# HIV incidence in a multinational cohort of men and transgender women who have sex with men in sub-Saharan Africa: Findings from HPTN 075

**DOI:** 10.1371/journal.pone.0247195

**Published:** 2021-02-25

**Authors:** Theodorus G. M. Sandfort, Yamikani Mbilizi, Eduard J. Sanders, Xu Guo, Vanessa Cummings, Erica L. Hamilton, Victor Akelo, Ravindre Panchia, Karen Dominguez, Michael J. Stirratt, Wairimu Chege, Jonathan Lucas, Charlotte A. Gaydos, Ying Q. Chen, Susan H. Eshleman

**Affiliations:** 1 HIV Center for Clinical and Behavioral Studies, New York State Psychiatric Institute and Columbia University, New York, New York, United States of America; 2 College of Medicine—Johns Hopkins Project, Blantyre, Malawi; 3 Kenya Medical Research Institute-Wellcome Trust Research Programme, Kilifi, Kenya; 4 Department of Medicine, University of Oxford, Oxford, England, United Kingdom; 5 Vaccine and Infectious Disease Division, Fred Hutchinson Cancer Research Center, Seattle, Washington, United States of America; 6 Department of Pathology, Johns Hopkins University School of Medicine, Baltimore, Maryland, United States of America; 7 Science Facilitation Department, FHI 360, Durham, North Carolina, United States of America; 8 Kenya Medical Research Institute, Kisumu Clinical Research Site, Kisumu, Kenya; 9 Perinatal HIV Research Unit, Univ. of the Witwatersrand, Soweto HPTN CRS, Soweto, South Africa; 10 Desmond Tutu HIV Centre, University of Cape Town Medical School, Cape Town, South Africa; 11 National Institute of Mental Health, Division of AIDS Research, Bethesda, Maryland, United States of America; 12 Division of AIDS, National Institute of Allergy and Infectious Diseases, National Institutes of Health, Bethesda, Maryland, United States of America; University of Cincinnati College of Medicine, UNITED STATES

## Abstract

Few studies have assessed HIV incidence in men who have sex with men (MSM) and transgender women (TGW) in sub-Saharan Africa (SSA). We assessed HIV incidence and its correlates among MSM and TGW in SSA enrolled in the prospective, multi-country HIV Prevention Trials Network (HPTN) 075 study, conducted from 2015 to 2017. Participants were enrolled at four sites in SSA (Kisumu, Kenya; Blantyre, Malawi; Cape Town and Soweto, South Africa). Eligible participants reported male sex assignment at birth, were 18 to 44 years of age, and had engaged in anal intercourse with a man in the preceding three months. Participation involved five study visits over 12 months. Visits included behavioral assessments and testing for HIV and sexually transmitted infections. Twenty-one of 329 persons acquired HIV during the study [incidence rate: 6.96/100 person-years (PY) (95% CI: 4.3, 10.6)]. Among TGW, HIV incidence was estimated to be 8.4/100 PY (95% CI: 2.3, 21.5). Four participants were found to have acute HIV infection at their first HIV-positive visit. HIV incidence varied among the four study sites, ranging from 1.3/100 PY to 14.4/100 PY. In multivariate longitudinal analysis, factors significantly associated with HIV acquisition were engagement in unprotected receptive anal intercourse [adjusted hazard ratio (AHR) 5.8, 95% confidence interval (CI): 2.4, 14.4] and incident rectal gonorrhea and/or chlamydia (AHR: 2.7, 95% CI: 1.1, 6.8). The higher HIV incidence in Cape Town compared to Blantyre could be explained by the higher prevalence of several risk factors for HIV infection among participants in Cape Town. Annual HIV incidence observed in this study is substantially higher than reported HIV incidence in the general populations in the respective countries and among MSM in the United States. Intensification of HIV prevention efforts for MSM and TGW in SSA is urgently needed.

## Introduction

Information about HIV incidence is critical for understanding the status of the HIV epidemic in diverse populations. Various studies have shown that men who have sex with men (MSM) in sub-Saharan Africa (SSA), as elsewhere in the world, are at increased risk for HIV infection [1,2]. Observed HIV prevalence in this population ranges from 7.8% in Khartoum, Sudan to 49.5% in Johannesburg, South Africa [3–8]. HIV incidence estimates for MSM in SSA range from 5.0/100 person-years (PY) (95% confidence interval [CI]: 2.5, 10.0) in three West African countries [9] to 15.4/100 PY (95% CI: 12.3, 19.0) in urban Nigeria [10]. Variations in these estimates reflect real population differences as well as differences in study design, such as eligibility criteria. Prior HIV incidence estimates for MSM in Kenya and South Africa range from 5.1/100 PY (95% CI: 2.6, 9.8) to 12.5/100 PY (95% CI: 8.1, 19.2) [6,11–13]. Incidence data for MSM in Malawi were not available prior to the current study. Reported HIV prevalence among transgender women (TGW) included in some of these studies, was significantly higher than among MSM [13–16]. One of the few incidence studies that included TGW in SSA observed an incidence among TGW in coastal Kenya of 20.6/100 PY (95% CI: 6.6, 63.8) [13].

The HIV epidemic among MSM and TGW in SSA is strongly affected by the legal and social context. Same-sex sexuality is criminalized in most SSA countries, including Kenya and Malawi, with penalties ranging up to death [17]. While sexual orientation is protected by the constitution in South Africa, same-sex sexuality remains highly stigmatized as in other countries in SSA, with social acceptance among the lowest in the world [18]. Experiences with homophobia, including violence and blackmail, are well-documented in this population [7,19]. Stigma associated with same-sex sexuality and gender diversity impacts HIV risk and limits access to HIV prevention and care. In prior studies of MSM and TGW in SSA, stigma was associated with a lack of access to condoms and lubricants, HIV testing, and antiretroviral treatment (ART), and with lower country-level investment in HIV-related services for sexual and gender minorities [20–23].

The current study, HIV Prevention Trials Network (HPTN) 075, was a prospective, multi-country HIV prevention and care study that evaluated the feasibility of recruiting and retaining a cohort of adult MSM and TGW (2015–2017) over a one-year period with five study visits. The study was conducted at four sites: Kisumu, Kenya; Blantyre, Malawi; Cape Town and Soweto, South Africa. At these sites, the observed HIV prevalence among those screened for study participation was 16.6%, 22.1%, 31.7%, and 49.1%, respectively [16]. Prior HIV incidence estimates for MSM in Kenya and South Africa ranged from 5.1/100 PY (95% CI: 2.6, 9.8) to 12.5 PY (95% CI: 8.1, 19.2) [6,11–13]. Incidence data for MSM in Malawi were not available prior to this study. Here, we report HIV incidence among the MSM and TGW enrolled in HPTN 075 and its correlates.

## Methods

### Study design and population

Each study site developed site-specific strategies to promote study awareness, acceptability, and recruitment in consultation with the local MSM community and other community stakeholders, taking advantage of each local community’s expertise. Sites implemented various recruitment strategies, including: (a) peer outreach: MSM were hired and trained as peer outreach workers; (b) participant referral: eligible participants were asked to encourage their friends to participate in the study (this activity was not incentivized); (c) informational sessions about the study, including presentations in gay-friendly places; (d) key informant referral; and (e) indirect recruitment: distribution of announcements via actual and virtual gay venues and events.

Main eligibility criteria included: (a) reporting male sex assignment at birth; (b) 18–44 years old; (c) reporting at least one act of anal intercourse in the previous three months with a person reported by the participant to be biologically male; and (d) willing to undergo HIV testing throughout the study and receive test results. Persons who had ever participated in a biomedical and/or behavioral intervention or cohort study for HIV or sexually transmitted infections (STIs) were excluded; co-enrollment in such studies was not permitted. The number of enrolled participants with HIV infection was capped at 20 per site; this was done to avoid excluding specific groups from the respective communities, while still enrolling a sufficient number of uninfected participants to address the study’s main goals, which were focused on primary HIV prevention. Persons with HIV who reported already being in HIV care or taking ART were excluded to be able to assess engagement in HIV care. This report includes evaluation of participants who tested negative for HIV infection at study enrollment.

These eligibility criteria permitted the enrollment of TGW as well as MSM. Although it is important to distinguish the health needs of TGW from those of MSM [24,25], TGW were included based on community input and a desire to be inclusive and respectful of how local communities define themselves. Some TGW in SSA identify as gay or see themselves as part of gay communities [26]. This integration may be similar to the early stages in the history of the transgender movement in the United States, when there were no clear boundaries between non-heteronormative gender and sexual groups, and some transgender persons identified as “gay” or saw themselves as an integral part of the gay community [27]. Inclusion of TGW further allowed for collection of exploratory information about this high-risk population in SSA. There were no specific efforts to recruit TGW.

### Procedures

Given potential risks associated with study participation, research staff developed site-specific risk-mitigation plans, guided by international guidelines for HIV prevention trials [28,29], ethical direction provided by the HPTN [30], and guidance for conducting research with MSM in rights-constrained environments [31]. The latter was intended to help researchers and community organizations safely conduct research on HIV among MSM in challenging social, political, and human rights contexts. This process was facilitated by a checklist of factors to be considered in design, conduct, and implementation of such studies [31]. Key variables in this checklist included: (a) active involvement of the MSM community in all aspects of the planned study; (b) identification and involvement of community stakeholders and government representatives; (c) inclusion of research on human rights protections/violations within the research context; (d) development of policies for dealing with hostile/intrusive media; (e) conduct of formative research including on community needs, criminalization, stigma and discrimination; (f) planning for MSM/LGBT community capacity-building and informed participation; and (g) planning for dissemination of findings involving the MSM/LGBT community and skill-building for activists to disseminate/use data locally for advocacy.

The screening visit included administrative procedures, real time HIV testing and collection of blood for plasma storage. Eligible participants subsequently had an enrollment visit, followed by visits at Weeks 13, 26, 39 and 52. All study visits included structured behavioral assessments and assessment of social impacts (S1 Questionnaire). Both were conducted using in-person interviews by research staff specifically trained to administer the survey. Participants had an option to complete short, sensitive parts of the survey themselves, if they preferred so. HIV testing and medical examinations were conducted at each visit. Biological samples were collected as follows: urine for *Neisseria gonorrhea* (GC) and/or *Chlamydia trachomatis* (CT) testing at Weeks 1, 26, and 52; pharyngeal and rectal swabs for GC/CT testing at Weeks 1 and 52; blood for hepatitis B virus (HBV) serology (HBSAb, HBSAg, and HBCoreAb) testing at Week 1 and for syphilis testing at Weeks 1, 26, and 52 (see S1 Table. Site assays). All participants received HIV risk reduction counseling; condoms and lubricants were available at each visit. Referrals for pre-exposure prophylaxis (PrEP) were made once it became available at each of the three of the sites. If PrEP became available through demonstration projects, the study protocol was updated to reflect this. Participants who acquired HIV during the study were referred for treatment according to country-specific protocols. Referrals were also made in cases where participants needed care that could not be delivered at the study site. Research participation was compensated according to local standards (ranging from $4 to $10 US per visit).

Demographic, psychosexual, and behavioral characteristics were assessed using tools that were successfully used in studies of MSM in SSA. Medical circumcision status was assessed verbally by asking participants: “Are you circumcised? By circumcised, I mean that the foreskin of your penis is removed”; if participants answered “yes,” they were asked if they had had a medical circumcision, performed by a doctor in a clinic or hospital, or a traditional circumcision.

Gender identity was determined by asking “How do you identify your gender?”; this question was preceded by an explanation that “gender is the social part of being male or female. It relates to your self-identity. When I ask about gender, I am asking about whether you regard yourself to be male, female, transgender female, or if you identify yourself in another way”. Persons who identified as “female” or “transgender” or in ways other than exclusively male, were categorized as transgender.

Experiences with transactional sex were assessed with the question, “Sometimes people give or receive something in return for having sex. This can be a variety of things, including food, clothes, a place to sleep, a cell phone, money and a lot of other things. Has a man ever given you something in exchange for sex?” Whether participants had ever experienced forced sex was assessed by asking, “Has a man ever forced you to have sex when you did not want to yourself? This man could have been a stranger, someone you knew, or a regular partner”.

Whether participants had any experiences with homophobia in the preceding year was based on responses to five yes/no questions, such as “Have you, as a result of sexual orientation or practice, in last year been verbally or physically harassed?” [32]. Based on the distribution of responses and to facilitate interpretation, scores were dichotomized as “none” or “any” homophobic experiences in the preceding year. Four items were adapted from [33,34] to assess participants’ negative feelings about same-sex sexuality (e.g., “I cannot decide whether I am bisexual, homosexual, or heterosexual” and “Sometimes I dislike myself for being a man who has sex with other men”). Statements were scored on a 4-point Likert scale (1 = strongly agree, 4 = strongly disagree). The Cronbach alpha for this assessment in this study was .75. A mean score was calculated. For the current analysis, a median split was used to dichotomize the variable, with 2 as the median. Concealment of homosexuality was assessed by asking “In general, how hard do you try to keep your sexual orientation hidden from your family?” (1 = “I openly talk about it with my family” to 4 = “Try very hard”) [35].

Sexual behavior was assessed at each visit by a member of the research staff. To assess recent sexual behavior, participants were first asked the number of persons they had sex with in the preceding three months. For each partner they were asked how many times they engaged in receptive and insertive anal sex. Participants were also asked about the frequency of condom use for receptive and insertive anal intercourse with each partner. These data were collected for up to three partners. These questions were embedded in a set of other questions about each specific sexual partnership. Answers to these questions were used to calculate the number of times a participant had engaged in condomless receptive and insertive anal sex, respectively, and the number of male partners with whom they had condomless receptive and insertive anal sex. Participants were also asked how often they had sex with each partner under the influence of alcohol or drugs. Initiation of PrEP was assessed once PrEP became available at a study site; although several PrEP referrals were made, no participants reported having initiated PrEP.

### Laboratory testing

Laboratory testing was performed at study sites and at the HPTN Laboratory Center. A list of the assays used for real-time testing is provided in S1 Table. HIV testing was performed at screening, enrollment, and all subsequent study visits. The testing algorithm at screening included two HIV rapid tests or a rapid test and a second HIV screening test; one or both of these tests were performed in a laboratory. A single HIV rapid test was performed at enrollment. HIV RNA testing was also performed if the person had symptoms of acute HIV infection. If all HIV tests at screening and enrollment were non-reactive/negative, the participant was eligible for enrollment as a participant without HIV infection. At follow-up visits, testing was performed using the same algorithm used at the screening visit. Further testing was performed to determine HIV status if any of these tests were reactive/positive. If testing at any follow-up visit indicated that the participant acquired HIV infection, a separate visit was conducted 1–2 weeks later to confirm HIV acquisition and rule out the possibility of a participant/sample mix-up; CD4 cell count and HIV viral load testing were also performed at that visit.

Additional retrospective testing was performed for quality assurance at the HPTN Laboratory Center (Johns Hopkins University School of Medicine, Baltimore, MD) where all enrollment samples were tested with the 4^th^ generation Architect HIV-1 Ag/Ab test (Architect test, Abbott Laboratories, Wiesbaden, Germany) to confirm enrollment HIV status; further testing was performed to confirm HIV status at enrollment in participants with reactive test results. The following additional tests were performed for all seroconversion cases: the Architect test; the 4^th^ generation BioRad GS HIV Combo Ag/Ab EIA (BioRad test, Bio-Rad Laboratories, Hercules, CA); the Geenius HIV ½ Supplemental Assay (Geenius test, Bio-Rad Laboratories); and the APTIMA HIV-1 RNA Qualitative Assay (APTIMA test; Hologic Gen-Probe Inc., San Diego, CA); the APTIMA test was also performed at the last HIV negative visit to determine if the participant had acute HIV infection at that visit. HIV viral load testing was performed at the first HIV positive visit using the RealTime HIV-1 Viral Load Assay (Abbott Molecular, Des Plaines, IL).

STI testing was performed at enrollment, Week 26, and Week 52, for GC and CT using nucleic acid amplification tests for urine, pharyngeal swabs, and rectal swabs. Testing for GC/CT in urine was performed at study sites; swab testing was performed at the HPTN Laboratory Center. At these visits, participants were also tested for syphilis using blood specimens according to local standards using treponemal tests and non-treponemal tests. Syphilis status at enrollment was determined based on review of test results obtained at all three study visits (participants were classified as being positive for syphilis at enrollment if both the treponemal and the non-treponemal tests were positive. At follow-up visits, participants who had active syphilis at enrollment were classified as not successfully treated if both tests were still positive, or successfully treated if only the treponemal test was positive, or if both tests were positive with a drop in the non-treponemal titer of two or more dilutions). At enrollment, HBV testing was performed, including testing for hepatitis B surface antigen (HBsAg), testing for antibodies to hepatitis B surface antigen (HBsAb), and testing for antibodies to hepatitis B core antigen (HBcAb) tests. A positive status includes active infection (acute and chronic, HBsAg+, anti-HBc+, anti-HBs negative) and excludes resolved HBV infections (HBsAg negative, anti-HBc+, anti-HBs+) and vaccinated persons (HBSAg negative, anti-HBc negative).

### Statistical analysis

Univariate and multivariate longitudinal Cox regression analysis were used to estimate HIV incidence and identify baseline and time-dependent correlates of incident infection. A stepwise procedure was used for the multivariate analysis with p < 0.10 as criterion for entry and p > 0.10 as criterion for elimination of predictor variables in model building. Because HIV incidence differed significantly between two study sites (Blantyre and Cape Town), we used Chi-square tests to detect differences in sociodemographic characteristics, risk behaviors, and STI history between those sites. Analyses were conducted using SAS 9.4.

### Ethics statement

Participating study sites received approval from their respective institutional review boards (IRBs): Kenya Medical Research Institute (KEMRI; #2994; Nairobi, Kenya); College of Medicine Research Ethics Committee (COMREC; 07/15/1762; Blantyre, Malawi) and Institutional Review Board, Johns Hopkins University School of Public Health (00006252; Baltimore, USA); Human Research Ethics Committee, University of Cape Town, Faculty of Health Sciences (#795/2014; Cape Town, South Africa); Research Ethics Committee (Medical), University of the Witwatersrand Human (Wits HREC Medical; #141105; Johannesburg, South Africa). Additional approval was received from the Institutional Review Board, New York State Psychiatric Institute (#6868; New York, USA). Informed consent was obtained separately for screening and enrollment. Participants provided written consent at three sites. At one site, the local IRB recommended the use of verbal consent, because signing the consent form would create an unnecessary documentation of study participation that could lead to unintended disclosure; participants’ verbal consent was documented by the research staff. Participants were compensated for participation in the study according to local standards. If a potential participant was found to be illiterate, study staff identified an impartial witness to take part in the informed consent process as an added protection for the participant.

## Results

### Study population

The analyses in this report included 329 MSM and TGW who tested negative for HIV infection at enrollment (Table 1); this represents 82.0% of 401 participants enrolled. Of these 329 persons, 4 persons died (unrelated to the study) and 302 (302/325; 92.9%) completed the final study visit.

**Table 1:** Sociodemographic characteristics, risk behaviors and sexually transmitted infections among men who have sex with men and transgender women who tested negative for HIV infection at study enrollment, HPTN 075 (N = 329).

Characteristic		N*	%
Study site	Blantyre, Malawi	83	25.2
	Kisumu, Kenya	85	25.8
	Cape Town, South Africa	80	24.3
	Soweto, South Africa	81	24.6
Age at baseline (years)	18–24	219	66.6
	25–44	110	33.4
Level of education	Less than secondary	113	34.9
	Completed secondary	138	42.6
	Some tertiary	73	22.5
Circumcision status (medical)	No	192	58.7
	Yes	135	41.3
Gender identity	Cisgender	271	83.6
	Transgender	53	16.4
Sexual attraction	Men only	144	44.2
	Men and women	182	55.8
Sexual identity	Gay	197	60.2
	Bisexual	130	39.8
Sex with women (ever)	No	128	39.0
	Yes	200	61.0
Transactional sex (ever)	No	235	72.5
	Yes	89	27.5
Forced sex (ever)	No	246	75.2
	Yes	81	24.8
Homophobic experiences	None	181	55.4
	Any	146	44.6
Negative feelings about same-sex attraction	Few	186	57.1
	Many	140	42.9
Concealment of sexual orientation	Low	178	55.6
	High	142	44.4
*In 3 months preceding enrollment*:			
Number of sexual partners	0–1	176	54.2
	2	95	29.3
	3+	54	16.6
Number of times participant had CRAI with a man	0	247	75.5
	1+	80	24.5
Number of men with whom participant had CRAI	0	247	75.5
	1	67	20.5
	2+	13	4.0
Number of times participant had CIAI with man	0	250	76.5
	1+	77	23.6
Number of men with whom participant had CIAI	0	250	76.5
	1+	56	17.1
	2+	21	6.4
Number of sexual partners while under the influence of alcohol or drugs	0	162	49.5
	1+	165	50.5
*STI at enrollment*:			
Rectal gonorrhea and/or chlamydia	No	276	83.9
	Yes	53	16.1
Oral gonorrhea and/or chlamydia	No	321	97.6
	Yes	8	2.4
Urethral gonorrhea and/or chlamydia	No	309	93.9
	Yes	20	6.1
Syphilis	No	313	95.1
	Yes	16	4.9
HBV (chronic or acute)	No	273	83.0
	Yes	56	17.0

*N may not add to total due to missing values.

Abbreviations: SSA = sub-Saharan Africa; CRAI = condomless receptive anal intercourse; CIAI = condomless insertive anal intercourse; STI = sexually transmitted infection; HBV = Hepatitis B virus.

About one in six participants (16.4%; 53/324) reported identifying as female or transgender. The mean age of the 329 individuals was 23.8 years; 66.6% (219/329) were < 25 years old; 41.3% (135/327) reported having undergone medical circumcision. Over half of the participants (182/326; 55.8%) reported that they were sexually attracted to both men and women; 39.8% (130/327) identified as bisexual; and 61.0% (200/328) reported ever having had sex with a woman. About a quarter of the participants reported that they had ever engaged in transactional sex (89/324; 27.5%) or ever were forced to have sex (81/327; 24.8%). Less than half of the participants (146/327; 44.4%) reported having had any homophobic experiences in the last year. A similar proportion had negative feelings about their same-sex attraction and tried hard to conceal their sexual orientation [42.9% (140/326) and 44.4% (142/320), respectively]. The number of sexual partners reported in the three months before enrollment ranged from 0 to 10. About a quarter of the participants reported to have had condomless receptive anal sex with one or more men (80/327; 24.5%); condomless insertive anal sex was reported by 23.6% (77/327) of the participants (both in the three months preceding enrollment). About half of the participants (165/327; 50.5%) had had sex with one or more partners while under the influence of alcohol or drugs. The most frequently diagnosed STIs were rectal GC and/or CT (53/329; 16.1%). STIs were diagnosed in 26.1% of the participants (See S2 Table for STI incidence). Chronic and acute HBV were diagnosed in 17.0% (56/329) of the participants.

### HIV incidence

Overall, 21 (6.4%) of the 329 participants who tested negative for HIV infection at enrollment acquired HIV during the study, corresponding to an overall HIV incidence rate of 6.96/100 PY (95% CI: 4.3, 10.6). Retrospective testing at the HPTN Laboratory Center revealed that four of the 21 participants were found to have acute HIV infection at their first HIV-positive visit. All four cases had reactive results with two 4^th^ generation tests (the Architect test and the BioRad test) but had negative results with the Geenius test. All four cases had positive qualitative HIV RNA tests and high HIV viral loads (220,180; 1,453,100; 3,455,083; and >10,000,000 HIV RNA copies/mL). The rapid HIV tests used in this study detect HIV antibody only and would not be expected to detect acute (RNA only) infections. In three of the four cases, HIV infection was missed at the study site using two HIV screening tests, at least one of which was performed in a laboratory; in the fourth case, one of the two screening tests was reactive (the case with an HIV viral load of 3,455,083 copies/ml). In all three cases where infection was missed, the infection was detected at the sites by HIV rapid testing at the next quarterly visit.

### Correlates of HIV acquisition

Four of the 21 incident HIV cases occurred among TGW, corresponding to an incidence estimate of 8.4/100 PY (95% CI: 2.3, 21.5). HIV incidence varied between study sites (Table 2). The incidence rate was significantly higher in Cape Town (14.4/100 PY) than in Blantyre (1.3/100 PY; unadjusted Hazard Ratio [UHR] 12.1; 95% CI: 1.5, 97.2). The UHR for HIV acquisition was greater for persons who reported having had condomless receptive anal intercourse one or more times (UHR: 7.5; 95% CI: 3.1, 17.8), persons who had one or who had two or more male partners with whom they had unprotected receptive anal sex (compared to persons who reported no unprotected receptive anal sex; 27.5/100 PY, 31.7/100 PY, 3.8/100 PY, respectively; UHR: 7.3, 95% CI: 3.0, 17.6 and UHR: 10.5, 95% CI: 1.35, 83.0, respectively), and for participants who were diagnosed with rectal GC and/or CT (HR: 4.2; 95% CI: 1.8, 10.1). In univariate analysis, none of the other risk factors or demographic characteristics was associated with HIV acquisition. In multivariate analysis, having had condomless receptive anal intercourse one or more times and rectal GC and/or CT incidence were significantly associated with HIV acquisition (adjusted HR [AHR] engagement in unprotected receptive anal intercourse: 5.8; 95% CI: 2.4, 14.4; and AHR for rectal GC and/or CT: 2.7; 95% CI: 1.1, 6.8).

**Table 2:** Association of HIV incidence with sociodemographic characteristics, risk behaviors, and sexually transmitted infections at baseline among men who have sex with men and transgender women, HPTN 075 (N = 329)^1^.

Characteristic		HIV infections/person years	HIV incidence per 100 PY	Unadjusted HR (95% CI)	Adjusted HR (95% CI)
Study site	Blantyre, Malawi	1/74.4	1.3	REF	
	Kisumu, Kenya	3/80.1	3.8	3.0 (0.3, 29.0)	
	Cape Town, South Africa	10/69.3	14.4	12.1 (1.5, 97.2)	
	Soweto, South Africa	7/78.1	9.0	7.4 (0.9, 62.0)	
Age at baseline (years)	18–24	15/204.5	7.3	REF	
	25–44	6/97.3	6.2	0.9 (0.3, 2.2)	
Level of education	Less than secondary	7/102.9	6.8	REF	
	Completed secondary	9/126.1	7.1	1.0 (0.4, 2.7)	
	Some tertiary	5/67.8	7.4	1.0 (0.3, 3.3)	
Circumcision status (medical)	No	15/173.1	8.7	REF	
	Yes	6/126.6	4.7	0.6 (0.2, 1.5)	
Gender identity	Cisgender	17/249.2	6.8	REF	
	Transgender	4/47.7	8.4	1.2 (0.4, 3.6)	
Sexual attraction	Men only	12/138.8	8.7	REF	
	Men and women	9/160.8	5.6	0.7 (0.3, 1.6)	
Sexual identity	Gay	12/181.7	6.6	REF	
	Bisexual	9/118.6	7.6	1.2 (0.5, 2.8)	
Sex with women (ever)	No	11/119.7	9.2	REF	
	Yes	10/181.1	5.5	0.6 (0.3, 1.5)	
Transactional sex (ever)	No	15/213.8	7.0	REF	
	Yes	6/83.0	7.2	1.0 (0.4, 2.7)	
Forced sex (ever)	No	18/220.7	8.2	REF	
	Yes	3/79.0	3.8	0.5 (0.1, 1.5)	
Homophobic experiences	None	16/234.9	6.8	REF	
	Any	5/66.4	7.5	1.1 (0.4, 2.9)	
Negative feelings about same-sex attraction	Few	11/177.9	6.2	REF	
	Many	10/121.6	8.2	1.3 (0.6, 3.1)	
Concealment of sexual orientation	Low	9/160.0	5.6	REF	
	High	11/133.5	8.1	1.5 (0.6, 3.6)	
HBV (chronic or acute)	No	15/253.0	6.0	REF	
	Yes	6/48.8	12.3	2.1 (0.8, 5.5)	
**Time-dependent correlates**					
Number of sexual partners	0–1	14/203.9	6.9	REF	
	2	2/69.2	2.9	0.4 (0.1, 1.9)	
	3+	5/28.7	17.4	2.5 (0.9, 7.1)	
Number of times participant had URAI with a man	0	10/262.2	3.8	REF	REF
	1+	11/39.5	27.8	7.5 (3.1, 17.8)	5.8 (2.4, 14.4)
Number of men with whom participant had URAI	0	10/262.2	3.8	REF	
	1	10/36.4	27.5	7.3 (3.0, 17.6)	
	2+	1/3.2	31.7	10.5 (1.3, 83.0)	
Number of times participant had UIAI with man	0	18/268.8	6.7	REF	
	1+	3/32.9	9.1	1.3 (0.4, 4.6)	
Number of men with whom participant had UIAI	0	18/268.8	6.7	REF	
	1	2/29.2	6.9	1.0 (0.2, 4.4)	
	2+	1/3.8	26.5	3.9 (0.5, 29.2)	
Number of sexual partners while under influence of alcohol or drugs	0	13/211.7	6.1	REF	
	1+	8/90.1	8.9	1.5 (0.6, 3.6)	
Rectal gonorrhea and/or chlamydia	No	12/256.8	4.7	REF	REF
	Yes	9/44.2	20.4	4.2 (1.8, 10.1)	2.7 (1.1, 6.8)
Oral gonorrhea and/or chlamydia	No	19/294.1	6.5	REF	
	Yes	1/6.7	15.0	2.4 (0.3, 17.8)	
Urethral gonorrhea and/or chlamydia	No	19/284.8	6.7	REF	
	Yes	2/17.0	11.8	1.8 (0.4, 7.7)	
Syphilis	No	19/286.0	6.7	REF	
	Yes	2/15.9	12.6	2.1 (0.5, 8.9)	

Abbreviations: SSA = sub-Saharan Africa; PY = person years; HR = hazard ratio; REF = reference group; CI: confidence interval; CRAI = condomless receptive anal intercourse; CIAI = condomless insertive anal intercourse; STI = sexually transmitted infection; HBV = Hepatitis B virus.

^1^ A stepwise Cox Model was used with p-value < 0.1 for entry and *p* > .01 for exit of variables in the multivariable analysis.

### Differences between study sites

Participants at the lowest HIV incidence site (Blantyre) differed from participants at the highest HIV incidence site (Cape Town) in several ways (Table 3). Participants in Blantyre were more likely to be attracted to both men and women (75.3% vs. 40.0% in Cape Town); to identify as bisexual instead of gay (52.4% vs. 31.3% in Cape Town); and to ever have had sex with women (76.8% vs. 53.8% in Cape Town). Participants in Blantyre were also more likely to have engaged in condomless insertive anal sex, both in terms of number of times (32.9% reported one or more times condomless insertive anal sex in the three months prior to enrollment. compared to 18.8% in Cape Town) and number of partners (11.0% reported condomless insertive anal sex with two or more sexual partners at least once in the three months prior to enrollment vs. 2.5% in Cape Town). Participants in Blantyre were less likely to have had three or more sexual partners in the three months before enrollment (6.1% vs. 23.1% in Cape Town) and were less likely to be diagnosed with rectal GC and/or CT at enrollment (7.2% vs. 23.8% in Cape Town) and syphilis (1.2% vs. 12.5% in Cape Town).

**Table 3:** Comparison of sociodemographic characteristics, risk behaviors and sexually transmitted infections among men who have sex with men and transgender women in Blantyre, Malawi and Cape Town, South Africa, who tested negative for HIV infection at study enrollment, HPTN 075 (N = 163).

Characteristic		Blantyre Malawi	Cape Town South Africa	Chi square	*P*
Age at baseline	18–24	48/83 (57.8%)	54/80 (67.5%)	1.63	0.202
	25–44	35/83 (42.2%)	26/80 (32.5%)		
Level of education	Less than secondary	39/80 (48.8%)	31/80 (38.8%)	4.11	0.128
	Completed secondary	30/80 (37.5%)	28/80 (35.0%)		
	Some tertiary	11/80 (13.8%)	21/80 (26.3%)		
Circumcision status (medical)	No	61/82 (74.4%)	68/80 (85.0%)	2.81	0.094
	Yes	21/82 (25.6%)	12/80 (15.0%)		
Gender identity	Cisgender	62/81 (76.5%)	68/80 (85.0%)	1.85	0.174
	Transgender	19/81 (23.5%)	12/80 (15.0%)		
Sexual attraction	Men only	20/81 (24.7%)	48/80 (60.0%)	20.57	**<0.001**
	Men and women	61/81 (75.3%)	32/80 (40.0%)		
Sexual identity	Gay	39/82 (47.6%)	55/80 (68.8%)	7.46	**0.006**
	Bisexual	43/82 (52.4%)	25/80 (31.3%)		
Sex with women (ever)	No	19/82 (23.2%)	37/80 (46.3%)	9.54	**0.002**
	Yes	63/82 (76.8%)	43/80 (53.8%)		
Transactional sex (ever)	No	59/80 (73.8%)	66/80 (82.5%)	1.79	0.181
	Yes	21/80 (26.3%)	14/80 (17.5%)		
Forced sex (ever)	No	57/82 (69.5%)	63/80 (78.8%)	1.80	0.180
	Yes	25/82 (30.5%)	17/80 (21.3%)		
*In 3 months preceding enrollment*:					
Number of sexual partners	0–1	48/82 (58.5%)	40/78 (51.3%)	9.63	**0.008**
	2	29/82 (35.4%)	20/78 (25.6%)		
	3+	5/82 (6.1%)	18/78 (23.1%)		
Number of times participant had CRAI with a man	0	65/82 (79.3%)	55/80 (68.8%)	2.33	0.127
	1+	17/82 (20.7%)	25/80 (31.3%)		
Number of men with whom participant had CRAI	0	65/82 (79.3%)	55/80 (68.8%)	4.64	0.098
	1	16/82 (19.5%)	19/80 (23.8%)		
	2+	1/82 (1.2%)	6/80 (7.5%)		
Number of times participant had CIAI with a man	0	55/82 (67.1%)	65/80 (81.3%)	4.24	**0.040**
	1+	27/82 (32.9%)	15/80 (18.8%)		
Number of men with whom participant had CIAI	0	55/82 (67.1%)	65/80 (81.3%)	6.07	**0.048**
	1	18/82 (22.0%)	13/80 (16.3%)		
	2+	9/82 (11.0%)	2/80 (2.5%)		
Number of sexual partners while under the influence of alcohol or drugs	0	49/82 (59.8%)	37/80 (46.3%)	2.97	0.085
	1+	33/82 (40.2%)	43/80 (53.8%)		
*STIs at enrollment*:					
Rectal gonorrhea and/or chlamydia	No	77/83 (92.8%)	61/80 (76.3%)	8.56	**0.003**
	Yes	6/83 (7.2%)	19/80 (23.8%)		
Oral gonorrhea and/or chlamydia	No	82/83 (98.8%)	78/80 (97.5%)	0.38	0.539
	Yes	1/83 (1.2%)	2/80 (2.5%)		
Urethral gonorrhea and/or chlamydia	No	78/83 (94.0%)	78/80 (97.5%)	1.23	0.267
	Yes	5/83 (6.0%)	2/80 (2.5%)		
Syphilis	No	82/83 (98.8%)	70/80 (87.5%)	8.26	**0.004**
	Yes	1/83 (1.2%)	10/80 (12.5%)		
HBV	No	66/83 (79.5%)	69/80 (86.3%)	1.30	0.255
	Yes	17/83 (34.9%)	11/80 (13.8%)		

*N may not add to total due to missing values.

**P values <0.05 are bolded.

Abbreviations: CRAI = condomless receptive anal intercourse; CIAI = condomless insertive anal intercourse; STIs = sexually transmitted infections; HBV = Hepatitis B virus.

## Discussion

HPTN 075 demonstrated that research with MSM and TGW could achieve excellent recruitment and one-year retention at four sites in three countries in SSA, with more than 90% of the cohort completing the final assessment. Overall HIV incidence in the cohort was 6.96/100 PY. HIV incidence varied by study site; the incidence observed in Blantyre was significantly lower than the incidence observed in Cape Town. Diagnosis of HBV at enrollment and incident rectal GC and/or CT were independently associated with HIV acquisition.

The observed HIV incidence is consistent with reports of HIV incidence among MSM and TGW in other studies conducted in SSA [6,9–11]. Kimani et al. observed an overall incidence of 5.1/100 PY (95% CI: 2.6, 9.8) among MSM and TGW in coastal Kenya, including a high incidence among TGW of 20.6/100 PY (95% CI: 6.6, 63.8) [13]. Lane et al. observed an incidence of 12.5/100 PY (95% CI: 8.1, 19.2) among MSM in Mpumalanga, South Africa [12]. The observed HIV incidence estimates are substantially higher than the ones reported for the general populations in the respective countries (0.12%, 0.24%, and 0.55% for Kenya, Malawi, and South Africa, respectively) [36]. Compared with the incidence observed among Black MSM in the United States, the observed HIV incidence in this study is substantially higher [37]. Matthews et al. calculated a weighted mean incidence of 4.16% per year (95% CI:2.76, 5.56) across six studies of Black MSM in the United States [38]. When comparing the various estimates, it is important to note that participants in HPTN 075 were not selected based on frequent engagement in high-risk sexual practices; HIV incidence would be higher among populations selected for being at high HIV risk. The high observed HIV incidence in the context of relatively low numbers of sexual partners suggests that MSM and TGW in these settings are at risk because of sex with partners who have undiagnosed and/or unsuppressed HIV infection. This highlights the importance of reaching key populations and engaging them in HIV testing and HIV care, and prevention.

Study findings about correlates of HIV acquisition confirm prior studies among MSM in SSA. As in other studies, engagement in receptive anal intercourse was associated with HIV acquisition [6,10,11]. In this study, engagement in unprotected receptive anal intercourse and incident rectal GC and/or CT were the only factors independently associated with HIV acquisition. That we did not find additional factors may reflect the limited sample size and the resulting reduced power. Bacterial STIs have been associated with HIV acquisition in other studies of MSM, including in the iPrEx trial [10,39].

The lower HIV incidence observed among MSM and TGW in Blantyre compared to Cape Town is striking. Several risk factors for HIV infection were more prevalent among participants in Cape Town: exclusive homosexual attraction, identification as gay vs. bisexual, and never having had sexually engaged with women; these factors have consistently been shown to be associated with higher HIV prevalence and incidence [6,11]. Even though participants in Blantyre were more likely to have engaged in condomless insertive anal intercourse, participants in Cape Town had more sexual partners in the preceding three months. Participants in Cape Town were also more likely to be diagnosed with an STI (rectal GC and/or CT, or syphilis) at enrollment. The difference in HIV prevalence between Cape Town and Blantyre observed at screening (31.7% and 22.1%, respectively) [16] might also have contributed to the difference in HIV incidence. This difference is consistent with the higher estimated overall HIV prevalence reported among MSM in South Africa and Malawi (26.8% and 17.3%) [36] and among 15- to 49-year-old men in South Africa and Malawi (18.9% and 9.2%, respectively) [40].

In four of the 21 cases with incident HIV, the participant had acute infection at the first HIV positive visit. The HIV viral loads were high in all four of cases (220,180 to >10,000,000 copies/mL). In three of the four cases, HIV infection was missed by testing with two HIV screening tests and was not detected until the next quarterly visit. This provided a 3-month window where those individuals were likely to be highly infectious, unaware of their HIV-positive status, and not on treatment. This highlights the need to use sensitive 4^th^ (or 5^th^) generation HIV tests to accurately screen populations at high risk for HIV infection.

Interpretation of these findings should take into account important study limitations. First, because of the small sample size and relatively limited number of incident HIV cases, we had limited power to identify correlates of HIV acquisition. Second, it is not clear to what extent loss to follow-up may have affected the HIV incidence rate. Third, because the behavioral variables are based on self-report, they may have been affected by social desirability bias. Finally, because of the methods used to recruit participants in HPTN 075 and the enrollment criteria used in the study, the sample is not fully representative and observed incidence cannot be generalized to all MSM and TGW in SSA.

The observed HIV incidence among MSM and TGW in Kenya, Malawi, and South Africa suggests a strong need to intensify HIV prevention efforts among this population in SSA at multiple levels. Given the association between incident HIV and STIs, efforts to advance targeted STI detection and treatment, especially for extra genital STIs, could play a role in curbing HIV transmission. Behavioral interventions that promote PrEP uptake and adherence would be beneficial [41]. Community-based interventions (e.g., promotion of social capital and a sense of belonging), might lead to a decrease in risk behaviors and HIV acquisition [42]. Structural interventions that reduce sexual and gender-related stigma and discrimination may also help in these populations. Existing barriers to care for MSM and TGW living with HIV should be eliminated [23]. This is critical for the health and well-being of persons living with HIV and for reducing onward HIV transmission. Further research is needed to improve our understanding of the differences between the HIV epidemics among MSM and TGW in various geographic areas and to identify the most efficient ways to prevent HIV transmission in these populations.

## Supporting information

S1 Table Site assays. (PDF)

S2 Table STI diagnoses by study visit. (PDF)

S3 Table STI diagnoses by study visit. (PDF)

S1 Questionnaire Enrollment and follow-up questionnaires. (PDF)

S1 Data Dataset. (CSV)

S1 File (DOCX)
