# Correction: HIV incidence in a multinational cohort of men and transgender women who have sex with men in sub-Saharan Africa: Findings from HPTN 075

**DOI:** 10.1371/journal.pone.0249331

**Published:** 2021-03-24

**Authors:** The PLOS ONE Staff

## Notice of republication

An incorrect version of the Data Availability statement was published in error. The publisher apologizes for this error. Additional text was missing from affiliation 7. The correct affiliation 7 is: Science Facilitation Department, FHI 360, Durham, North Carolina, United States of America. This article was republished on March 8, 2021 to correct for this error. Please download this article again to view the correct version.

## References

[1] Sandfort TGM, Mbilizi Y, Sanders EJ, Guo X, Cummings V, Hamilton EL, et al. (2021) HIV incidence in a multinational cohort of men and transgender women who have sex with men in sub-Saharan Africa: Findings from HPTN 075. PLoS ONE 16(2): e0247195. 10.1371/journal.pone.0247195 33630925 PMC7906338

