# Supplementary material for: HIV incidence in a multinational cohort of men and transgender women who have sex with men in sub-Saharan Africa: Findings from HPTN 075
Source: PLoS One. 2021 Feb 25;16(2):e0247195. doi: 10.1371/journal.pone.0247195 (PMC7906338; doi:10.1371/journal.pone.0247195)
Supplement: S1 Table — (PDF) [file pone.0247195.s001.pdf]

	Malawi		Kenya		Cape Town		Soweto	
	Kit Name	Manufacturer	Kit Name	Manufacturer	Kit Name	Manufacturer	Kit Name	Manufacturer
**Serology**								
HIV rapid test (FDA approved)	OraQuick Advance Rapid HIV 1/2 Antibody Test	OraSure Technologies	Uni-Gold Recombigen HIV-1/2 Test	Trinity Biotech	Uni-Gold Recombigen HIV-1/2 Test	Trinity Biotech	Uni-Gold Recombigen HIV-1/2 Test	Trinity Biotech
HIV rapid test	Determine HIV-1/2 Rapid Test	Alere	Determine HIV-1/2 Rapid Test	Alere	Determine HIV-1/2 Rapid Test	Alere	Determine HIV-1/2 Rapid Test	Alere
Hep B sAg	GS HBsAg EIA 3.0	BIO-RAD	Murex HBsAg Version 3	DiaSorin	ARCHITECT HBsAg Qualitative II	Abbott Diagnostics	ARCHITECT HBsAg Qualitative II	Abbott Diagnostics
Hep B sAb	MONOLISA Anti-HBs EIA	BIO-RAD	Anti-HBs	DiaSorin	ARCHITECT Anti-HBsAb test	Abbott Diagnostics	ARCHITECT Anti-HBsAb test	Abbott Diagnostics
Hep B Core Ab	MONOLISA Anti-HBc EIA	BIO-RAD	Murex anti-HBc (Total)	DiaSorin	ARCHITECT Anti-HBc II test	Abbott Diagnostics	ARCHITECT Anti-HBc II test	Abbott Diagnostics
**Virology**								
HIV-1 RNA PCR	RealTime HIV-1 Viral Load Assay	Abbott Molecular	RealTime HIV-1 Viral Load Assay	Abbott Molecular	RealTime HIV-1 Viral Load Assay	Abbott Molecular	RealTime HIV-1 Viral Load Assay	Abbott Molecular
**STI Testing**								
Syphilis	BIOTEC RPR Test Kit	BIOTEC Laboratories Limited (UK)	BD Macro-Vue RPR Card Test	Becton Deckinson	BD Macro-Vue RPR Card Test	Becton Deckinson	BD Macro-Vue RPR Card Test	Becton Deckinson
Treponemal confirmatory	BIOTEC TPHA Test Kit	BIOTEC Laboratories Limited (UK)	SD Bioline Syphilis 3.0	Standard Diagnostic	SERODIA-TP-PA	Serodia - JAPAN	IMMUNTREP TPHA	Omega Diagnostics
CT/NG urine	Xpert CT/NG	Cepheid	Abbott RealTime CT/NG Amplification Reagent Kit	Abbott Molecular	Xpert CT/NG	Cepheid	Xpert CT/NG	Cepheid
CTNG rectal swab	Aptima Combo 2*	Hologic	Aptima Combo 2*	Hologic	Aptima Combo 2*	Hologic	Aptima Combo 2*	Hologic
CTNG pharyngeal swab	Aptima Combo 2*	Hologic	Aptima Combo 2*	Hologic	Aptima Combo 2*	Hologic	Aptima Combo 2*	Hologic

*Testing performed at the HPTN LC
