# Supplementary material for: HIV incidence in a multinational cohort of men and transgender women who have sex with men in sub-Saharan Africa: Findings from HPTN 075
Source: PLoS One. 2021 Feb 25;16(2):e0247195. doi: 10.1371/journal.pone.0247195 (PMC7906338; doi:10.1371/journal.pone.0247195)
Supplement: S2 Table — (PDF) [file pone.0247195.s002.pdf]

S3 Table. STI diagnoses by study visit

	Week 1	Week 3	Week 5
Rectal gonorrhea and/or chlamydia	16.7% (53/318)	-	12.3% (36/293)
Oral gonorrhea and/or chlamydia	2.5% (8/319)	-	2.0% (6/302)
Urethral gonorrhea and/or chlamydia	6.3% (20/319)	5.3% (16/304)	4.7% (14/301)
Syphilis	5.0% (16/319)	5.3% (16/304)	7.6% (23/304)
