# Supplementary material for: HIV incidence in a multinational cohort of men and transgender women who have sex with men in sub-Saharan Africa: Findings from HPTN 075
Source: PLoS One. 2021 Feb 25;16(2):e0247195. doi: 10.1371/journal.pone.0247195 (PMC7906338; doi:10.1371/journal.pone.0247195)
Supplement: S1 Questionnaire — (PDF) [file pone.0247195.s004.pdf]

S1 Questionnaire. Enrollment and Follow-up questionnaires

Local language versions of both questionnaires can be requested from the corresponding author.

HPTN 075 Enrollment Questionnaire Version 1.4 – 19OCT15

Contents

Demographic Background 3
Sexual and Gender Identity, and Social Support 7
Sexual History 11
Sexual Behavior Assessment 16
HIV/STI-Related Factors 36
HIV-Related Care and Treatment and HIV Stigma 41
Alcohol and Drugs 46
Mental Health 48
Experience of Study Participation 49

Self-Administered vs. Interviewer-Administered

Demographic Background	Interviewer Administered
Sexual and Gender Identity and Social Support	Interviewer Administered
Sexual History	Interviewer Administered
Sexual Behavior Assessment	Interviewer Administered
HIV/STI-Related Factors	Self-Administered HIVHERPES through HIVNOTRI, then back to Interviewer-Administered
HIV-Related Care and Treatment and HIV Stigma	Self-Administered resumes at CTSDRUNC
Alcohol and Drugs	Self-Administered
Mental Health	Self-Administered
Opinions About Self	Self-Administered
Experience of Study Participation	Self-Administered stops after PRTPRIVT, then back to Interviewer Administered

PTID

Participant ID
[ ][ ][ ] - [ ][ ][ ][ ][ ] - [ ]
Site Number   Participant Number   Chk

INTERVIEWER: ____________________________ **[OPEN TEXT]**

[Note to Site: Enter the name or initials of the person administering the Enrollment Questionnaire].

SURVEY LANGUAGE

- English (*Show every time)*
- Xhosa (*Only show for Cape Town site)*
- Afrikaans (*Only show for Cape Town site)*
- Sutu *(Only show for Soweto site)*
- Zulu *(Only show for Soweto site)*
- Swahili *(Only show for Kenya site)*
- Luo *(Only show for Kenya site)*
- Chichewa *(Only show for Malawi site)*

Variable Name (VN): **HIVSTATUS**

Interviewer Instructions: Input HIV Status from study HIV Testing (not self-reported by participant)

HIV Status
- HIV-Positive
- HIV-Negative

Demographic Background

Thank you for taking the time to meet with me today. I will now ask you a few questions about yourself. Please remember that you do not have to answer any questions that you do not want to answer and we can stop the interview at any time. If I ask a question that you don't want to answer, just let me know and I will go on to the next question. Do you have any questions before we begin?

I would like to start with some questions about your background.

VN: **DEMEDUC**

1. What is the highest level of education that you completed?
   - None = 0
   - Grade 1/Class 1 = 1
   - Grade 2/Class 2 = 2
   - Grade 3/Class 3 = 3
   - Grade 4/Class 4 = 4
   - Grade 5/Class 5 = 5
   - Grade 6/Class 6 = 6
   - Grade 7/Class 7 = 7
   - Grade 8/Class 8 = 8
   - Grade 9/Form 1 = 9
   - Grade 10/Form 2 = 10
   - Grade 11/Form 3 = 11
   - Grade 12/Form 4 = 12
   - College/University = 13
   - Other = 14
   - No answer=99

VN: **DEMSTUDE**

2. Are you currently a student?

   1 = ☐ Yes
   2 = ☐ No → **GO TO DEMEMPS**
   99=☐ No answer

SHOW IF DEMSTUDE=1

VN: **DEMFTPT**

3. Are you a full-time or part-time student?

   1= ☐ Full time
   2= ☐ Part time
   99=☐ No answer

SHOW ALL

VN: **DEMEMPS**

4. What best describes your current employment status?

   1☐ Employed full-time
   2☐ Employed part-time
   3☐ Self-employed

4☐ Unemployed or between jobs
5☐ On disability
6☐ Other
99☐ No answer

VN: **DEMHH**

5. Which of the following best describes your household?
   1☐ I live by myself
   2☐ I live with a partner or spouse (with or without children)
   3☐ I live with a roommate or roommates (who are not partners)
   4☐ I live with relatives (parents, grandparents, siblings, etc.)
   5☐ I do not have a stable home
   6☐ Other
   99☐ No answer

VN: **DEMMARIT**

6. What is your current marital status?
   1☐ Married/civil union/legal partnership
   2☐ Single/divorced/widowed **→ GO TO DEMKIDS**
   99☐ No answer

SHOW IF DEMMARIT = 1

VN: **DEMSPOUSE**

7. What is the gender of your partner/spouse?
   1☐ Male
   2☐ Female
   3☐ Transgender (male to female)
   4☐ Transgender (female to male)
   5☐ Intersex
   6☐ Other
   99☐ No answer

SHOW ALL

VN: **DEMKIDS**

8. Of how many children are you the biological father?

   ____
   99☐ No answer

VN: **DEMRELIG**

9. What is your current religious affiliation?
   1=Christian
   2=Islam
   3=Baha'i faith
   4=Buddhism
   5=Hinduism
   6=Judaism

7=Animist
8=Traditionalist
9=No religion
99=No answer

VN: **DEMINCO**

10. What is your major source of income?
 1☐ Formal employment
 2☐ Self-employment
 3☐ Family
 4☐ Spouse
 5☐ No monthly income
 6☐ Other
 99☐ No answer

PROMPT: If DEMEMPS= 4, 5 or 6 and DEMINCO=1 or 2, Show soft warning "Participant reported being un-employed in a previous question. Please confirm this response. Click "OK" to continue. Click "CANCEL" to change your response."

VN: **DEMINCMO**

11. On average how much money do you earn or receive in a month? (if none, enter zero)
 (Show if site is Cape Town or Soweto): ____ Rand (*min 0, max 99999; 5 digits*)
 (Show if site is Malawi): ____ Kwacha (*min 0, max 9,999,999; 7 digits*)
 (Show if Site is Kenya): ____ Shilling (*min 0, max 999,999; 6 digits*)
 99☐ No answer

SHOW IF DEMINCMO > 0

VN: **DEMDEPND**

12. Including yourself, how many people depend on this income? (SHOULD AT LEAST BE 1)

 ___________ people including participant (*min 1, max 99*)

 99☐ No answer

VN: **DEMNOMON**

13. In the last 12 months, how often did you run out of money for your basic needs? Would you say…
 1☐ Never
 2☐ Rarely
 3☐ Sometimes
 4☐ Often
 5☐ Almost always
 99☐ No answer

VN: **DEMBORRO**

14. In the last 12 months, how often have you had to borrow money from a friend or relative to survive financially? Would you say…

1☐ Never
2☐ Rarely
3☐ Sometimes
4☐ Often
5☐ Almost always
99☐ No answer

VN: **DEMSECURE**

15. Would you say that financially your future is Very uncertain, Uncertain, Secure, or Very secure?

1☐ Very uncertain
2☐ Uncertain
3☐ Secure
4☐ Very secure
99☐ No answer

VN: **DEMPART**

16. Are you currently in an ongoing, intimate, sexual relationship with a man?

1☐ Yes
2☐ No → **GO TO IDGENDER**
99☐ No answer

SHOW IF DEMPART =1

VN: **DEMPTIME**

17. How long have you been in this relationship?

1☐ Less than a month
2___Months (*min 1, max 11*) **[Allow participant to enter both months and years]**
3___Years (*min 1, max 99*)
99☐ No answer

SHOW IF DEMPART =1

VN: **MPARTLHH**

18. Do you live together in the same household?

1☐ Yes
2☐ No
99☐ No answer

Sexual and Gender Identity, and Social Support

The following questions are about how you see yourself in terms of your gender and your sexuality.

The next question asks about gender. Gender is the social part of being male or female. It relates to your self-identity. When I ask about gender, I am asking about whether you regard yourself to be male, female, transgender female, or if you identify yourself in another way.

VN: **IDGENDER**

19. How do you identify your gender?

1☐ Male
2☐ Female
3☐ Transgender female (male to female)
4☐ Additional category, specify: ________________________
99☐ No answer

VN: **IDMASCUL**

20. If you compare yourself to others, would you say you are generally more like other men or more like women? Would you say you are…

1☐ Like other men
2☐ Like other men as well as like women
3☐ More like women
4☐ The same as women
99☐ No answer

VN: **IDATTRACT**

21. Do you currently feel more sexually attracted to men or to women? Would you say…

1☐ Only to women
2☐ More to women than to men
3☐ To women and men equally
4☐ More to men than to women
5☐ Only to men
99☐ No answer

VN: **IDSEXUAL**

22. Do you identify as gay, bisexual, heterosexual, or transgender, or would you use another word to describe your sexuality?

1☐ Gay → **GO TO IDDECIDE**
2☐ Bisexual → **GO TO IDDECIDE**
3☐ Heterosexual
4☐ Transgender → **GO TO IDDECIDE**
5☐ Other → **GO TO IDDECIDE**
99☐ No answer → **GO TO IDDECIDE**

SHOW IF IDSEXUAL = 3 (HETEROSEXUAL)

VN: **IDEXPLAIN**

23. You told us that you have sex with men and you call yourself heterosexual. Could you explain why you see yourself that way?

____

99☐ No answer

SHOW ALL

To what extent do you agree or disagree with the following statements? For each indicate if you Disagree strongly, Disagree, Agree or Agree strongly.

	Disagree strongly 1	Disagree 2	Agree 3	Agree strongly 4	No answer 99
24. [VN: **IDDECIDE**] I cannot decide whether I am bisexual, homosexual, or heterosexual	☐	☐	☐	☐	☐
25. [VN: **IDISLIKE**] Sometimes I dislike myself for being a man who has sex with other men	☐	☐	☐	☐	☐
26. [VN: **IDWISH**] I wish I were only sexually attracted to women	☐	☐	☐	☐	☐
27. [VN: **IDGLAD**] I am glad to be sexually attracted to other men	☐	☐	☐	☐	☐

For the next questions I will ask how old you were when you first had an experience. If you don't know, please give us your best guess.
How old were you when you first…

	Age in years	N/A	No answer 99
28. [VN: **IDAWARE**]. . . Became aware of your sexual attraction to men?	_______	☐	☐
29. [VN: **IDFIRSTSEX**]. . . Had any kind of sex with a boy or a man?	_______	☐	☐
30. [VN: **IDORGASM**]. . . Had an orgasm when having sex with a man?	_______	☐	☐

SHOW IDRECOGN – IDHIDDEN (#31-34) IF IDSEXUAL ≠ 3 (NOT 3, HETEROSEXUAL)

31. [VN: **IDRECOGN**]. . . Recognized you were gay, bisexual, or transgender?	_______	☐	☐
32. [VN: **IDSAID**].. . . Said to another person you were gay, bisexual, or transgender?	_______	☐	☐
33. [VN: **IDPARENT**].. . . Said to one of your parents that you were gay, bisexual, or transgender?	_______	☐	☐

SHOW IF IDSEXUAL ≠ 3 (NOT 3, HETEROSEXUAL)

VN: **IDHIDDEN**

34. In general, how hard do you try to keep your sexual orientation hidden from your family?

1☐ Try very hard
2☐ Try somewhat hard
3☐ Don't try, but don't talk about it
4☐ I openly talk about it with my family
98☐ Not applicable
99☐ No answer

SHOW IF IDSEXUAL = 3 (HETEROSEXUAL)

VN: **IDHIDDEN2**

35. In general, how hard do you try to keep it hidden from your family that you have sex with men?

1☐ Try very hard
2☐ Try somewhat hard
3☐ Don't try, but don't talk about it
4☐ I openly talk about it with my family
98☐ Not applicable
99☐ No answer

SHOW ALL

Have you, as a result of sexual orientation or practice, in the last 12 months. . .	Yes=1	No=2	No answer =99
36. [VN: **STIGHARA**]. . . Been verbally or physically harassed?	☐	☐	☐
37. [VN: **STIGBEAT**].. . . Been beaten up?	☐	☐	☐
38. [VN: **STIGBLACK**].. . . Been blackmailed?	☐	☐	☐
39. [VN: **STIGLOST**].. . . Lost employment?	☐	☐	☐
40. [VN: **STIGDISC**].. . . Felt legal or police discrimination?	☐	☐	☐

	Yes=1	No=2	No answer =99
41. [VN: **STIGHCAR**] Have you ever felt afraid to go to healthcare services because you worry someone may learn you have sex with men?	☐	☐	☐
42. [VN: **STIGAVOI**] Have you ever avoided going to healthcare services because you worry someone may learn you have sex with men?	☐	☐	☐
43. [VN: **STIGDENY**] Have you ever been denied health services (or someone kept you from receiving health services) because you have sex with men?	☐	☐	☐
44. [VN: **STIGTREA**] Have you ever felt that you were not treated well in a health center because someone knew that you have sex with men?	☐	☐	☐

45. [VN: **STIGACCS**] Have you ever had difficulties in accessing healthcare services because you have sex with men?	☐	☐	☐
46. [VN: **STIGGOSS**] Have you ever heard healthcare providers gossiping (talking) about you because you have sex with men?	☐	☐	☐

How often do the following apply to you? Would you say Never, Rarely, Sometimes, Often or Always?	Never =1	Rarely =2	Sometimes =3	Often =4	Always =5	No answer =99
47. [VN: **SSMONEY**] There is someone you can rely on in case you need money	☐	☐	☐	☐	☐	☐
48. [VN: **SSCLINIC**] There is someone you can rely on to go with you to the doctor, clinic, or hospital	☐	☐	☐	☐	☐	☐
49. [VN: **SSTALK**] There is someone you can rely on to talk to if you have problems	☐	☐	☐	☐	☐	☐
50. [VN: **SSFOOD**] There is someone you can rely on if you need a meal, food, or a place to stay	☐	☐	☐	☐	☐	☐
51. [VN: **SSHURT**] There is someone you can rely on if you get beaten up, attacked, or hurt	☐	☐	☐	☐	☐	☐

To what extent do you agree or disagree with the following statements? Would you say you disagree strongly, disagree, agree, or agree strongly to the following statements…	Disagree strongly =1	Disagree =2	Agree =3	Agree strongly =4	Not Applicable =98	No answer = 99
52. [VN: **SSCONNEC**] I feel strongly connected to other men who have sex with men	☐	☐	☐	☐	☐	☐
53. [VN: **SSCOUNT**] In general, I feel that I can count on members of the gay community if I need help or advice	☐	☐	☐	☐	☐	☐
54. [VN: **SSCOMMUN**] Where I live there is no gay community that I can rely on	☐	☐	☐	☐	☐	☐

Sexual History

I now want to ask you about a completely different topic.

SHOW ALL

VN: **SHCIRCUM**

55. Are you circumcised? By circumcised, I mean that the foreskin of your penis is removed.
    1☐ Yes
    2☐ No → **GO TO SHFORYRS**
    3☐ Don't know → **GO TO SHFORYRS**
    99☐ No answer → **GO TO SHFORYRS**

SHOW IF SHCIRCUM=1

VN: **SHMEDCIR**

56. Was this a medical circumcision, done by a doctor in a clinic or hospital, or was it a traditional circumcision?
    1☐ Medical circumcision
    2☐ Traditional circumcision
    99☐ No answer

SHOW ALL

The following questions are about your sexual history. I first want to ask you about when you were a child or a young teenager.

VN: **SHFORYRS**

57. Before you turned 16, did you ever have any kind of sexual contact with someone who was at least 4 years older?
    1 ☐ Yes
    2 ☐ No → **GO TO SHSEXNO**
    99☐ No answer

SHOW SHSEXONCE – SHABUSE (#58-61) IF SHFORYRS = 1 (YES)

VN: **SHSEXONCE**

58. Did that happen only once or more frequently?
    1☐ Once
    2☐ More often
    99☐ No answer

VN: **SHSEXSEX**

59. Was this with a man or a woman?
    1☐ With a man
    2☐ With a woman
    3☐ With both men and women
    99☐ No answer

VN: **SHSEXBAD**

60. Did this experience (some of these experiences) make you feel uncomfortable, regretful, or bad?

1☐ Yes
2☐ No
99☐ No answer

VN: **SHABUSE**

61. Would you consider this experience (some of these experiences) to be sexual abuse?

1☐ Yes
2☐ No
99☐ No answer

SHOW ALL
VN: **SHSEXNO**

62. How many men have you had sex with in your lifetime? If you don't know exactly, please give us your best estimate.

___________________ number of men (lifetime) (*min 1, max 999*)
99☐ No answer

VN: **SHONGOIN**

63. With how many of these men did you have an ongoing intimate relationship in which you regularly had sex? If you don't know exactly, please give us your best estimate.

___________________men
99☐ No answer

PROMPT: If SHONGOIN>SHSEXNO: "Your response must be less than or equal to total number in your lifetime."

VN: **SHPOSITI**

64. When you have anal sex, do you prefer to be a top or a bottom, or doesn't that matter to you? A "top" means that you are the insertive partner, putting your penis in the anus of a man. A "bottom" means that you are the receptive partner, a man puts his penis in your anus.

1☐Bottom
2☐Top
3☐No preference
4☐Does not prefer anal sex
99☐ No answer

VN: **SHWOMAN**

65. Have you ever had sexual intercourse (anal or vaginal) with a woman?

1☐ Yes
2 ☐ No → **GO TO SHAFTER**
99☐ No answer → **GO TO SHAFTER**

SHOW IF SHWOMAN = 1
VN: **SHFIRSTW**

66. How old were you when you first had sexual intercourse with a woman? (include anal or vaginal)
__________________ years (*MIN 0, MAX 99*)
99☐ No answer

SHOW IF SHWOMAN = 1
VN: **SHWOMNO**

67. How many women have you had sex with in your lifetime?

__________________ women (*MIN 1, MAX 999*)
99☐ No answer

SHOW IF SHWOMAN = 1
VN: **SHWANAL**

68. Have you ever had anal sex with a woman?
   1 ☐ Yes
   2 ☐ No
   99☐ No answer

SHOW ALL
The next questions are about experiences that you might have had after the age of 16.

VN: **SHAFTER**

69. Has a man ever forced you to have sex when you did not want to yourself? This man could have been a stranger, someone you knew, or a regular partner.
   1☐ Yes
   2☐ No → **GO TO SHEXCHNG**
   99☐ No answer → **GO TO SHEXCHNG**

SHOW SHOFTEN – SHFORCYR (#70-73) IF SHAFTER = 1 (YES)

VN: **SHOFTEN**

70. How often would you say you have had such experiences with men?
   1☐ Only once
   2☐ A few times
   3☐ Occasionally
   4☐ Often
   5☐ Very often
   99☐ No answer

VN: **SHWHO**

71. Were these men strangers, somebody you knew, or a regular partner(s)? You may choose more than one.

SHWHO_1☐ Stranger(s)
SHWHO_2☐ Somebody known (including regular partners)
SHWHO_3☐ Regular partner(s)
SHWHO_99☐ No answer

VN: **SHFORCE**

72. Looking back, how severe would you say these forced experiences with men were? Would you say…
   1☐ Not severe at all
   2☐ Somewhat severe
   3☐ Severe
   4☐ Very severe
   99☐ No answer

VN: **SHFORCYR**

73. Have you had any of these forced sex experiences in the past year?
   1☐ Yes
   2☐ No
   99☐ No answer

SHOW ALL
VN: **SHEXCHNG**

74. Sometimes people give or receive something in return for having sex. This can be a variety of things, including food, clothes, a place to sleep, a cell phone, money and a lot of other things. Has a man ever given you something in exchange for sex?
   1 ☐ Yes
   2 ☐ No → **GO TO SHEXGIVE**
   99☐ No answer

SHOW IF SHEXCHNG = 1 (YES)
VN: **SHMANEXC**

75. In the past year, has a man given you anything in exchange for having sex with him?
   1 ☐ Yes
   2 ☐ No → **GO TO SHEXGIVE**
   99☐ No answer

SHOW IF SHMANEXC = 1 (YES)

Which of the following things has a man given you in the past year in exchange for having sex with him? Has he given you…	Yes=1	No=2	No answer = 99
76. [VN: **SHEXFOOD**] Food, clothes and/or cosmetics	☐	☐	☐
77. [VN: **SHEXSLEEP**] A place to sleep	☐	☐	☐
78. [VN: **SHEXCELL**] A cell phone	☐	☐	☐
79. [VN: **SHEXDRUG**] Drugs/alcohol	☐	☐	☐
80. [VN: **SHEXMONE**] Money	☐	☐	☐
81. [VN: **SHEXOTHR**] Other things	☐	☐	☐

SHOW ALL
VN: **SHEXGIVE**

82.Have you ever given a man something in exchange for having sex with you?

1 ☐ Yes
2 ☐ No → **GO TO SEXUAL BEHAVIOR ASSESSMENT (INTRO TEXT ABOVE SPHMANY)**
99☐ No answer

SHOW IF SHEXGIVE= 1
VN: **SHEXGIYR**

83.Have you in the past year given a man something in exchange for having sex with you?

1 ☐ Yes
2 ☐ No → **GO TO SEXUAL BEHAVIOR ASSESSMENT (INTRO TEXT ABOVE SPHMANY)**
99☐ No answer

SHOW IF SHEXGIYR = 1

Which of the following things have you given a man in the past year in exchange for having sex with you?

	Yes=1	No=2	No answer = 99
84.[VN: **SHGFOOD**] Food, clothes and/or cosmetics	☐	☐	☐
85.[VN: **SHGSLEEP**] A place to sleep	☐	☐	☐
86.[VN: **SHGCELL**] A cell phone	☐	☐	☐
87.[VN: **SHGDRUGS**] Drugs/alcohol	☐	☐	☐
88.[VN: **SHGMONY**] Money	☐	☐	☐
89.[VN: **SHGOTHR**] Other things	☐	☐	☐

Sexual Behavior Assessment

Assessment steps
1. Elicit number of sexual partners and nicknames (SPHMANY - SPRP3).
2. Determine sexual frequency and timing (SP1TIMES - SP1RECENT).
3. Assess personal and relational characteristics and sexual behavior partner by partner (SP1GENDER - SP1TSTUDY).
4. If 4 or more sex partners: assess additional sexual risk (SP4UNPSEX - SP5UNPSEX).

This next section will be about persons you have had sex with in the last three months. By “sex” we mean “anal sex” when we talk about male partners; and vaginal or anal sex when we talk about female partners.

In order to do this, we will ask you to provide nicknames. We will then use these nicknames to customize the questions so that they are specific to the persons you have had sex with.

It is important that you choose a nickname that will best help you remember the person. The names you provide are meant to help you only and should not reveal your partner’s full identity. We do not want to know who your partners actually are.

Some examples of nicknames that you might choose are: a person’s first name, a nickname you call the partner by, or the place where you both met. You could also use a description of what a person is to you, such as “lover” or “buddy.”

SHOW ALL
VN: **SPHMANY**

90. How many persons have you had sex with in the past three months? This could be a steady partner, persons that you had sex with only once, that gave you something in return for sex, or that you are no longer in touch with.

_______________ partners
99☐ No answer

IF 0 PARTNERS, SKIP TO INTRODUCTION OF HIV/STI SECTION.

SHOW IF SPHMANY= 1, 2 OR 3

IF SPHMANY = 1: I would now like to ask you some questions about the person you have had sex with in the past 3 months.

IF SPHMANY >1: I would now like to ask you some questions about each of these partners you have had sex with in the past 3 months.

91.
SHOW IF SPHMANY = 1: Could you give me initials or nickname for this person?
SHOW IF SPHMANY > 1: Could you give me initials or a nickname for each of these partners?

SHOW IF SPHMANY = 1
VN: **SP1**
Sexual partner 1: _________ **[OPEN TEXT]**

SHOW IF SPHMANY>1

VN: **SP2**
Sexual Partner 2: ________ **[OPEN TEXT]**

SHOW IF SPHMANY>2
VN: **SP3**
Sexual Partner 3: _________ **[OPEN TEXT]**
If SPHMANY = 1, 2 or 3 partners, skip to "loop of questions to ask each sexual partner" starting with SP1TIMES. If > 3 partners, continue with next set of questions to get nicknames of 3 partners only.

SHOW IF SPHMANY>3 (FOUR OR MORE PARTNERS)
Because it would take too long to discuss them all, I would like to select three of them. There are different types of sexual partners. I am going to ask a few questions to help you select three different types of sexual partners you've had sex with in the past 3 months.

VN: **SPMAIN**

92. How many of the people you had sex with in the last 3 months do you consider to be a main partner? A main partner is someone you have a relationship with, like a lover, boyfriend or girlfriend, or a spouse.
    1 ☐ None → **GO TO SPCASUAL**
    2 ☐ One
    3 ☐ More than one

SHOW IF SPMAIN>1
VN: **SPMP**

93. **IF SPMAIN = 3:** Think about the most important one and please give me initials or a nickname for this person.
IF SPMAIN = 2: Please give me initials or a nickname for this person.

__________ **[OPEN TEXT]**

VN: **SPCASUAL**
94. (Not including anyone we may have already discussed), how many of the people you had sex with in the last 3 months do you consider to be a casual partner? A casual partner is someone with whom you just have sex once or twice, or that you hook up with from time to time to have sex.
    1☐None→ **GO TO SPEXCHAN**
    2☐One
    3☐More than one

SHOW IF SPCASUAL>1
VN: **SPCP**

95. **IF SPCASUAL = 3:** Think about the <u>most recent</u> one and please give me initials or a nickname for this person.
IF SPCASUAL = 2: Please give me initials or a nickname for this person.

_________ **[OPEN TEXT]**

VN: **SPEXCHAN**
96. (Not including anyone we may have already discussed), are any of these persons an exchange partner? An exchange partner is someone you have sex with in exchange for food, money, shelter or drugs.
    1☐None→ **GO TO SPWOMAN**
    2☐One
    3☐More than one

SHOW IF SPEXCHAN> 1

VN: **SPEP**

97.**IF SPEXCHAN = 3:** Think about the most recent one and please give me initials or a nickname for this person.
IF SPEXCHAN = 2: Please give me initials or a nickname for this person.

___________ **[OPEN TEXT]**

SHOW IF 3 PARTNERS HAVE NOT ALREADY BEEN SELECTED (IF ANY 1 OF SPMP, SPCP OR SPEP = 1 (NONE))

VN: **SPWOMAN**

98.Not including anyone we may have already discussed, are any of the people you've had sex with in the past 3 months a (biological) woman?

1 ☐ None
2 ☐ One
3 ☐More than One

SHOW IF SPWOMAN >1
VN: **SPWP**

99. **IF SPWOMAN = 3:** Think about the most recent one not already chosen and please give me initials or a nickname for this person.
IF SPWOMAN = 2: Please give me initials or a nickname for this person.

____________ **[OPEN TEXT]**

SHOW IF 3 PARTNERS HAVE NOT ALREADY BEEN SELECTED (IF ANY 2 OF SPMAIN, SPCASUAL, SPEXCHAN OR SPWOMAN = 1 (NONE))

VN: **SPRP1**

100. Not including anyone we may have already discussed, think about your next most recent sexual partner in the past three months.

Please give me initials or a nickname for this person. ______ **[OPEN TEXT]**

SHOW IF 3 PARTNERS STILL HAVE NOT BEEN SELECTED (IF ANY 3 OF SPMAIN, SPCASUAL, SPEXCHAN, SPWOMAN OR SPRP1 = 1 (NONE))

VN: **SPRP2**

101. Not including anyone we may have already discussed, think about your next most recent sexual partner in the past three months.

Please give me initials or a nickname for this person. ______ **[OPEN TEXT]**

SHOW IF 3 PARTNERS STILL HAVE NOT BEEN SELECTED (IF SPMAIN, SPCASUAL, SPEXCHAN AND SPWOMAN ALL = 1 (NONE) AND SPRP1 AND SPRP2 HAVE ANSWERS.

VN: **SPRP3**

102. Not including anyone we may have already discussed, think about your next most recent sexual partner in the past three months.

Please give me initials or a nickname for this person. ______ **[OPEN TEXT]**

LOOP OF QUESTIONS TO ASK FOR EACH SEXUAL PARTNER

VN: SP1TIMES

103. In the past 3 months, have you had sex with [XXX] once or more than once?
1 ☐ One time
2 ☐ More than once

SHOW IF SP1TIMES = 1
VN: **SP1DATE**

104. In the past 3 months, when did you have sex with [XXX]? If you don't know exactly please give your best guess.
_____MM/YYYY
2 ☐Unknown
99☐ No answer

PROMPT: If MM/YYYY > today's date or not within 3 months from today's date: "Your response must be before today and within the last 3 months. Please correct your response. "

SHOW IF SP1TIMES = 2
VN: **SP1FIRST**

105. When was the first time you had sex with [XXX]? Please provide the month and year. If you don't know exactly, please give your best guess. (*You may enter 99 for the month if the participant does not want to guess.*)
_____MM/YYYY **(Allow 99 for MM)**
2 ☐Unknown
99☐ No answer

PROMPT: If MM/YYYY is > today's date: "Your response cannot be a date in the future. Please correct your response. "

SHOW IF SP1TIMES = 2
VN: **SP1RECENT**

106. When was the most recent time you had sex with [XXX]?
_____MM/YYYY
2 ☐Unknown
99☐ No answer

PROMPT: If MM/YYYY > today's date or not within 3 months from today's date: "Your response must be before today and within the last 3 months. Please correct your response."

PROMPT: IF SP1RECENT IS < SP1FIRST: "The most recent time you had sex with your partner has to be on or after the date you first had sex with that partner. Please correct your response."

SHOW ALL
VN: **SP1GENDER**

107. Is [XXX] male, female, or transgender?

1☐ Male
2☐ Female
3☐ Transgender: male to female
4☐ Transgender: female to male
5☐ Intersex
99☐ No answer

VN: **SP1WHERE**

108. Where did you first meet [XXX]?

1☐ Through friends
2☐ In a bar/club/dancing
3☐ School or work
4☐ At church
5☐ At social event
6☐ Via the internet/social media
7☐ Cruising area (park/public ground/bushes, public toilet, railway/bus station)
8☐ On the street
9☐ Other
99☐ No answer

VN: **SP1FMEET**

109. Do you remember the month and year when you met [XXX] for the first time? If not, give your best guess.

____MM/YYYY **(Allow 99 for MM)**
99☐ No answer

PROMPT: If SP1FMEET MM/YYYY is after the current MM/YYYY: "Your response cannot be a date in the future. Please correct your response."

VN: **SP1AGE**

110. What is [XXX]'s current age (if you are unsure of the exact age, choose an age that you think is close)?

1 ☐ ______________years → **GO TO SP1RACE**
2 ☐ Don't know
99☐ No answer → **GO TO SP1RACE**

SHOW IF SP1AGE = 2
VN: **SP1OLDER**

111. Compared to you, is [XXX] much older, somewhat older, about the same age, or somewhat or much younger?

1☐ Much older
2☐ Somewhat older
3☐ (More or less) same age
4☐ Somewhat younger
5☐ Much younger
99☐ No answer

VN: **SP1RACE**

112. What is the race or ethnicity of [XXX]?

Show If Site is Soweto or Cape Town:
1=Black
2=White
3= Indian/Asian
4= Coloured
5=Other
99=No answer

Show If Site is Malawi:
2= White
6=Chewa
7= Ngoni
8= Yao
9= Tumbuka
5= Other
99=No answer

Show if Site is Kenya
10=Luo,
11= Kisii
12=Luhya
13=Kalenjin
2=White
3= Indian/Asian
5=Other
99=No answer

SHOW IF SP1GENDER = 1
VN: **SP1ATTRACT**

113. As far as you know, is [XXX] sexually attracted to men or to women?
1☐ Only to women
2☐ More to women than to men
3☐ To women and men equally
4☐ More to men than to women
5☐ Only to men
6☐ Don't know
99☐ No answer

SHOW IF SP1GENDER = 1
VN: **SP1FARLIVE**

114. How far do you live from [XXX]?
1☐ Same household
2☐ Same area of town
3☐ Another area of town
4☐ Outside of the city
5☐ Outside of the country
6☐ Don't know
7☐ Person listed is homeless and is not living anywhere regularly
99☐ No answer

SHOW IF SP1GENDER = 1
VN: **SP1MONEY**

115. Who would you say has generally more money to spend, [XXX] or you?
1☐ Partner
2☐ Participant self

3☐ The same
4 ☐Don't know
99☐ No answer

SHOW IF SP1GENDER = 1
VN: **SP1MASCU**

116. Would you say that [XXX] is more or less masculine than you?
1☐ Partner more masculine
2☐ Partner less masculine
3☐ The same
99☐ No answer

SHOW ALL
VN: **SP1KNOWN**

117. How well did you know [XXX] prior to having sex with him/her for the first time?
1☐ Not at all (just met)
2☐ Somewhat
3☐ Fairly well
4☐ Very well
99☐ No answer

SHOW IF SP1TIMES=2
VN: **SP1CLOSE**

118. How do you feel about your relationship with [XXX]? Would you say your relationship is:
1☐ Very close
2☐ Somewhat close
3☐ Not at all close
99☐ No answer

SHOW ALL
VN: **SP1SHARE**

119. **IF SP1TIMES = 1:** Did [XXX] share his/her HIV status with you <u>before</u> you had sex?
IF SP1TIMES = 2: Did [XXX] share his/her HIV status with you <u>before</u> you first had sex?
1☐ Yes
2☐ No → **GO TO SP1STATBFS**
3☐ Don't know → **GO TO SP1STATBFS**
99☐ No answer

SHOW IF SP1SHARE =1 (YES)
VN: **SP1STATBS**

120. What was [XXX]'s status at that time?
1☐ HIV-negative
2☐ HIV-positive
3☐ Don't know
99☐ No answer

SHOW ALL
VN: **SP1STATBFS**

121. **IF SP1TIMES = 1:** Did you share your HIV status with him/her <u>before</u> you had sex?
IF SP1TIMES = 2: Did you share your HIV status with him/her <u>before</u> you first had sex?
1☐ Yes
2☐ No

3☐ Don’t know
99☐ No answer

SHOW IF SP1TIMES =2 AND SP1SHARE = 2 OR 3
VN: **SP1SHARAS**

122. Did you and [XXX] share both of your HIV statuses any time after you first had sex?
1☐ Yes
2☐ No → **GO TO SP1PLACE**
3☐ Don’t know → **GO TO SP1PLACE**
99☐ No answer

SHOW IF SP1TIMES =2 & SP1SHARAS= 1 (SP1SHARE = 2 OR 3)
VN: **SP1STATAS**

123. What was [XXX]’s status at that time?
1☐ HIV-negative
2☐ HIV-positive
3☐ Don’t know
99☐ No answer

SHOW ALL
VN: **SP1PLACE**

124. Where did you have sex with [XXX]? You can select more than one response.
(SP1PLACE_1)☐ At participant’s place
(SP1PLACE_2)☐ At partner’s place
(SP1PLACE_3) ☐ At the place we live together
(SP1PLACE_4)☐ Elsewhere
(SP1PLACE_99) ☐ No answer

SPECIFIC QUESTIONS (PART OF LOOP OF QUESTIONS) FOR A MALE SEX PARTNER

SHOW IF SP1GENDER = 1, 3, OR 5 (MALE)

VN: **SP1ANAL**

125. Did you have receptive anal sex with [XXX], meaning you were the “bottom”, in the past 3 months?
1☐ Yes
2☐ No → **GO TO SP1INSERT**
99☐ No answer → **GO TO SP1INSERT**

TOP OF PAGE: In the past 3 months…

SHOW IF SP1ANAL=1 (YES) AND SP1TIMES=1

VN: **SP1ASRASC**

126. Did [XXX] use a condom when you had receptive anal sex with him?

1☐ He used condom
2☐ He did not use condom → **GO TO SP1INSERT**
3☐ Cannot remember → **GO TO SP1INSERT**
99☐ No answer → **GO TO SP1INSERT**

TOP OF PAGE: In the past 3 months…

SHOW IF SP1ASRASC =1
VN: **SP1RASCT**

127. Did [XXX] wear the condom all the time or part of the time while he was inside you?
1☐ All the time
2☐ Part of the time
3☐ Cannot remember
99☐ No answer

SHOW IF SP1ANAL=1 (YES) & SP1TIMES=2
VN: **SP1ANALNO**

128. How many times have you had receptive anal sex with [XXX] in the past 3 months? If you don't know exactly, give us your best guess.

________ times
99☐ No answer

PROMPT: If SP1ANAL=1, SP1ANALNO must be > 0: "Your response must be greater than 0. Please correct your response."

SHOW IF SP1ANAL=1 & SP1TIMES=2
VN: **SP1UNPROT**

129. Most men do not use condoms all the time they have anal sex. Of these **{R: SP1ANALNO}** times that you had receptive anal sex with [XXX], how many were unprotected, that means that no condom was used? If you don't know exactly, give us your best guess.

________ times (*MAX = SP1ANALNO*)
99☐ No answer

PROMPT: If number of times entered is > value of SP1ANALNO: "Your response must be less than or equal to the total number of times you had receptive anal sex with this partner in the last 3 months."

SHOW IF SP1TIMES=2 & SP1UNPROT < SP1ANALNO
VN: **SP1ANCON**

130. Sometimes men only put on a condom after they have already had their penis inside the other person's anus. In the past 3 months, how many times did [XXX] only use condoms part of the time while he was inside you? If you don't know exactly, give us your best guess.

________ times
99☐ No answer

SHOW IF SP1TIMES = 1 or 2
VN: **SP1INSERT**

131. Did you have insertive anal sex with [XXX], meaning you were the "top", in the past 3 months?
1☐ Yes
2☐ No → **GO TO LOGIC BEFORE SP1GOTCON, IF APPLICABLE; OTHERWISE GO TO SP1AODOTH**
99☐ No answer

TOP OF PAGE (next two items): In the past 3 months…

SHOW IF SP1INSERT=1 (YES) & SP1TIMES = 1
VN: **SP1ASIASC**

132. Did you use a condom when you had insertive anal sex with him?
1☐ I used condom
2☐ I did not use condom
3☐ Cannot remember
99☐ No answer

SHOW IF SP1ASIASC=1 & SP1TIMES =1
VN: **SP1IASCT**

133. Did you wear the condom all the time or part of the time while you were inside of [XXX]?
1☐ All the time
2☐ Part of the time
3☐ Cannot remember
99☐ No answer

SHOW IF SP1INSERT=1 & SP1TIMES = 2
VN: **SP1INTIMES**

134. How many times have you had insertive anal sex with [XXX] in the past 3 months? If you don't know exactly, give us your best guess.

________ times
99☐ No answer

PROMPT: If SP1INSERT=1, SP1INTIMES must be > 0: "Your response must be greater than 0. Please correct your response."

SHOW IF SP1INSERT=1 & SP1TIMES = 2
VN: **SP1INPROT**

135. Most men do not use condoms all the time they have anal sex. Of these **{R: SP1INTIMES}** times that you had insertive anal sex with [XXX], how many were unprotected, that means that no condom was used? If you don't know exactly, give us your best guess.

________ times (*max = value of SP1INTIMES*)
99☐ No answer

PROMPT: If number of times entered is > SP1INTIMES: "Your response must be less than or equal to the total number of times you had insertive anal sex with this partner in the last 3 months."

SHOW IF SP1TIMES = 2 & (SP1INPROT< SP1INTIMES) (IF CONDOMS HAD BEEN USED IN INSERTIVE SEX)
VN: **SP1INCON**

136. Sometimes men only put on a condom after they have already been with their penis inside the other person's anus. In the past 3 months, how many times did you only use condoms part of the time while you were inside [XXX]? If you don't know exactly, give us your best guess.

_________ times
99☐ No answer

NOTE: After these sex questions for a male sex partner, continue with logic above SP1GOTCON.

SPECIFIC QUESTIONS (PART OF LOOP OF QUESTIONS) FOR A FEMALE SEX PARTNER

SHOW IF SP1GENDER=2, 4 (FEMALE)

VN: **SP1VAGIN**

137. Did you have vaginal intercourse with [XXX] in the past 3 months?
1☐ Yes
2☐ No → **GO TO SP1FEMAN**
99☐ No answer

SHOW IF SP1VAGIN = 1 & SP1TIMES = 1

TOP OF PAGE: In the past 3 months…

VN: **SP1VAGCON**

138. Did you use a condom when you had vaginal intercourse with [XXX]?
1☐ I used condom
2☐ I did not use condom
3☐ Cannot remember
99☐ No answer

SHOW IF SP1VAGCON = 1 & SPTIMES=1
VN: **SP1VCTIM**

139. Did you wear the condom all the time or part of the time when you were inside [XXX]?
1☐ All the time
2☐ Part of the time
3☐ Cannot remember
99☐ No answer

SHOW IF SP1VAGIN = 1 & SP1TIMES=2
VN: **SP1VTIMES**

140. How many times have you had vaginal intercourse with [XXX] in the past 3 months?

________ times
99☐ No answer

PROMPT: If SP1VAGIN=1, SP1VTIMES must be > 0: "Your response must be greater than 0. Please correct your response."

SHOW IF SP1VAGIN = 1 & SP1TIMES=2
VN: **SP1VPROT**

141. Of these **{R: SP1VTIMES}** times that you had vaginal intercourse with [XXX], how many were unprotected, that means that no condom was used?

________ times (*MAX = SP1VTIMES*)
99☐ No answer

PROMPT: If number of times entered is > SP1VTIMES: "This response must be less than or equal to the total number of times you had vaginal intercourse with this partner in the last 3 months."

SHOW IF SP1VAGIN=1 & SP1TIMES=2 AND (SP1VTIMES> SP1VPROT) (IF CONDOMS HAD BEEN USED IN VAGINAL SEX)

VN: **SP1VAGCPT**

142. Sometimes men only put on a condom after they have already been with their penis inside the woman's vagina. In the past 3 months, how many times did you only use condoms part of the time while you were inside [XXX]?

________ times
99☐ No answer

SHOW IF SP1TIMES = 1 OR 2
VN: **SP1FEMAN**

143. Did you have anal sex with [XXX] in the past 3 months?
1☐ Yes
2☐ No → **GO TO LOGIC BEFORE SP1GOTCON, IF APPLICABLE; OTHERWISE GO TO SP1AODOTH**
99☐ No answer

SHOW IF SP1FEMAN = 1 & SP1TIMES=1
VN: **SP1FEMAC**

144. Did you use a condom when you had anal sex with [XXX]?
1☐ I used condom
2☐ I did not use condom
3☐ Cannot remember
99☐ No answer

SHOW IF SP1FEMAC = 1 & SP1TIMES=1
VN: **SP1FASCT**

145. Did you wear the condom all the time or part of the time while you were inside of [XXX]?

1☐ All the time
2☐ Part of the time
3☐ Cannot remember
99☐ No answer

SHOW IF SP1FEMAN=1 & SP1TIMES=2
VN: **SP1FATIMES**

146. How many times have you had anal sex with [XXX] in the past 3 months?

________ times
99☐ No answer

PROMPT: If SP1FEMAN=1, SP1FATIMES must be > 0: "Your response must be greater than 0. Please correct your response"

SHOW IF SP1FEMAN=1 & SP1TIMES=2
VN: **SP1FAPROT**

147. Most men do not use condoms all the time they have sex with a woman. Of these **{R: SP1FATIMES}** times that you had anal sex with [XXX], how many were unprotected, that means that no condom was used?

________ times (*MAX = SP1FATIMES*)
99☐ No answer

PROMPT: If number of times entered is > SP1FATIMES: "This response must be less than or equal to the total number of times you had anal sex with this partner in the last 3 months."

SHOW IF SP1FEMAN = 1 AND SP1TIMES=2 AND (SP1FATIMES> SP1FAPROT) (IF CONDOMS HAD BEEN USED IN ANAL SEX WITH FEMALE PARTNER)
VN: **SP1FACON**

148. Sometimes men only put on a condom after they have already been with their penis inside the woman's anus. In the past 3 months, how many times did you only use condoms part of the time while you were inside [XXX]?

________ times
99☐ No answer

NOTE: After these sex questions for a female sex partner, continue with logic above SP1GOTCON.

SHOW TO MALE OR FEMALE PARTNERS IF CONDOMS HAVE BEEN USED OR PARTICIPANT IS UNSURE IF CONDOMS WERE USED, DEFINED AS:

FOR SP1TIMES = 2: CONDOMS WERE USED IF SP1UNPROT < SP1ANALNO, OR SP1INPROT < SP1INTIMES OR SP1VPROT < SP1VTIMES OR SP1FAPROT < SP1FATIMES

FOR SP1TIMES = 1: CONDOMS WERE USED IF SP1ASRASC = 1 or SP1ASIASC=1 or SP1VAGCON =1 or SP1FEMAC = 1

Now I'm going to ask a few questions about your use of condoms in the past 3 months.

VN: **SP1GOTCON**

149. **IF SP1TIMES = 1:** Who provided the condom?

IF SP1TIME = 2: Who took care that condoms were available?

1☐ Participant
2☐ Sexual Partner ([XXX])
3☐ Both
99☐ No answer

VN: **SP1WANTCON**

150. Were condoms used because <u>[XXX]</u> or because <u>you</u> wanted to use them?

1☐ Participant wanted to use them
2☐ Partner wanted to use them
3☐ Both wanted to use them
99☐ No answer

TOP OF PAGE (next 2 items): In the past 3 months…

SHOW IF SP1TIMES=2
VN: **SP1CSLPT**

151. When you and [XXX] used condoms, how many times did the condom slip off?

_________ times
99☐ No answer

SHOW IF SP1TIMES=2
VN: **SP1CBRKT**

152. When you and [XXX] used condoms, how many times did the condom break?

_________ times
99☐ No answer

TOP OF PAGE: In the past 3 months…

SHOW IF SPTIMES=1
VN: **SP1CSLIP**

153. When you and [XXX] used condoms, did the condom slip off?

1☐ Condom slipped of
2☐ Condom did not slip off
3☐ Don't remember
99☐ No answer

SHOW IF SPTIMES=1
VN: **SP1CBREK**

154. When you and [XXX] used condoms, did the condom break?

1☐ Condom did break
2☐ Condom did not break
3☐ Don't remember
99☐ No answer

SHOW TO MALE OR FEMALE PARTNERS IF CONDOMS HAD <u>NOT</u> BEEN USED OR PARTICIPANT WAS UNSURE, DEFINED AS:

FOR SP1TIMES = 2: CONDOMS WERE NOT USED IF SP1UNPROT = SP1ANALNO (RECEPTIVE ANAL SEX MALE PARTNER) OR SP1INPROT = SP1INTIMES (INSERTIVE ANAL SEX MALE PARTNER) OR SP1VPROT = SP1VTIMES (VAGINAL SEX FEMALE PARTNER) OR SP1FAPROT = SP1FATIMES (ANAL SEX FEMALE PARTNER)

VN: **SP1NOTUSE**

155. What was the main reason why condoms were not (always) used when you had sex with [XXX]?
1☐ Condom wasn't available
2☐ Condom was available, but I didn't want to use one
3☐ Condom was available, but [XXX] didn't want to use one
4☐ Both HIV negative
5☐ Other reason
99☐ No answer

FOR ALL PARTNERS (MALE OR FEMALE) SHOW IF SP1ANAL=1 OR SP1INSERT=1 OR SP1FEMAN=1 (IF PARTICIPANT HAD ANAL SEX WITH PARTNER)

TOP OF PAGE: In the past 3 months…

SHOW IF SP1TIMES =1
VN: **SP1LUBE1**

156. Was lubricant used when [XXX] and you had anal sex?
1☐ Yes
2☐ No
3☐ Don't remember
99☐ No answer

SHOW IF SP1TIMES =2
VN: **SP1LUBE2**

157. How often was lubricant used when [XXX] and you had anal sex? Would you say….?
1☐ Never
2☐ Rarely
3☐ Sometimes
4☐ Most of the times
5☐ Always
99☐ No answer

INSERT PAGE BREAK

AT TOP OF PAGE: In the past 3 months…

SHOW IF SP1LUBE1 =1 OR SP1LUBE2>1 (SP1TIMES CAN = 1 OR 2). DO NOT SHOW IF SP1LUBE1 OR SP1LUBE2 = 99.
VN: **SP1LUBTYPE**

158. What type of lubricant did you use with [XXX]? *(Mark all that apply)*

SP1LUBTYPE_1☐ Vaseline
SP1LUBTYPE_2☐ Commercially or clinic-sourced product (e.g., KY Jelly)
SP1LUBTYPE_3☐ Body lotion or baby oil
SP1LUBTYPE_4☐ Vegetable or food oil
SP1LUBTYPE_5☐ Butter or margarine
SP1LUBTYPE_6☐ Soap
SP1LUBTYPE_7☐ Grease
SP1LUBTYPE_8☐ Saliva or water
SP1LUBTYPE_9☐ Other
SP1LUBTYPE_99☐ No answer

INSERT PAGE BREAK

**SHOW IF SP1TIMES = 1 OR 2**

AT TOP OF PAGE: In the past 3 months…

VN: **SP1AODOTH**

159. **IF SP1TIMES = 1:** Was [XXX] under the influence of alcohol or drugs when you and [XXX] had sex?
IF SP1TIMES = 2: How often was [XXX] under the influence of alcohol or drugs when you and [XXX] had sex?

1☐ Never
2☐ Rarely
3☐ Sometimes
4☐ Most of the time
5☐ Always
6☐ No
7☐ Yes
99☐ No answer

SHOW No (6), Yes (7) and No answer (99) IF (SP1TIMES) = 1
SHOW Never (1), "Rarely (2), Sometimes (3), Most of the time (4), Always (5) and No answer (99) IF (SP1TIMES) = 2

VN: **SP1AODSELF**

160. **IF SP1TIMES = 1:** Were you under the influence of alcohol or drugs when you and [XXX] had sex?
IF SP1TIMES = 2: How often were you under the influence of alcohol or drugs when you and [XXX] had sex?

1☐ Never
2☐ Rarely
3☐ Sometimes
4☐ Most of the time
5☐ Always
6☐ No
7☐ Yes
99☐ No answer

SHOW No (6), Yes (7) and No answer (99) IF (SP1TIMES) = 1
SHOW Never (1), "Rarely (2), Sometimes (3), Most of the time (4), Always (5) and No answer (99) IF (SP1TIMES) = 2

TOP OF PAGE: In the past 3 months…

VN: **SP1PAYYOU**

161. **IF SP1TIMES = 1:** Did [XXX] pay you for having sex with him/her?
IF SP1TIMES = 2: Did [XXX] ever pay you for having sex with him/her?

1☐ Yes
2☐ No
99☐ No answer

VN: **SP1GETELSE**

162. **IF SP1TIMES = 1:** Did you get anything else from [XXX] for having sex with him/her, such as food, a place to sleep, or clothing?
IF SP1TIMES = 2: Did you ever get anything else from [XXX] for having sex with him/her, such as food, a place to sleep, or clothing?

1☐ Yes
2☐ No
99☐ No answer

TOP OF PAGE: In the past 3 months

VN: **SP1PAYTHEM**

163. **IF SP1TIMES = 1:** Did you pay [XXX] for having sex with you?
IF SP1TIMES = 2: Did you ever pay [XXX] for having sex with you?

1☐ Yes
2☐ No
99☐ No answer

VN: **SP1GIVELSE**

164. **IF SP1TIMES = 1:** Did you give [XXX] anything for having sex with you, such as food, a place to sleep, or clothing?
IF SP1TIMES = 2: Did you ever give [XXX] anything for having sex with you, such as food, a place to sleep, or clothing?

1☐ Yes
2☐ No
99☐ No answer

VN: **SP1FORCED**

165. **IF SP1TIMES = 1:** Did you feel that [XXX] forced you to have sex with him/her when you did not want to yourself?
IF SP1TIMES = 2: In the last 3 months, did you ever feel that [XXX] forced you to have sex with him/her when you did not want to yourself?

1☐ Yes
2☐ No **→ GO TO LOGIC BEFORE SP1OFTYFT**
99☐ No answer

SHOW IF SP1FORCED = 1 & SP1TIMES=2
VN: **SP1OFTYFF**

166. How often would you say you felt forced by [XXX] to have sex?

1☐ Only once
2☐ A few times
3☐ Regularly
4☐ Often

5☐ Very often
99☐ No answer

SHOW IF SP1TIMES = 1
VN: **SP1FORCY**

167. Did you force [XXX] to have sex with you?
1☐ Yes
2☐ No
99☐ No answer

SHOW IF SP1TIMES=2
VN: **SP1OFTYFT**

168. How often would you say you have forced [XXX] to have sex with you in the last 3 months?
1☐ Never
2☐ Only once
3☐ A few times
4☐ Regularly
5☐ Often
6☐ Very often
99☐ No answer

**SHOW SP1SWW – SP1TSTUDY IF SP1GENDER=1 (IF PARTNER IS MALE)**

VN: **SP1SWW**

169. As far as you know, does [XXX] also have sex with women?
1☐ Yes
2☐ No
3☐ Don't know
99☐ No answer

SHOW IF SP1TIMES=2
VN: **SP1SWO**

170. In the last 3 months, during the time you were sexually involved with [XXX], did [XXX] have sex with anyone else?
1☐ Yes
2☐ No
3☐ Don't know
99☐ No answer

SHOW IF SP1TIMES=1 OR 2
VN: **SP1AGAIN**

171. Do you think you will have sex again with [XXX]?
1☐ Yes
2☐ No
3☐ Don't know
99☐ No answer

SHOW IF SP1TIMES=2
VN: **SP1YSTUDY**

172. Have you ever talked with [XXX] about your participation in this research study?
1☐ Yes
2☐ No
3☐ Don't know
99☐ No answer

SHOW IF SP1TIMES=1 OR 2
VN: **SP1TSTUDY**

173. As far as you know, does [XXX] participate in this research study?
1☐ Yes
2☐ No
3☐Don't know
99☐ No answer

GO BACK TO START OF SEXUAL PARTNER SECTION (SP1TIMES IS THE FIRST QUESTION) IF MORE PARTNERS HAVE TO BE DISCUSSED. OTHERWISE:

IF SP#GENDER=2 (FEMALE) THEN GO TO SPFEMIW

IF SP#GENDER ≠ 2 (IS NOT FEMALE) AND NO MORE PARTNERS AND PARTICIPANT HAD 3 OR LESS PARTNERS CONTINUE WITH HIV/STI SECTION

IF 3 PARTNERS HAVE BEEN DISCUSSED AND PARTICIPANT HAD 4 PARTNERS CONTINUE WITH SP4UNPSEX

IF 3 PARTNERS HAVE BEEN DISCUSSED AND PARTICIPANT HAD MORE THAN 4 PARTNERS CONTINUE WITH SP5UNPSEX

ADDITIONAL SEXUAL RISK QUESTIONS IF SPHMANY = 4 ONLY

SHOW IF SPHMANY = 4
VN: **SP4UNPSEX**

174. You said that you had had sex with one other person in the last 3 months. Did you ever have unprotected anal or vaginal sex with this person in this period?
1☐ Yes
2☐ No, never
3☐ Can't remember
99☐ No answer

SHOW IF SPHMANY = 4
VN: **SP4YSTUDY**

175. Have you ever talked with this person about your participation in this research study?
1☐ Yes
2☐ No
3☐ Don't know

99☐ No answer

ADDITIONAL SEXUAL RISK QUESTIONS IF SPMANY > 4

SHOW IF SPHMANY > 4
VN: **SP5UNPSEX**

176. You said that you had had sex with several other persons in the last 3 months. Did you ever have unprotected anal or vaginal sex with any of these persons in this period?

1☐ Yes
2☐ No, never
3☐ Can't remember
99☐ No answer

SHOW IF SPHMANY > 4
VN: **SP5YSTUDY**

177. Have you ever talked with these persons about your participation in this research study?

1☐ Yes
2☐ No
3☐ Don't know
99☐ No answer

SHOW IF SP#GENDER = 2 (FEMALE) (IF PERSON HAD SEX WITH A WOMAN)

VN: **SPFEMIW**

178. You mentioned that you had sex with one or more women in the past 3 months. Do you think that if we want to interview her/them, this woman/these women would be willing to come to our study offices for an interview?

1☐ Definitively
2☐ Probably
3☐ Probably not
4☐ Definitively not
5☐ Don't know
99☐ No answer

HIV/STI-Related Factors

The next questions are about HIV and sexually transmitted infections. Most people prefer to answer them privately, by reading and recording their responses on their own. Some people prefer that I continue to read the questions out loud and record their responses for them. Which would you prefer?

INTERVIEWER RADIO BUTTON

- **SELF-ADMINISTERED**
- **INTERVIEWER ADMINISTERED**

[SHOW IF SELF-ADMINISTERED]: *(Interviewer to read out loud FIRST):* As you read and respond to the questions in this section, I will stay in the room to help or to answer any questions you might have. You can skip any question you choose but please remember your responses are very important and will remain confidential.

[SHOW IF INTERVIEWER ADMINISTERED]: Ok, let's continue. Like I said, the following questions are about HIV, AIDS, other sexually transmitted infections and how they can be prevented.

**Have you ever heard of:**	Yes = 1	No = 2	No answer = 99
179. [VN: **HIVHERPES**] Genital herpes	☐	☐	☐
180. [VN: **HIVGONOR**] Gonorrhea	☐	☐	☐
181. [VN: **HIVWARTS**]Genital warts	☐	☐	☐
182. [VN: **HIVCHLAM**]Chlamydia	☐	☐	☐

[SHOW IF SELF-ADMINISTERED]: Please indicate whether each of the following statements are true, false or you do not know by checking the box next to the statement.

[SHOW IF INTERVIEWER ADMINISTERED]: Please indicate whether you think the following statements are true or false. You can also say that you don't know the answer.

	True = 1	False = 2	Do not know = 3	No answer = 99
183. [VN: **HIVHVIRU**] Once a person has caught genital herpes they will always have the herpes virus	☐	☐	☐	☐
184. [VN: **HIVORAL**] Gonorrhea can be transmitted through oral sex	☐	☐	☐	☐
185. [VN: **HIVSPREAD**] Genital warts can only be spread by intercourse	☐	☐	☐	☐
186. [VN: **HIVCWOME**] Chlamydia affects only women	☐	☐	☐	☐
187. [VN: **HIVAIDS**] HIV is a virus that weakens the immune system, leading to other infections, cancers, and AIDS	☐	☐	☐	☐
188. [VN: **HIVCREDU**] Using condoms when you have sex can reduce the chance of getting HIV	☐	☐	☐	☐
189. [VN: **HIVCMORE**] It is safe to use the same condom more than once	☐	☐	☐	☐
190. [VN: **HIVVACCI**] There is a vaccine that can stop people from getting HIV	☐	☐	☐	☐
191. [VN: **HIVORALS**] Oral sex is just as risky as anal intercourse for transmitting HIV	☐	☐	☐	☐

192. [VN: **HIVSSCON**] It is safe to have sex without a condom if it is with your regular partner	☐	☐	☐	☐
193. [VN: **HIVOILUB**] When you use condoms it is okay to also use oil-based lubricants	☐	☐	☐	☐
194. [VN: **HIVPOUT**] ‘‘Pulling out’’ before the male ejaculates prevents transmission of HIV	☐	☐	☐	☐
195. [VN: **HIVWASH**] As long as both partners wash themselves after sex, it is not necessary to use condoms	☐	☐	☐	☐
196. [VN: **HIVCURE**] There is a cure for AIDS	☐	☐	☐	☐
197. [VN: **HIVSEEIT**] If someone has HIV you can see that straightaway	☐	☐	☐	☐
198. [VN: **HIVTANAL**] HIV can be transmitted through anal sex	☐	☐	☐	☐

VN: **HIVANALR**

199. If two men have anal sex and do not use a condom, who is more at risk for HIV infection: the top, the bottom, or is the risk the same for both?

1☐ Top more at risk
2☐ Bottom more at risk
3☐ Risk same for both
4☐ Don't know
99☐ No answer

[SHOW IF SELF-ADMINISTERED]: Choose the response that best represents to what extent you agree or disagree with the following statements.

[SHOW IF INTERVIEWER-ADMINISTERED]: To what extent you agree or disagree with the following statements? Do you Disagree strongly, Disagree, Agree or Agree strongly…

	Disagree strongly 1	Disagree 2	Agree 3	Agree strongly 4	No answer 99
200. [VN: **HIVSTIM**]The use of condoms can make sex more stimulating	☐	☐	☐	☐	☐
201. [VN: **HIVPLEASU**]Condoms can be pleasurable	☐	☐	☐	☐	☐
202. [VN: **HIVVALUE**]Condoms go against my values or religious beliefs	☐	☐	☐	☐	☐

QUESTIONS HIVRISK - HIVNOTRI ARE ONLY FOR MEN WHO ARE <u>HIV-NEGATIVE</u>. IF HIV-POSITIVE, CONTINUE WITH INTRO ABOVE HIVGETEAS

To what extent do you agree or disagree with the following statements?	Disagree strongly 1	Disagree 2	Agree 3	Agree strongly 4	No answer 99
203. [VN: **HIVRISK**] I am at risk for HIV	☐	☐	☐	☐	☐
204. [VN: **HIVPOSS**] There is a possibility that I have HIV/AIDS	☐	☐	☐	☐	☐
205. [VN: **HIVSEX**] I may have had sex with someone who was at risk for HIV	☐	☐	☐	☐	☐
206. [VN: **HIVNOTRI**] My sexual experiences do not put me at risk for HIV	☐	☐	☐	☐	☐

[SHOW IF SELF-ADMINISTERED]: Thank you for completing that section on your own. Please tell the Study Staff that you have finished your section and are ready to continue the interview.

[SHOW IF INTERVIEWER ADMINISTERED]: Thank you for completing that section.

INTERVIEWER RADIO BUTTON

- **INTERVIEWER ADMINISTERED**

INSERT PAGE BREAK

[SHOW ALL]: I will now continue to read questions to you about HIV. Remember you can ask me questions at any time and we can skip any question you prefer to not answer. Your responses are very important to this research study. Do you have any questions before we begin?

VN: **HIVGETEAS**

207. Is it easy or hard to get condoms where you live?

1☐ Very easy → **GO TO HIVGETLUBE**
2☐ Easy → **GO TO HIVGETLUBE**
3☐ Not easy, not hard → **GO TO HIVGETLUBE**
4☐ Hard
5☐ Very hard
6☐ Don't know
99☐ No answer

SHOW IF HIVGETEAS = 4 OR 5
VN: **HIVGETEAS_OPEN**

208. What makes it hard for you to get condoms? **[OPEN TEXT]**

____

99☐ No answer

VN: **HIVGETLUB**

209. Is it easy or hard to get water-based lubricants where you live?

1☐ Very easy → **GO TO HIVTALKS**
2☐ Easy → **GO TO HIVTALKS**
3☐ Not easy, not hard → **GO TO HIVTALKS**
4☐ Hard
5☐ Very hard
6☐Don't know
99☐ No answer

SHOW IF HIVGETLUBE = 4 or 5
VN: **HIVGETLUB_OPEN**

210. What makes it hard for you to get water-based lubricants? **[OPEN TEXT]**

____

99☐ No answer

VN: **HIVTALKS**

211. In the last 12 months, have you participated in any talks or meetings about HIV/AIDS?
1☐ Yes
2☐ No
99 ☐ No answer

[VN: **HIVMSM**]

212. In the last 12 months, have you participated in any talks or meetings related to HIV/AIDS and the issues of MSM?
1☐ Yes
2☐ No
99 ☐ No answer

[VN: **HIVPREV**]

213. In the last 12 months, have you received any information on HIV prevention?
1☐ Yes
2☐ No → **GO TO HIVDIRTY**
99 ☐ No answer → **GO TO HIVDIRTY**

SHOW IF HIVPREV=1
I will now read you a list of different types of information. For each type, please tell me whether you have received this information or not in the past 12 months. Did you receive information…

	Yes=1	No=2	No answer = 99
214. [VN: **HIVPREVW**] About how to prevent HIV transmission when having sex with women	☐	☐	☐
215. [VN: **HIVPREVM**] About how to prevent HIV transmission when having sex with men	☐	☐	☐
216. [VN: **HIVPREVN**] About how to prevent HIV using dirty needles	☐	☐	☐
217. [VN: **HIVPREVA**] About abstaining from having sex	☐	☐	☐
218. [VN: **HIVPREVT**] About treatment for HIV	☐	☐	☐

IF HIVPREVW - HIVPREVT ALL = 2 (NO) GO TO HIVDIRTY

SHOW IF HIVPREVW=1 or HIVPREVM =1 or HIVPREVN=1 or HIVPREVA = 1 or HIVPREVT=1
If you have received information on prevention of HIV transmission, where did you get it? I will read several potential sources of information. Please let me know if you have received information on prevention of HIV transmission from each of the following sources.

	Yes=1	No=2	No answer = 99
219. [VN: **HIVPREVF**] A magazine, brochure, or flyer	☐	☐	☐
220. [VN: **HIVPREVS**] School	☐	☐	☐
221. [VN: **HIVPREW**] Peer educator or workshop	☐	☐	☐
222. [VN: **HIVPREI**] Internet	☐	☐	☐
223. [VN: **HIVPRED**] Doctor or other healthcare provider	☐	☐	☐
224. [VN: **HIVPREC**] Mosque, church, or religious group	☐	☐	☐
225. [VN: **HIVPREFF**] Friends or family	☐	☐	☐

226. [VN: **HIVPREO**] Other men who have sex with men	☐	☐	☐
227. [VN: **HIVPREBB**] Media or billboard	☐	☐	☐

VN: **HIVPREOTH**

228. Have there been any other sources that you have received information from on HIV prevention in the last 12 months?

1☐ Yes, specify ____________________ **[TEXT]**
2☐ No
99☐ No answer

SHOW ALL

To what extent do you agree or disagree with the following statements? Would you say you Disagree strongly, Disagree, Agree or Agree strongly that….	Disagree strongly =1	Disagree =2	Agree =3	Agree strongly =4	No answer =99
229. [VN: **HIVDIRTY**] Most people believe that a person who has HIV is dirty and unclean	☐	☐	☐	☐	☐
230. [VN: **HIVREJECT**] Most people with HIV are rejected when others find out	☐	☐	☐	☐	☐
231. [VN: **HIVLJOB**] People with HIV lose their jobs when their employers find out	☐	☐	☐	☐	☐
232. [VN: **HIVDISGU**] Most people think that a person with HIV is disgusting	☐	☐	☐	☐	☐

HIV-Related Care and Treatment and HIV Stigma

SHOW CTSDOCTR - CTSWJUDG (THIS SECTION) ONLY IF PARTICIPANT IS HIV-POSITIVE. TO BE ASKED AFTER REFERRAL TO CARE HAS BEEN MADE.

VN: **CTSDOCTR**

233. Since your last visit, have you seen a doctor for the treatment of your HIV infection?
1☐ Yes → **GO TO CTSDRRES**
2☐ No
99 ☐ No Answer

SHOW IF CTSDOCTR = 2
VN: **CTSDOCNO_OPEN**

234. Can you explain why you haven't seen a doctor for the treatment of your HIV infection? **[OPEN TEXT]**

__________________ → **GO TO SELF-ADMINISTERED SECTION INTRO ABOVE CTSDRUNC**

99☐ No answer

SHOW CTDRRES – CTSMEDMIS IF CTSDOCTR = 1 (YES)
VN: **CTSDRRES**

235. When you went for treatment, did you feel respected by the doctor and nurses?
1☐ Yes
2☐ No
99 ☐ No Answer

VN: **CTSDRUND**

236. Do you feel that the doctor and nurses understood you?
1☐ Yes
2☐ No
99 ☐ No Answer

VN: **CTSDRACC**

237. Do you think the doctor and the nurses accept or reject sex between men?
1☐ Reject
2☐ Accept
3☐ Neutral
4☐ I don't know
99 ☐ No Answer

VN: **CTSDRMED**

238. Did the doctor prescribe you any medication for the treatment of your HIV infection?
1☐ Yes
2☐ No → **GO TO SELF-ADMINISTERED SECTION INTRO ABOVE CTSDRUNC**
99 ☐ No Answer

SHOW CTSMEDPRE – CTSMEDMIS IF CTSDRMED = 1 (YES)

VN: **CTSMEDPRE**

239. Do you know what medication the doctor prescribed (for the treatment of HIV)?

Note: Make one comprehensive list of response options but only display certain options based on Site. Allow participant to select all drugs that apply.

If Site = Malawi:
1☐AZT
2☐3TC
3☐NVP
4☐EFV
5☐ATV/r
6☐LPV,r
7☐TDF
8☐Combivir (AZT, 3TC)
9☐Truvada (TDF, FTC)
10☐Single tablet TDF
11☐ATV/r
12☐D4T
13☐Triomune (D4T, 3TC, NVP) in single tablet
15☐Raltegrevir
15☐Cotrimoxazole

If Site = Soweto or Cape Town:
16☐Efavirenz
17☐Nevirapine
18☐Combivir (AZT and 3TC)
19☐Lamivudine
20☐FDC (fixed dose combination – Tenofovir, EFV, 3TC),
21☐Tenofovir
22☐Ritonavir
23☐Zidovudine
24☐Alluvia (RTV/LPV)
25☐Kaletra
26☐Abacavir

If Site = Kenya:
27☐Fixed dose regimen - AZT / 3TC
28☐AZT /3TC/ +NVP
29☐D4T / 3TC
30☐D4T / 3TC/ NVP
31☐TDF /3TC
32☐TDF/ 3TC/ EFV
33☐Didanosine
34☐Efavirenz
35☐Nevirapine
36☐Ritonavir
37☐Lopinavir
38☐Atazanavir
39☐Darunavir
40☐Raltegravir
41☐Elvitegravir
42☐Emtricitabine
43☐Lamivudine
44☐Stavudine
45☐Zidovudine
99☐ No Answer **(SHOW ALL SITES THIS OPTION)**

VN: **CTSMEDOFT**

240. How often do you have to take the medication? **[OPEN TEXT]**

____

99☐ No answer

VN: **CTSMEDPIL**

241. Many people find it difficult to take their pills every day. In the last 30 days, how good a job did you do at taking your HIV medicines in the way you were supposed to? Would you say…

1☐ Very poor
2☐ Poor
3☐ Fair
4☐ Good
5☐ Very good
6☐ Excellent
99 ☐ No Answer

VN: **CTSMEDSCA**

242. Please indicate on a scale from 0 to 100 your best guess about how much of your HIV medicines you took as recommended over the last 30 days. 0% means that you have taken no pills, 50% means you have taken half of your pills, and 100% means you have taken every single pill.

☐ 0
☐ 10
☐ 20
☐ 30
☐ 40
☐ 50
☐ 60
☐ 70
☐ 80
☐ 90
☐ 100
99 ☐ No Answer

VN: **CTSMEDMIS**

243. In the last 30 days, on how many days did you miss at least one dose of your HIV medicines?

__________________ days (range 0 - 30)
99☐ No answer

At this point, you may read and record your responses on your own again. However, some people prefer that I continue to read the questions out loud and record their responses for them. Which would you prefer?

INTERVIEWER RADIO BUTTON

- **SELF-ADMINISTERED**
- **INTERVIEWER ADMINISTERED**

PAGE BREAK

You will now be asked to respond to some questions about your experiences living with HIV. Your honest responses are very important to this study. [**IF SELF-AMINISTERED:** If you have any questions, please ask the Interviewer.]

Since you found out that you have HIV, has any healthcare provider . . .	Yes = 1	No = 2	No Answer = 99
244. [VN: **CTSDRUNC**]. . . Been uncomfortable with you?	☐	☐	☐
245. [VN: **CTSDRINF**].. . . Treated you as inferior?	☐	☐	☐
246. [VN: **CTSDRAVO**].. . . Preferred to avoid you?	☐	☐	☐
247. [VN: **CTSDRREF**].. . . Refused to see you for medical care?	☐	☐	☐

To what extent do you agree or disagree with the following statements?	Disagree strongly =1	Disagree =2	Agree =3	Agree strongly =4	No answer = 99
248. [VN: **CTSTELLP**] It is difficult to tell people about my HIV infection	☐	☐	☐	☐	☐
249. [VN: **CTSDIRTY**] Being HIV positive makes me feel dirty and unclean	☐	☐	☐	☐	☐
250. [VN: **CTSGUILT**] I feel guilty that I am HIV positive	☐	☐	☐	☐	☐
251. [VN: **CTSASHAM**] I am ashamed that I am HIV positive	☐	☐	☐	☐	☐
252. [VN: **CTSWORTH**] I sometimes feel worthless because I am HIV positive	☐	☐	☐	☐	☐
253. [VN: **CTSIHIDE**] I hide my HIV status from others	☐	☐	☐	☐	☐

To what extent do you agree or disagree with the following statements?	Disagree strongly = 1	Disagree = 2	Agree = 3	Agree strongly =4	No answer = 99
254. [VN: **CTSREJECT**] Some people close to me are afraid others will reject them if it becomes known that I have HIV	☐	☐	☐	☐	☐
255. [VN: **CTSBACKA**] People have physically backed away from me when they learn I have HIV	☐	☐	☐	☐	☐
256. [VN: **CTSIGOODP**] People who know I have HIV tend to ignore my good points	☐	☐	☐	☐	☐
257. [VN: **CTSTOUCH**] Some people avoid touching me once they know I have HIV	☐	☐	☐	☐	☐
258. [VN: **CTSOCIAL**] I have stopped socializing with some people because of their reactions to my having HIV	☐	☐	☐	☐	☐
259. [VN: **CTSSTOP**] People I care about stopped calling me after learning I have HIV	☐	☐	☐	☐	☐
260. [VN: **CTSAFRAI**] People seem afraid of me once they learn I have HIV	☐	☐	☐	☐	☐
261. [VN: **CTSHURT**] I have been hurt by how people reacted to learning I have HIV	☐	☐	☐	☐	☐
262. [VN: **CTSCHILD**] People don’t want me around their children once they know I have HIV	☐	☐	☐	☐	☐
263. [VN: **CTSFRIEN**] I have lost friends by telling them I have HIV	☐	☐	☐	☐	☐
264. [VN: **CTSDISTA**] Some people who know I have HIV have grown more distant	☐	☐	☐	☐	☐
265. [VN: **CTSUNCLE**] Having HIV makes me feel unclean	☐	☐	☐	☐	☐

266. [VN: **CTSIMBAD**] Having HIV makes me feel that I'm a bad person	☐	☐	☐	☐	☐
267. [VN: **CTSWORSE**] People's attitudes about HIV make me feel worse about myself	☐	☐	☐	☐	☐
268. [VN: **CTSNAGAP**] I feel I am not as good a person as others because I have HIV	☐	☐	☐	☐	☐
269. [VN: **CTSFAULT**] Some people act as though it's my fault that I have HIV	☐	☐	☐	☐	☐
270. [VN: **CTSISOLA**] Since learning I have HIV, I feel set apart and isolated from the rest of the world	☐	☐	☐	☐	☐
271. [VN: **CTSNHIDE**] I never feel the need to hide the fact that I have HIV	☐	☐	☐	☐	☐
272. [VN: **CTSWTELL**] I worry that people who know I have HIV will tell others	☐	☐	☐	☐	☐
273. [VN: **CTSRTOLD**] I regret having told some people that I have HIV	☐	☐	☐	☐	☐
274. [VN: **CTSWJUDG**] I worry that people may judge me when they learn I have HIV	☐	☐	☐	☐	☐

Alcohol and Drugs

[SHOW IF HIV CARE SECTION WAS SKIPPED (I.E. IF HIV-NEGATIVE) AND THIS IS THE FIRST TIME THE PARTICIPANT IS BEING ASKED IF THEY WANT TO SWITCH TO SELF-ADMINISTERED]: At this point, you may read and record your responses on your own again. However, some people prefer that I continue to read the questions out loud and record their responses for them. Which would you prefer?

***OTHERWISE, MAINTAIN SELF-ADMINISTERED OR INTERVIEWER-ADMINISTERED SETTING**

INTERVIEWER RADIO BUTTON

- **SELF-ADMINISTERED**
- **INTERVIEWER ADMINISTERED**

[SHOW IF SELF-ADMINISTERED]: You will now be asked to respond to some questions about your experiences with alcohol and drugs. Your honest responses are very important to this study. If you have any questions, please ask the Interviewer.

[SHOW IF INTERVIEWER ADMINISTERED]: The next questions are about the use of alcohol and drugs. Please choose the best response.

VN: **AODOFTEN**

275. How often do you have a drink containing alcohol?
1☐ Never → **GO TO AODPXDG**
2☐ Monthly or less
3☐ Two to four times a month
4☐ Two to three times a week
5☐ Four or more times a week
99☐ No answer

SHOW IF AODOFTEN IS >1 (IF PARTICIPANT HAS HAD A DRINK)

VN: **AODMANY**

276. How many drinks containing alcohol do you have on a typical day when you are drinking?
1☐ 1 or 2
2☐ 3 or 4
3☐ 5 or 6
4☐ 7 to 9
5☐ 10 or more
99☐ No answer

SHOW IF AODOFTEN IS >1 (IF PARTICIPANT HAS HAD A DRINK)

VN: **AODBINGE**

277. How often do you have six or more drinks on one occasion?
1 ☐Never
2 ☐Less than monthly
3 ☐Monthly
4 ☐Weekly
5 ☐Daily or almost daily
99☐ No answer

The next questions are about other substances you could have used, including substances prescribed by a doctor (like pain medications) that you might have taken for reasons or in doses other than prescribed, and recreational or illegal drugs.

When referring to these substances, please include any of the following:
Cannabis (marijuana, dagga, bhang, ganja, puga, pot, grass, hash, etc.)
Nyaope (whoonga or wunga; ARV mixed with other drugs)
Inhalants (turpentine, nitrous oxide, shoe glue, glue, petrol, gas, paint thinner, etc.)
Methamphetamine (tik, speed, crystal meth, ice, etc.)
Sedatives or sleeping pills (mandrax, Valium, Serepax, Ativan, Xanax, Librium, Rohypnol, GHB, etc.)
Cocaine (coke, crack, etc.)
Street opioids (heroin, opium, etc.)
Prescription opioids (fentanyl, oxycodone [OxyContin, Percocet], hydrocodone [Vicodin], methadone, buprenorphine, etc.).
Prescription stimulants (Ritalin)

	Never 1	Once or twice 2	Monthly 3	Weekly 4	Daily or almost daily 5	No answer 99
278. [VN: **AODPXDG**] In the past year, how often have you used prescription drugs for non-medical reasons?	☐	☐	☐	☐	☐	☐
279. [VN: **AODRECD**] In the past year, how often have you used recreational drugs?	☐	☐	☐	☐	☐	☐

SHOW IF AODPXDG > 1 OR AODRECD > 1 (IF DRUGS HAVE EVER BEEN USED)

VN: **AODHUSE**

280. Some people smoke, swallow, or inject drugs. How do you use drugs? You can choose more than one response

AODHUSE_1☐ Smoke
AODHUSE_2☐ Swallow
AODHUSE_3☐ Inject
AODHUSE_4☐ Other
AODHUSE_99☐ No answer

SHOW IF AODHUSE_3 IS CHECKED

VN: **AODINJECT**

281. Have you in the past three months ever shared needles with somebody else?

1☐ Yes
2☐ No
99☐ No answer

Mental Health

*MAINTAIN SELF-ADMINISTERED OR INTERVIEWER-ADMINISTERED SETTING

The next questions are about how you feel about yourself.

VN: **MHSELFEST**

282. To what extent do you agree or disagree with the following statement:

“I have high self-esteem”? With “self-esteem” we mean having a favorable opinion of yourself.

1☐ Disagree strongly
2☐ Disagree
3☐ Agree
4☐ Agree strongly
99☐ No answer

VN: **MHANXIET**

283. In the last 4 weeks, since **{TODAY-4 weeks},** have you had an anxiety attack — suddenly feeling fear or panic?

1☐ Yes
2☐ No → **GO TO DEPNOINT**
99☐ No answer

SHOW IF MHANXIET = 1

VN: **MHANXEVR**

284. Has this ever happened before?

1☐ Yes
2☐ No
99☐ No answer

Over the last 2 weeks, how often have you been bothered by any of the following problems?

[IF INTERVIEWER ADMIN]: For each please respond with Not at all, Several days, More than half the days or Nearly everyday.

	Not at all 1	Several days 2	More than half the days 3	Nearly everyday 4	No answer 99
285. [VN: **DEPNOINT**] Little interest or pleasure in doing things	☐	☐	☐	☐	☐
286. [VN: **DEPRESSD**] Feeling down, depressed, or hopeless	☐	☐	☐	☐	☐
287. [VN: **DEPSLEEP**]Trouble falling or staying asleep, or sleeping too much	☐	☐	☐	☐	☐
288. [VN: **DEPTIRED**] Feeling tired or having little energy	☐	☐	☐	☐	☐
289. [VN: **DEPOREAT**] Poor appetite or overeating	☐	☐	☐	☐	☐
290. [VN: **DEPDOWN**] Feeling bad about yourself - or that you are a failure or have let yourself or your family down	☐	☐	☐	☐	☐
291. [VN: **DEPTHINK**] Trouble	☐	☐	☐	☐	☐

concentrating on things, such as reading the newspaper or watching television					
292. [VN: **DEPMOVE**] Moving or speaking so slowly that other people could have noticed	☐	☐	☐	☐	☐
293. [VN: **DEPRESTL**] Or the opposite - being so fidgety or restless that you have been moving around a lot more than usual	☐	☐	☐	☐	☐
294. [VN: **DEPHURTS**] Thoughts that you would be better off dead, or of hurting yourself in some way	☐	☐	☐	☐	☐

Opinions About Self

***MAINTAIN SELF-ADMINISTERED OR INTERVIEWER-ADMINISTERED SETTING**

Please say whether you think the following statements are true or false.	True 1	False 2	No answer 99
295. [VN: **MHRESENT**] I sometimes feel resentful when I don't get my way	☐	☐	☐
296. [VN: **MHGIVUP**] On a few occasions, I have given up doing something because I thought too little of my ability	☐	☐	☐
297. [VN: **MHREBEL**] There have been times when I felt like rebelling against people in authority even though I knew it wouldn't get me anywhere	☐	☐	☐
298. [VN: **MHLISTE**] No matter who I am talking to, I'm always a good listener	☐	☐	☐
299. [VN: **MHSICK**] I can remember "playing sick" to get out of something	☐	☐	☐
300. [VN: **MHADVAN**] There have been occasions when I took advantage of someone	☐	☐	☐
301. [VN: **MHMISTA**] I am always willing to admit it when I make a mistake	☐	☐	☐
302. [VN: **MHGEVEN**] I sometimes try to get even, rather than forgive and forget	☐	☐	☐
303. [VN: **MHCOURT**] I am always courteous, even to people who are disagreeable	☐	☐	☐
304. [VN: **MHANNOYE**] I have never been annoyed when people expressed ideas very different from my own	☐	☐	☐
305. [VN: **MHJEALOU**] There have been times when I was quite jealous of the good fortune of others	☐	☐	☐
306. [VN: **MHIRRITA**] I am sometimes irritated by people who ask favors of me	☐	☐	☐
307. [VN: **MHNOMEAN**] I have never deliberately said something that hurt someone's feelings	☐	☐	☐

Experience of Study Participation

***MAINTAIN SELF-ADMINISTERED OR INTERVIEWER-ADMINISTERED SETTING**

The following questions are about your participation in the study. The first set of questions is about how you felt answering the questions in the interview.

How did you feel about answering the questions? To what extent do you agree or disagree with the following statements?	Disagree strongly 1	Disagree 2	Agree 3	Agree strongly 4	No answer 99
308. [VN: **PRTQSAD**] Some questions made me sad	☐	☐	☐	☐	☐
309. [VN: **PRTQLIKE**] I liked it that I was able to give my opinion	☐	☐	☐	☐	☐
310. [VN: **PRTQEASY**] All questions were easy to understand	☐	☐	☐	☐	☐
311. [VN: **PRTQDIFF**] Some questions were difficult to answer	☐	☐	☐	☐	☐
312. [VN: **PRTQDOWN**] Some questions made me feel down	☐	☐	☐	☐	☐
313. [VN: **PRTQHELP**] I felt a need for help due to the questions	☐	☐	☐	☐	☐
314. [VN: **PRTQRELIE**] I found it a relief to share my experiences	☐	☐	☐	☐	☐
315. [VN: **PRTQBADT**] The questions gave me bad thoughts about things that happened to me	☐	☐	☐	☐	☐

VN: **PRTQSEXB**

316. How comfortable or uncomfortable did you feel answering the questions about sexual behavior?
   1☐ Very comfortable
   2☐ Comfortable
   3☐ Uncomfortable
   4☐ Very uncomfortable
   99☐ No answer

VN: **PRTQAOD**

317. How comfortable or uncomfortable did you feel answering the questions about drug and alcohol use?
   1☐ Very comfortable
   2☐ Comfortable
   3☐ Uncomfortable
   4☐ Very uncomfortable
   99☐ No answer

The next set of questions is about your participation in this study more generally.

Please indicate whether you agree or disagree with the following statements:	Disagree strongly	Disagree	Agree	Agree strongly	No answer 99
318. [VN: **PRTCLEAR**] All study procedures are clearly explained	☐	☐	☐	☐	☐
319. [VN: **PRTRESPT**] I feel respected by the research staff	☐	☐	☐	☐	☐
320. [VN: **PRTPRIVT**] The research staff keeps my personal information completely private	☐	☐	☐	☐	☐

[IF SELF-ADMINISTERED]: Thank you. You have finished this self-administered section. Please tell the clinic staff person that you have finished your section and are ready to continue the interview.

[IF INTERVIEWER ADMINISTERED]: Thank you for completing that section.

INTERVIEWER RADIO BUTTON

- **INTERVIEWER ADMINISTERED**

I will now continue to read questions to you about your experiences with this study. Do you have any questions before we begin?

VN: **PRTCOMFY**

321. Do you feel comfortable with all the medical procedures requested for this study?
1☐ Yes → **GO TO PRTRJOIN**
2☐ No
99☐ No answer

SHOW IF PRTCOMFY = 2

VN: **PRTMPROC**

322. Can you tell which medical procedures you are not comfortable with and why that is so? **[OPEN TEXT]**

____
99☐ No answer

SHOW ALL

VN: **PRTRJOIN**

323. What was the single most important reason you decided to join this study? **[OPEN TEXT]**

____
99☐ No answer

VN: **PRTRCONT**

324. What is the single most important reason you continue to participate in this study? **[OPEN TEXT]**

____
99☐ No answer

VN: **PRTCOMM**

325. How committed do you feel to this study?
1☐ Very committed
2☐ Committed
3☐ Moderately committed
4☐ Not committed
99☐ No answer

VN: **PRTIMPORT**

326. How important or unimportant do you consider this study to be for your community?
1☐ Very Important
2☐ Important

3☐ Moderately important
4☐ Not important at all
99☐ No answer

There are several things that might make it difficult for you to participate in this study. I will mention a few things and I would like to hear from you whether it is easy or difficult for you to do these.

	Very easy 1	Easy 2	Difficult 3	Very difficult 4	No answer 99
327. [VN: **PRTCLINC**] Travelling to the study clinic	☐	☐	☐	☐	☐
328. [VN: **PRTTIME**] Making time to come for study visits	☐	☐	☐	☐	☐
329. [VN: **PRTAPPTS**] Setting up appointments for a study visit	☐	☐	☐	☐	☐

VN: **PRTBURDN**

330. Overall, considering the time you spent and any anxiety, or discomfort you may have experienced in this study, how difficult has it been for you to participate?
4☐Very difficult
3☐Moderately difficult
2☐Slightly difficult
1☐Not difficult at all
99☐ No answer

SHOW IF PRTBURDN>1
VN: **PRTSIGBRD**

331. What are the most significant burdens thus far for you of participating in this study? **[OPEN TEXT]**

____

99☐ No answer

VN: **PRTOTHER**

332. Do you know any men like you who also have sex with men who are not participating in this study?
1☐ Yes
2☐ No → **GO TO PRTFAMLY**
99☐ No answer

VN: **PRTOTH_OPEN**

333. Do you have any idea why these men are not participating in this study? **[OPEN TEXT]**

____

99☐ No answer

Because of your participation in this study, have you, since your last visit…	Yes 1	No 2	No answer 99
334. [VN: **PRTFAMLY**]. . . Had personal trouble with family?	☐	☐	☐

335. [VN: **PRTFRIEND**]. . . Had personal trouble with friends or acquaintances?	☐	☐	☐
336. [VN: **PRTHOUSE**]. . . Had trouble getting or keeping housing?	☐	☐	☐
337. [VN: **PRTECON**]. . . Had trouble getting or keeping a job or trouble with income or economic support?	☐	☐	☐
338. [VN: **PRTHEALTH**]. . . Had trouble with health insurance or getting health care?	☐	☐	☐
339. [VN: **PRTLEGAL**]. . . Been arrested or had trouble with the police or other legal problems?	☐	☐	☐
340. [VN: **PRTPROB**]. . . Had any other type of problem?	☐	☐	☐

SHOW IF ANY ONE OF PRTFAMLY TO PRTPROB = 1 (YES)

VN: **PRTEVENT**

341. How often have these events happened since your last visit? Would you say…
1☐ Only once
2☐ A few times
3☐ Regularly
4☐ Often
5☐ Very often
99☐ No answer

SHOW ALL
VN: **PRTEVENT_OPEN**

342. Because of your participation in this study, did anything negative or bad happen to you that you have not reported to us already? **[OPEN TEXT]**

____
99☐ No answer

END SCREEN
Thank you for your participation in the enrollment interview.

HPTN 075 Follow-up Questionnaire Version 4.0 – May 25, 2016

Contents

Relationship Status and Demographic Background 3
Sexual and Gender Identity, and Social Support 5
Resilience and Coping Self-Efficacy 8
HIV Prevention 10
Sexual Behavior Assessment 12
HIV Risk Perception 31
HIV/STI-Related Factors 32
HIV-Related Care and Treatment and HIV Stigma 35
Interest in HIV Prevention Strategies 41
Alcohol and Drugs 46
Mental Health 49
Experience of Study Participation 51

Self-Administered vs. Interviewer-Administered

Relationship Status and Demographic Background	Interviewer Administered
Sexual and Gender Identity and Social Support	Interviewer Administered
Resilience and Copying Self-Efficacy	Interviewer Administered
HIV Prevention	Interviewer Administered
Sexual Behavior Assessment	Interviewer Administered
HIV Risk Perception	Interviewer Administered
HIV/STI-Related Factors	Self-Administered HIVAIDS through HIVNOTRI, then back to Interviewer-Administered
HIV-Related Care and Treatment and HIV Stigma	Interviewer Administered; Self-Administered will resume at CTSDRUNC
Interest in HIV Prevention Strategies	Interviewer Administered
Alcohol and Drugs	Self-Administered
Mental Health	Self-Administered
Experience of Study Participation	Self-Administered stops after PRTPRIVT, then back to Interviewer Administered

PTID

Participant ID

_ _ _ - _ _ _ _ _ - _

Site Number | Participant Number | Chk

VISIT CODE

_ _ . _

SURVEY LANGUAGE

- English (*Show every time)*
- Xhosa (*Only show for Cape Town site)*
- Afrikaans (*Only show for Cape Town site)*
- Sutu *(Only show for Soweto site)*
- Zulu *(Only show for Soweto site)*
- Swahili *(Only show for Kenya site)*
- Luo *(Only show for Kenya site)*
- Chichewa *(Only show for Malawi site)*

Variable Name (VN): **HIVSTATUS**

Interviewer Instructions: Input HIV Status from study HIV testing (not self-reported by participant)

HIV Status

- HIV-Negative
- HIV-Positive

Relationship Status and Demographic Background

	Week 13/ 3.0	Week 26/ 4.0	Week 39/ 5.0	Week 52/ 6.0
**Relationship Status and Demographic Background**	**X**	**X**	**X**	**X**
Sexual and Gender Identity, and Social Support		X		X
Resilience and Coping Self-Efficacy		X		
HIV Prevention	X	X	X	X
Sexual Behavior Assessment	X	X	X	X
HIV Risk Perception	X		X	
HIV/STI-Related Factors		X		X
HIV-Related Care, Treatment and HIV Stigma	X	X	X	X
Interest in HIV Prevention Strategies		X		
Alcohol and Drugs		X		X
Mental Health	X	X	X	X
Experience of Study Participation	X	X	X	X

SHOW (DEMEMPS) IF VISIT= 4.0 (WEEK 26) OR 6.0 (WEEK 52)

VN: **(DEMEMPS)**

1. What best describes your current employment status?

1☐ Employed full-time
2☐ Employed part-time
3☐ Self-employed
4☐ Unemployed or between jobs
5☐ On disability
6☐ Other
99☐ No answer

SHOW DEMPREV – (MPARTLHH) IF VISIT =3.0 (WEEK 13), 4.0 (WEEK 26), 5.0 (WEEK 39), OR 6.0 (WEEK 52)

VN: **DEMPREV**

2. Did you mention at your last visit that you were in an on-going, intimate, sexual relationship with a man?

1 ☐ Yes → **GO TO DEMPREVP**
2 ☐ No → **GO TO DEMOTHER**

VN: **DEMPREVP**

3. Are you still with the partner you mentioned at the previous visit?

1 ☐ Yes → **GO TO NEXT SECTION (HIV PREVENTION IF VISIT = 3.0 OR 5.0; SEXUAL AND GENDER IDENTITY, ETC. IF VISIT = 4.0 OR 6.0)**
2 ☐ No
99 ☐ No answer → **GO TO NEXT SECTION (HIV PREVENTION IF VISIT = 3.0 OR 5.0; SEXUAL AND GENDER IDENTITY, ETC. IF VISIT = 4.0 OR 6.0)**

SHOW IF DEMPREVP = 2 (NO)

VN: **DEMIDEA**

4. Have you any idea why your relationship with your former partner ended?

_______________________________ **[OPEN TEXT]**
2 ☐ No
99 ☐ No answer

SHOW IF DEMPREVP = 2 (NO)
VN: **DEMEXPLAIN**

5. Could it be that your participation in this research study has anything to do with you and your partner breaking up?
   1 ☐ Yes
   2 ☐ No → **GO TO DEMEXPLAIN**
   99 ☐ No answer → **GO TO DEMOTHER**

SHOW IF DEMEXPLAIN = 1 (YES)
VN: **DEMEXPLAIN.TEXT**

6. Please explain how participating in the research related to you and your partner breaking up.
   ______________________________ **[OPEN TEXT]**
   99 ☐ No answer

VN: **DEMOTHER**

7. Are you currently in an (other) ongoing, intimate, sexual relationship with a man?
   1 ☐ Yes
   2 ☐ No → **GO TO NEXT SECTION (HIV PREVENTION IF VISIT = 3.0 OR 5.0; SEXUAL AND GENDER IDENTITY, ETC. IF VISIT = 4.0 OR 6.0)**
   99 ☐ No answer → **GO TO NEXT SECTION (HIV PREVENTION IF VISIT = 3.0 OR 5.0; SEXUAL AND GENDER IDENTITY, ETC. IF VISIT = 4.0 OR 6.0)**

SHOW IF DEMOTHER = 1 (YES)
VN: **(DEMPTIME)**

8. How long have you been in this relationship?
   DEMPTIME_LESS 1☐ Less than a month
   DEMPTIME_MM ___Months (*min 1, max 11*) **[Note: Allow participant to enter both months and years]**
   DEMPTIME_YY ___Years (*min 1, max 99*)
   99☐ No answer

SHOW IF DEMOTHER= 1 (YES)
VN: **(MPARTLHH)**

9. Do you live together in the same household?
   1☐ Yes
   2☐ No
   99☐ No answer

Sexual and Gender Identity, and Social Support

	Week 13/ 3.0	Week 26/ 4.0	Week 39/ 5.0	Week 52/ 6.0
Relationship Status and Demographic Background	X	X	X	X
**Sexual and Gender Identity, and Social Support**		**X**		**X**
Resilience and Coping Self-Efficacy		X		
HIV Prevention	X	X	X	X
Sexual Behavior Assessment	X	X	X	X
HIV Risk Perception	X		X	
HIV/STI-Related Factors		X		X
HIV-Related Care and Treatment and HIV Stigma	X	X	X	X
Interest in HIV Prevention Strategies		X		
Alcohol and Drugs		X		X
Mental Health	X	X	X	X
Experience of Study Participation	X	X	X	X

SHOW (IDMASCUL) – (IDHIDDEN2) IF VISIT = 6.0 (WEEK 52)

VN: **(IDMASCUL)**

10. If you compare yourself to others, would you say you are generally more like other men or more like women? Would you say you are…
    - 1☐ Like other men
    - 2☐ Like other men as well as like women
    - 3☐ More like women
    - 4☐ The same as women
    - 99☐ No answer

VN: **(IDATTRACT)**

11. Do you currently feel more sexually attracted to men or to women? Would you say…
    - 1☐ Only to women
    - 2☐ More to women than to men
    - 3☐ To women and men equally
    - 4☐ More to men than to women
    - 5☐ Only to men
    - 99☐ No answer

VN: **(IDSEXUAL)**

12. Do you identify as gay, bisexual, heterosexual, or transgender, or would you use another word to describe your sexuality?
    - 1☐ Gay → **GO TO IDDECIDE**
    - 2☐ Bisexual → **GO TO IDDECIDE**
    - 3☐ Heterosexual
    - 4☐ Transgender → **GO TO IDDECIDE**
    - 5☐ Other → **GO TO IDDECIDE**
    - 99☐ No answer → **GO TO IDDECIDE**

SHOW IF (IDSEXUAL) = 3 (HETEROSEXUAL)
VN: **(IDEXPLAIN)**

13. You told us that you have sex with men and you call yourself heterosexual. Could you explain why you see yourself that way?

____________________ **[OPEN TEXT]**
2 ☐ No
99 ☐ No answer

To what extent do you agree or disagree with the following statements? For each indicate if you disagree strongly, disagree, agree or agree strongly.

	Disagree strongly 1	Disagree 2	Agree 3	Agree strongly 4	No answer 99
14. [VN: **(IDDECIDE)**] I cannot decide whether I am bisexual, homosexual, or heterosexual	☐	☐	☐	☐	☐
15. [VN: **(IDISLIKE)**] Sometimes I dislike myself for being a man who has sex with other men	☐	☐	☐	☐	☐
16. [VN: **(IDWISH)**] I wish I were only sexually attracted to women	☐	☐	☐	☐	☐
17. [VN: **(IDGLAD)**] I am glad to be sexually attracted to other men	☐	☐	☐	☐	☐

SHOW IF (IDSEXUAL) ≠ 3 (NOT 3, HETEROSEXUAL)

VN: **(IDHIDDEN)**

18. In general, how hard do you try to keep your sexual orientation hidden from your family?

1☐ Try very hard
2☐ Try somewhat hard
3☐ Don't try, but don't talk about it
4☐ I openly talk about it with my family
98☐ Not applicable
99☐ No answer

SHOW IF (IDSEXUAL) = 3 (HETEROSEXUAL)

VN: **(IDHIDDEN2)**

19. In general, how hard do you try to keep it hidden from your family that you have sex with men?

1☐ Try very hard
2☐ Try somewhat hard
3☐ Don't try, but don't talk about it
4☐ I openly talk about it with my family
98☐ Not applicable
99☐ No answer

SHOW STIGHARA – SSCOMMUN IF VISIT = 4.0 (WEEK 26) OR 6.0 (WEEK 52)

Have you, as a result of sexual orientation or practice, in the last 6 months. . .	Yes = 1	No = 2	No Answer = 99
20. [VN: **(STIGHARA)**]…Been verbally or physically harassed?	☐	☐	☐
21. [VN: **(STIGBEAT)**]…Been beaten up?	☐	☐	☐
22. [VN: **(STIGBLACK)**]…Been blackmailed?	☐	☐	☐
23. [VN: **(STIGLOST)**]…Lost employment?	☐	☐	☐
24. [VN: **(STIGDISC)**]…Felt legal or police discrimination?	☐	☐	☐

To what extent do you agree or disagree with the following statements? Would you say you disagree strongly, disagree, agree, or agree strongly to the following statements…	Disagree strongly =1	Disagree =2	Agree =3	Agree strongly =4	Not Applicable =98	No Answer = 99
25. [VN: **(SSCONNEC)**] I feel strongly connected to other men who have sex with men	☐	☐	☐	☐	☐	☐
26. [VN: **(SSCOUNT)**] In general, I feel that I can count on members of the gay community if I need help or advice	☐	☐	☐	☐	☐	☐
27. [VN: **(SSCOMMUN)**] Where I live there is no gay community that I can rely on	☐	☐	☐	☐	☐	☐

Resilience and Coping Self-Efficacy

	Week 13/ 3.0	Week 26/ 4.0	Week 39/ 5.0	Week 52/ 6.0
Relationship Status and Demographic Background	X	X	X	X
Sexual and Gender Identity, and Social Support		X		X
**Resilience and Coping Self-Efficacy**		**X**		
HIV Prevention	X	X	X	X
Sexual Behavior Assessment	X	X	X	X
HIV Risk Perception	X		X	
HIV/STI-Related Factors		X		X
HIV-Related Care and Treatment and HIV Stigma	X	X	X	X
Interest in HIV Prevention Strategies		X		
Alcohol and Drugs		X		X
Mental Health	X	X	X	X
Experience of Study Participation	X	X	X	X

SHOW RCBOUNCE – RCNEWFRIEND IF VISIT = 4.0 (WEEK 26)

To what extent do you agree or disagree with the following statements?	Disagree strongly=1	Disagree=2	Neutral=3	Agree=4	Agree strongly=5	No answer = 99
28. [VN: **RCBOUNCE**] I tend to bounce back quickly after hard times	☐	☐	☐	☐	☐	☐
29. [VN: **RCSTRESS**] I have a hard time making it through stressful events	☐	☐	☐	☐	☐	☐
30. [VN: **RERECOVER**] It does not take me long to recover from a stressful event	☐	☐	☐	☐	☐	☐
31. [VN: **RCSNAP**] It is hard for me to snap back when something bad happens	☐	☐	☐	☐	☐	☐
32. [VN: **RCDIFF**] I usually come through difficult times with little trouble	☐	☐	☐	☐	☐	☐
33. [VN: **RCSETBACKS**] I tend to take a long time to get over set-backs in my life	☐	☐	☐	☐	☐	☐

On a scale from zero to ten how certain are you that you can do the following things, when 0 means that you cannot do it at all, 5 means that you are moderately certain that you can do it, and 10 that you are certain that you can do it?

34. [VN: **RCFRIENDS**] Get friends to help you with the things you need
35. [VN: **RCCHANGE**] Sort out what can be changed, and what cannot be changed
36. [VN: **RCTHOUGHTS**] Make unpleasant thoughts go away
37. [VN: **RCACTION**] Make a plan of action and follow it when confronted with a problem
38. [VN: **RCOPTIONS**] Leave options open when things get stressful
39. [VN: **RCOFFTHOUGHTS**] Take your mind off unpleasant thoughts
40. [VN: **RCPROBLEM**] Think about one part of the problem at a time
41. [VN: **RCSOLUTION**] Find solutions to your most difficult problems
42. [VN: **RCUPSET**] Stop yourself from being upset by unpleasant thoughts
43. [VN: **RCSUPPORT**] Get emotional support from friends and family
44. [VN: **RCSAD**] Keep from feeling sad
45. [VN: **RCNEWFRIEND**] Make new friends

HIV Prevention

	Week 13/ 3.0	Week 26/ 4.0	Week 39/ 5.0	Week 52/ 6.0
Relationship Status and Demographic Background	X	X	X	X
Sexual and Gender Identity, and Social Support		X		X
Resilience and Coping Self-Efficacy		X		
**HIV Prevention**	**X**	**X**	**X**	**X**
Sexual Behavior Assessment	X	X	X	X
HIV Risk Perception	X		X	
HIV/STI-Related Factors		X		X
HIV-Related Care and Treatment and HIV Stigma	X	X	X	X
Interest in HIV Prevention Strategies		X		
Alcohol and Drugs		X		X
Mental Health	X	X	X	X
Experience of Study Participation	X	X	X	X

SHOW HIVPCOMM – HIVPINFO IF VISIT = 3.0 (WEEK 13), 4.0 (WEEK 26), 5.0 (WEEK 39) OR 6.0 (WEEK 52)

VN: **HIVPCOMM**

46. As far as you know, has there been any community activity organized by the study since your last visit?
    - 1 ☐ Yes
    - 2 ☐ No → **GO TO HIVPINFO**
    - 99 ☐ No answer → **GO TO HIVPINFO**

SHOW IF HIVPCOMM = 1 (YES)

VN: **HIVPACTIVITY**

47. Did you participate in that activity?
    - 1 ☐ Yes
    - 2 ☐ No
    - 99 ☐ No answer

VN: **HIVPINFO**

48. Since your last visit, have you seen or received any other information about HIV transmission and HIV?
    - 1 ☐ Yes
    - 2 ☐ No
    - 99 ☐ No answer

SHOW HIVPNEGTEST – HIVPSYMPTOMS IF VISIT = 3.0 (WEEK 13)

The following statements are about what happens in the first weeks after someone is infected with HIV. Can you say whether you think the following statements are true or false? You can also say that you don't know the answer.

	True	False	Do not know	No answer
49. [VN: **HIVPNEGTEST**] Someone who was recently infected with HIV may have a negative HIV test. This is called the "window period"	☐	☐	☐	☐
50. [VN: **HIVPNOSYMP**] Someone who recently is infected with HIV never has any symptoms	☐	☐	☐	☐
51. [VN: **HIVPSPREAD**] Someone recently infected with HIV can spread HIV even if he has a negative test	☐	☐	☐	☐
52. [VN: **HIVPSEXUAL**] Someone who was recently infected with HIV is at particularly high risk of transmitting HIV to a sexual partner	☐	☐	☐	☐

VN: **HIVPSYMPTOMS**

53. Most people will have some symptoms in the first 1-4 weeks after getting infected with HIV. Do you know any symptoms people might have when they just were infected with HIV? *Check all that are mentioned.*
    HIVPSYMPTOMS_1 ☐ Fever
    HIVPSYMPTOMS _2☐ Swollen glands
    HIVPSYMPTOMS _3☐ Sore throat
    HIVPSYMPTOMS _4☐ Rash
    HIVPSYMPTOMS _5☐ Fatigue
    HIVPSYMPTOMS _6☐ Muscle and join aches and pains
    HIVPSYMPTOMS _7☐ Headache
    HIVPSYMPTOMS _98☐ Don't know any symptoms
    HIVPSYMPTOMS _99☐ No answer

Sexual Behavior Assessment

	Week 13/ 3.0	Week 26/ 4.0	Week 39/ 5.0	Week 52/ 6.0
Relationship Status	X		X	
Demographic Background		X		X
Sexual and Gender Identity, and Social Support		X		X
Resilience and Coping Self-Efficacy		X		
HIV Prevention	X	X	X	X
**Sexual Behavior Assessment**	**X**	**X**	**X**	**X**
HIV Risk Perception	X		X	
HIV/STI-Related Factors		X		X
HIV-Related Care and Treatment	X		X	
HIV-Related Care and Treatment and HIV Stigma		X		X
Interest in HIV Prevention Strategies		X		
Alcohol and Drugs		X		X
Mental Health	X	X	X	X
Experience of Study Participation	X	X	X	X

SHOW (SPHMANY) – (SPFEMIW) IF VISIT = 3.0 (WEEK 13), 4.0 (WEEK 26), 5.0 (WEEK 39) OR 6.0 (WEEK 52)

Assessment steps
1. Elicit number of sexual partners and nicknames (SPHMANY – SPRP3).
2. Determine sexual frequency and timing (SP1TIMES – SP1RECENT).
3. Assess personal and relational characteristics and sexual behavior partner by partner (SP1GENDER – SP1TSTUDY).
4. If 4 or more sex partners: assess additional sexual risk (SP4UNPSEX – SPFEMIW).

This next section will be about persons you have had sex with since your previous visit. With “sex” we mean “anal sex” when we talk about male partners and vaginal or anal sex when we talk about female partners.

In order to do this, we will ask you to provide nicknames. We will then use these nicknames to customize the questions so that they are specific to the persons you have had sex with.

It is important that you choose a nickname that will best help you remember the person. The names you provide are meant to help you only and should not reveal your partner’s full identity. We do not want to know who your partners actually are.

Some examples of nicknames that you might choose are: a person’s first name, a nickname you call the partner by, or the place where you both met. You could also use a description of what a person is to you, such as “lover” or “buddy.”

VN: **(SPHMANY WITH MINOR WORDING CHANGE)**

54. How many persons have you had sex with since your last visit? This could be a steady partner, persons that you had sex with only once, that gave you something in return for sex, or that you are no longer in touch with.

_________________ partners
99☐ No answer

IF (SPHMANY) = 0, SKIP TO INTRODUCTION OF NEXT APPLICABLE SECTION [HIV RISK PERCEPTION IF VISIT 3.0 (WEEK 13) OR VISIT 5.0 (WEEK 39); HIV/STI-RELATED FACTORS IF VISIT 4.0 (WEEK 26) OR VISIT 6.0 (WEEK 52)].

SHOW IF (SPHMANY) = 1, 2 OR 3

IF SPHMANY = 1: I would now like to ask you some questions about the person you have had sex with since your previous visit.

IF SPHMANY >1: I would now like to ask you some questions about each of these partners you have had sex with since your previous visit.

55. **IF SPHMANY = 1:** Could you give me initials or a nickname for this person?

IF SPHMANY > 1: Could you give me initials or a nickname for each of these partners?

SHOW IF (SPHMANY) = 1
VN: **(SP1)**
Sexual partner 1: _________ **[OPEN TEXT]**

SHOW IF (SPHMANY) >1
VN: **(SP2)**
Sexual Partner 2: ________ **[OPEN TEXT]**

SHOW IF (SPHMANY) >2
VN: **(SP3)**
Sexual Partner 3: _________ **[OPEN TEXT]**

If (SPHMANY) = 1, 2 or 3 partners, skip to "loop of questions to ask each sexual partner" starting with (SP1TIMES). If > 3 partners, continue with next set of questions to get nicknames of 3 partners only.

SHOW IF (SPHMANY) >3 (FOUR OR MORE PARTNERS)

I would now like to ask you some questions about the persons you have had sex with since your previous visit. Because it would take too long to discuss them all, I would like to select three of them. There are different types of sexual partners. I am going to ask a few questions to help you select three different types of sexual partners you've had sex with since your previous visit.

VN: **(SPMAIN WITH MINOR WORDING CHANGE)**

56. How many of the people you had sex with since your previous visit do you consider to be a main partner? A main partner is someone you have a relationship with, like a lover, boyfriend or girlfriend, or a spouse.
   1 ☐ None → **GO TO (SPCASUAL)**
   2 ☐ One
   3 ☐ More than one

SHOW IF (SPMAIN) > 1
VN: **(SPMP)**

57. **IF (SPMAIN) = 2:** Please give me initials or a nickname for this person.
    IF (SPMAIN) = 3: Think about the most important one and please give me initials or a nickname for this person.

    ______ **[OPEN TEXT]**

VN: **(SPCASUAL WITH MINOR WORDING CHANGES)**

58. (Not including anyone we may have already discussed), how many of the people you had sex with since your previous visit do you consider to be a casual partner? A casual partner is someone with whom you just have sex once or twice, or that you hook up with from time to time to have sex.

1☐None→ **GO TO (SPEXCHAN)**
2☐One
3☐More than one

SHOW IF (SPCASUAL) > 1
VN: **(SPCP)**

59. **IF (SPCASUAL) = 2:** Please give me initials or a nickname for this person.
IF (SPCASUAL) = 3: Think about the most recent one and please give me initials or a nickname for this person.

_______ **[OPEN TEXT]**

VN: **(SPEXCHAN)**

60. (Not including anyone we may have already discussed), are any of these persons an exchange partner? An exchange partner is someone you have sex with in exchange for food, money, shelter or drugs.

1☐None→ **GO TO (SPWOMAN)**
2☐One
3☐More than one

SHOW IF (SPEXCHAN) > 1
VN: **(SPEP)**

61. **IF (SPEXCHAN) = 2:** Please give me initials or a nickname for this person.
IF (SPEXCHAN) = 3: Think about the most recent one and please give me initials or a nickname for this person.

_______ **[OPEN TEXT]**

**SHOW IF 3 PARTNERS HAVE NOT ALREADY BEEN SELECTED (IF ANY 1 OF (SPMP), (SPCP), OR (SPEP) = 1 (NONE))**

VN: **(SPWOMAN WITH MINOR WORDING CHANGES)**

62. (Not including anyone we may have already discussed), are any of the people you've had sex with since your previous visit a (biological) woman?

1 ☐ None
2 ☐ One
3 ☐ More than One

SHOW IF (SPWOMAN) >1
VN: **(SPWP)**

63. **IF (SPWOMAN) = 2:** Please give me initials or a nickname for this person.
IF (SPWOMAN) = 3: Think about the most recent one not already chosen and please give me initials or a nickname for this person.

_______ **[OPEN TEXT]**

SHOW IF 3 PARTNERS STILL HAVE NOT BEEN SELECTED (IF ANY 2 OF (SPMAIN), (SPCASUAL), (SPEXCHAN) OR (SPWOMAN) = 1 (NONE))

VN: **(SPRP1)**

64. Not including anyone we may have already discussed, think about your next most recent sexual partner since your last visit.

Please give me initials or a nickname for this person. _______ **[OPEN TEXT]**

SHOW IF 3 PARTNERS STILL HAVE NOT BEEN SELECTED (IF ANY 3 OF (SPMAIN), (SPCASUAL), (SPEXCHAN), (SPWOMAN) OR (SPRP1) = 1 (NONE))

VN: **(SPRP2)**

65. Not including anyone we may have already discussed, think about your next most recent sexual partner since your last visit

Please give me initials or a nickname for this person. _______ **[OPEN TEXT]**

SHOW IF 3 PARTNERS STILL HAVE NOT BEEN SELECTED (IF (SPMAIN), (SPCASUAL), (SPEXCHAN) AND (SPWOMAN) ALL = 1 (NONE) AND (SPRP1) AND (SPRP2) HAVE ANSWERS.

VN: **(SPRP3)**

66. Not including anyone we may have already discussed, think about your next most recent sexual partner since your last visit.

Please give me initials or a nickname for this person. _______ **[OPEN TEXT]**

LOOP OF QUESTIONS TO ASK FOR EACH SEXUAL PARTNER

SHOW ONLY FOR SP2 (2ND PARTNER MENTIONED) AND SP3 (3RD PARTNER MENTIONED): We will now discuss your next partner.

SHOW ALL

VN: **(SP1TIMES WITH MINOR WORDING CHANGE)**

67. Since your previous visit, have you had sex with [XXX] once or more often?
    - 1 ☐ One time
    - 2 ☐ More than once

SHOW IF (SP1TIMES) = 1

VN: **(SP1DATE WITH MINOR WORDING CHANGE)**

68. When did you have sex with [XXX]? If you don't know exactly please give your best guess.
    - ______MM/YYYY
    - 2 ☐Unknown
    - 99☐ No answer

PROMPT: IF MM/YYYY > today's date: "Your response cannot be a date in the future. Please correct your response. "

SHOW IF (SP1TIMES) = 2
VN: **(SP1FIRST)**

69. When was the first time you had sex with [XXX]? Please provide the month and year. If you don't know exactly, please give your best guess. (*You may enter 99 for the month if the participant does not want to guess*.)

_____MM/YYYY **(Allow 99 for MM)**
2 ☐Unknown
99☐ No answer

PROMPT: If MM/YYYY is > today's date: "Your response cannot be a date in the future. Please correct your response. "

SHOW IF (SP1TIMES) = 2
VN: **(SP1RECENT)**

70. When was the most recent time you had sex with [XXX]?

_____MM/YYYY
2 ☐Unknown
99☐ No answer

PROMPT: If MM/YYYY > today's date: "Your response cannot be a date in the future. Please correct your response."

PROMPT: If (SP1RECENT) is < (SP1FIRST): "The most recent time you had sex with your partner has to be on or after the date you first had sex with that partner. Please correct your response."

SHOW ALL
VN: **SP1PREV**

71. Is [XXX] one of the partners that you discussed at your previous study visit?
    1 ☐ Yes
    2 ☐ No
    99 ☐ No answer

VN: **(SP1GENDER)**

72. Is [XXX] male, female, or transgender?
    1☐ Male
    2☐ Female
    3☐ Transgender: male to female
    4☐ Transgender: female to male
    5☐ Intersex
    99☐ No answer

IF PARTNER IS SAME AS DISCUSSED AT PREVIOUS VISIT, (SP1WHERE) – (SP1STATAS) SHOULD BE SKIPPED.

SHOW (SP1WHERE) - (SP1STATAS) IF SP1PREV = 2 OR 99. OTHERWISE, GO TO (SP1PLACE).
VN: **(SP1WHERE)**

73. Where did you first meet [XXX]?
    1☐ Through friends
    2☐ In a bar/club/dancing
    3☐ School or work
    4☐ At church
    5☐ At social event
    6☐ Via the internet/social media
    7☐ Cruising area (park/public ground/bushes, public toilet, railway/bus station)
    8☐ On the street
    9☐ Other
    99☐ No answer

VN: **(SP1FMEET)**

74. Do you remember the month and year when you met [XXX] for the first time? If not, give your best guess. (*You may enter 99 for the month if the participant does not want to guess*.)

____MM/YYYY **(Allow 99 for MM)**

99☐ No answer

PROMPT: If (SP1FMEET) MM/YYYY is after the current MM/YYYY: "Your response cannot be a date in the future. Please correct your response."

VN: **(SP1AGE)**

75. What is [XXX]'s current age (if you are unsure of the exact age, choose an age that you think is close)?

1 ☐ ______________years → **GO TO (SP1RACE)**

2 ☐ Don't know

99☐ No answer → **GO TO (SP1RACE)**

SHOW IF (SP1AGE) = 2

VN: **(SP1OLDER)**

76. Compared to you, is [XXX] much older, somewhat older, about the same age, or somewhat or much younger?

1☐ Much older

2☐ Somewhat older

3☐ (More or less) same age

4☐ Somewhat younger

5☐ Much younger

99☐ No answer

VN: **(SP1RACE)**

77. What is the race or ethnicity of [XXX]?

Show If Site is Soweto or Cape Town:

1=Black

2=White

3= Indian/Asian

4= Coloured

5=Other

99=No answer

Show If Site is Malawi:

2= White

6=Chewa

7= Ngoni

8= Yao

9= Tumbuka

5= Other

99=No answer

Show if Site is Kenya

10=Luo,

11= Kisii

12=Luhya

13=Kalenjin

2=White

3= Indian/Asian

5=Other

99=No answer

SHOW SP1ATTRACT – SP1MASCU IF (SP1GENDER) = 1 (MALE)

VN: **(SP1ATTRACT)**

78. As far as you know, is [XXX] sexually attracted to men or to women?
    - 1☐ Only to women
    - 2☐ More to women than to men
    - 3☐ To women and men equally
    - 4☐ More to men than to women
    - 5☐ Only to men
    - 6☐ Don't know
    - 99☐ No answer

VN: **(SP1FARLIVE)**

79. How far do you live from [XXX]?
    - 1☐ Same household
    - 2☐ Same area of town
    - 3☐ Another area of town
    - 4☐ Outside of the city
    - 5☐ Outside of the country
    - 6☐ Don't know
    - 7☐ Person listed is homeless and is not living anywhere regularly
    - 99☐ No answer

VN: **(SP1MONEY)**

80. Who would you say has generally more money to spend, [XXX] or you?
    - 1☐ Partner
    - 2☐ Participant self
    - 3☐ The same
    - 4☐ Don't know
    - 99☐ No answer

VN: **(SP1MASCU)**

81. Would you say that [XXX] is more or less masculine than you?
    - 1☐ Partner more masculine
    - 2☐ Partner less masculine
    - 3☐ The same
    - 99☐ No answer

SHOW ALL

VN: **(SP1KNOWN)**

82. How well did you know [XXX] prior to having sex with him/her for the first time?
    - 1☐ Not at all (just met)
    - 2☐ Somewhat
    - 3☐ Fairly well
    - 4☐ Very well
    - 99☐ No answer

SHOW IF (SP1TIMES) = 2

VN: **(SP1CLOSE)**

83. How do you feel about your relationship with [XXX]? Would you say your relationship is:
    - 1☐ Very close
    - 2☐ Somewhat close
    - 3☐ Not at all close
    - 99☐ No answer

SHOW ALL

VN: **(SP1SHARE)**

84. **IF SP1TIMES = 1:** Did [XXX] share his/her HIV status with you before you had sex?

IF SP1TIMES = 2: Did [XXX] share his/her HIV status with you before you first had sex?

1☐ Yes
2☐ No → **GO TO (SP1STATBFS)**
3☐ Don't know → **GO TO (SP1STATBFS)**
99☐ No answer → **GO TO (SP1STATBFS)**

SHOW IF (SP1SHARE) = 1

VN: **(SP1STATBS)**

85. What was [XXX]'s status at that time?

1☐ HIV-negative
2☐ HIV-positive
3☐ Don't know
99☐ No answer

SHOW ALL

VN: **(SP1STATBFS)**

86. **IF SP1TIMES = 1:** Did you share your HIV status with him/her before you had sex?

IF SP1TIMES = 2: Did you share your HIV status with him/her before you first had sex?

1☐ Yes
2☐ No
3☐ Don't know
99☐ No answer

SHOW IF (SP1TIMES) = 2 AND (SP1SHARE) = 2 OR 3

VN: **(SP1SHARAS)**

87. Did you and [XXX] share both of your HIV statuses any time after you first had sex?

1☐ Yes
2☐ No → **GO TO (SP1PLACE)**
3☐ Don't know → **GO TO (SP1PLACE)**
99☐ No answer

SHOW IF (SP1SHARAS) =1

VN: **(SP1STATAS)**

88. What was [XXX]'s status at that time?

1☐ HIV-negative
2☐ HIV-positive
3☐ Don't know
99☐ No answer

SHOW ALL

VN: **(SP1PLACE)**

89. Where did you have sex with [XXX]? You can select more than one response.

(VN: **SP1PLACE_1**)☐ At participant's place
(VN: **SP1PLACE_2**)☐ At partner's place
(VN: **SP1PLACE_3**) ☐ At the place we live together
(VN: **SP1PLACE_4**)☐ Elsewhere
(VN: **SP1PLACE_99**) ☐ No answer

SPECIFIC QUESTIONS (PART OF LOOP OF QUESTIONS) FOR A MALE SEX PARTNER

SHOW IF (SP2GENDER) =1, 3, OR 5 (MALE)

VN: **(SP1ANAL WITH MINOR WORDING CHANGE)**

90. Did you have receptive anal sex with [XXX], meaning you were the “bottom”, since your previous visit?
    1☐ Yes
    2☐ No → **GO TO (SP1INSERT)**
    99☐ No answer → **GO TO (SP1INSERT)**

SHOW IF (SP1ANAL) = 1 AND (SP1TIMES) = 1
VN: **(SP1ASRASC)**

91. Did [XXX] use a condom when you had receptive anal sex with him?
    1☐ He used condom
    2☐ He did not use condom → **GO TO (SP1INSERT)**
    3☐ Cannot remember → **GO TO (SP1INSERT)**
    99☐ No answer → **GO TO (SP1INSERT)**

SHOW IF (SP1ASRASC) =1
VN: **(SP1RASCT)**

92. Did [XXX] wear the condom all the time or part of the time while he was inside you?
    1☐ All the time
    2☐ Part of the time
    3☐ Cannot remember
    99☐ No answer

SHOW IF (SP1ANAL) = 1 AND (SP1TIMES) = 2
VN: **(SP1ANALNO WITH MINOR WORDING CHANGE)**

93. How many times have you had receptive anal sex with [XXX] since your previous visit? If you don’t know exactly, give us your best guess.

    ________ times
    99☐ No answer

PROMPT: If (SP1ANAL) = 1, (SP1ANALNO) must be > 0: “Your response must be greater than 0. Please correct your response.”

SHOW IF (SP1ANAL) = 1 AND (SP1TIMES) = 2
VN: **(SP1UNPROT)**

94. Most men do not use condoms all the time they have anal sex. Of these **{R: (SP1ANALNO)}** times that you had receptive anal sex with [XXX], how many were unprotected, that means that no condom was used? If you don’t know exactly, give us your best guess.

    ________ times [*MAX= (SP1ANALNO)*]
    99☐ No answer

PROMPT: If number of times entered is > value of (SP1ANALNO): “Your response must be less than or equal to the total number of times you had receptive anal sex with this partner.”

SHOW IF (SP1TIMES) = 2 & (SP1UNPROT) < (SP1ANALNO)
VN: **(SP1ANCON)**

95. Sometimes men only put on a condom after they have already had their penis inside the other person's anus. Since your last visit, how many times did [XXX] only use condoms part of the time while he was inside you? If you don't know exactly, give us your best guess.

________ times
99☐ No answer

SHOW IF (SP1TIMES) = 1 OR 2
VN: **(<u>SP1INSERT</u> WITH MINOR WORDING CHANGES)**

96. Did you have insertive anal sex with [XXX], meaning you were the "top", since your previous visit?
1☐ Yes
2☐ No → **GO TO LOGIC BEFORE (SP1GOTCON), IF APPLICABLE; OTHERWISE GO TO (SP1AODOTH)**
99☐ No answer

SHOW IF (SP1INSERT) = 1 & (SP1TIMES) = 1
VN: **(SP1ASIASC)**

97. Did you use a condom when you had insertive anal sex with him?
1☐ I used condom
2☐ I did not use condom
3☐ Cannot remember
99☐ No answer

SHOW IF (SP1ASIASC) = 1 (USED CONDOM) AND (SP1TIMES) = 1 (SEX ONLY ONCE)
VN: **(SP1IASCT)**

98. Did you wear the condom all the time or part of the time while you were inside of [XXX]?
1☐ All the time
2☐ Part of the time
3☐ Cannot remember
99☐ No answer

SHOW IF (SP1INSERT) = 1 AND (SP1TIMES) =2
VN: **(<u>SP1INTIMES</u> WITH MINOR WORDING CHANGE)**

99. How many times have you had insertive anal sex with [XXX] since your previous visit? If you don't know exactly, give us your best guess.

________ times
99☐ No answer

PROMPT: If (SP1INSERT) = 1, (SP1INTIMES) must be > 0: "Your response must be greater than 0. Please correct your response."

SHOW IF (SP1INSERT) = 1 AND (SP1TIMES) = 2
VN: **(SP1INPROT)**

100. Most men do not use condoms all the time they have anal sex. Of these **{R: (SP1INTIMES)}** times that you had insertive anal sex with [XXX], how many were <u>unprotected</u>, that means that <u>no</u> condom was used? If you don't know exactly, give us your best guess.

________ times (*MAX = (SP1INTIMES)*)
99☐ No answer

PROMPT: If number of times entered is > (SP1INTIMES): "Your response must be less than or equal to the total number of times you had insertive anal sex with this partner."

SHOW IF (SP1TIMES) = 2 AND [(SP1INPROT) < (SP1INTIMES)] (IF CONDOMS HAD BEEN USED IN INSERTIVE SEX)

VN: **(SP1INCON)**

101. Sometimes men only put on a condom after they have already been with their penis inside the other person's anus. Since your previous visit, how many times did you only use condoms part of the time while you were inside [XXX]? If you don't know exactly, give us your best guess.

_________ times

99☐ No answer

NOTE: AFTER THESE QUESTIONS FOR MALE SEX PARTNER, CONTINUE WITH LOGIC ABOVE (SP1GOTCON).

SPECIFIC QUESTIONS (PART OF LOOP OF QUESTIONS) FOR A FEMALE SEX PARTNER

SHOW IF (SP1GENDER) =2, 4 (FEMALE)

VN: **(SP1VAGIN WITH MINOR WORDING CHANGE)**

102. Did you have vaginal intercourse with [XXX] since your previous visit?

1☐ Yes

2☐ No → **GO TO (SP1FEMAN)**

99☐ No answer

SHOW IF (SP1VAGIN) = 1 AND (SP1TIMES) = 1

VN: **(SP1VAGCON)**

103. Did you use a condom when you had vaginal intercourse with [XXX]?

1☐ I used condom

2☐ I did not use condom

3☐ Cannot remember

99☐ No answer

SHOW IF (SP1VAGCON) = 1 AND (SP1TIMES) = 1

VN: **(SP1VCTIM)**

104. Did you wear the condom all the time or part of the time when you were inside [XXX]?

1☐ All the time

2☐ Part of the time

3☐ Cannot remember

99☐ No answer

SHOW IF (SP1VAGIN) = 1 AND (SP1TIMES) = 2

VN: **(SP1VTIMES)**

105. How many times have you had vaginal intercourse with [XXX] since your previous visit?

_________ times

99☐ No answer

PROMPT: If (SP1VAGIN) = 1, (SP1VTIMES) must be > 0: "Your response must be greater than 0. Please correct your response."

SHOW IF (SP1VAGIN) = 1 AND (SP1TIMES) = 2

VN: **(SP1VPROT)**

106. Of these **{R: (SP1VTIMES)}** times that you had vaginal intercourse with [XXX], how many were unprotected, that means that no condom was used?

________ times [*MAX = (SP1VTIMES)*]

99☐ No answer

PROMPT: If number of times entered is > (SP1VTIMES): "This response must be less than or equal to the total number of times you had vaginal intercourse with this partner."

SHOW IF (SP1VAGIN) = 1 AND (SP1TIMES) = 2 AND [(SP1VTIMES) > (SP1VPROT)] (IF CONDOMS HAD BEEN USED IN VAGINAL SEX)

VN: **(SP1VAGCPT)**

107. Sometimes men only put on a condom after they have already been with their penis inside the woman's vagina. How many times did you only use condoms part of the time while you were inside [XXX]?

________ times

99☐ No answer

SHOW IF (SP1TIMES) = 1 OR 2

VN: **(SP1FEMAN)**

108. Did you have anal sex with [XXX] since your previous visit?

1☐ Yes

2☐ No → **GO TO LOGIC BEFORE (SP1GOTCON), IF APPLICABLE; OTHERWISE GO TO (SP1AODOTH)**

99☐ No answer

SHOW IF (SP1FEMAN) = 1 AND (SP1TIMES) = 1

VN: **(SP1FEMAC)**

109. Did you use a condom when you had anal sex with [XXX]?

1☐ I used condom

2☐ I did not use condom

3☐ Cannot remember

99☐ No answer

SHOW IF (SP1FEMAC) = 1 AND (SP1TIMES) = 1

VN: **(SP1FASCT)**

110. Did you wear the condom all the time or part of the time while you were inside of [XXX]?

1☐ All the time

2☐ Part of the time

3☐ Cannot remember

99☐ No answer

SHOW IF (SP1FEMAN) = 1 AND (SP1TIMES) = 2

VN: **(SP1FATIMES WITH MINOR WORDING CHANGE)**

111. How many times have you had anal sex with [XXX] since your previous visit?

________ times

99☐ No answer

PROMPT: If (SP1FEMAN) = 1, (SP1FATIMES) must be > 0: "Your response must be greater than 0. Please correct your response"

SHOW IF (SP1FEMAN) = 1 AND (SP1TIMES) = 2
VN: **(SP1FAPROT)**

112. Most men do not use condoms all the time they have sex with a woman. Of these **{R: (SP1FATIMES)}** times that you had anal sex with [XXX], how many were unprotected, that means that no condom was used?

_________ times [*MAX = (SP1FATIMES*)]
99☐ No answer

PROMPT: If number of times entered is > (SP1FATIMES): "This response must be less than or equal to the total number of times you had anal sex with this partner."

SHOW IF (SP1FEMAN) = 1 AND (SP1TIMES) = 2 AND ((SP1FATIMES) > (SP1FAPROT)) (IF CONDOMS HAD BEEN USED IN ANAL SEX WITH FEMALE PARTNER)
VN: **(SP1FACON)**

113. Sometimes men only put on a condom after they have already been with their penis inside the woman's anus. Since your previous visit, how many times did you only use condoms part of the time while you were inside [XXX]?

_________ times
99☐ No answer

NOTE: AFTER THESE SEX QUESTIONS FOR FEMALE PARTNERS, CONTINUE WITH LOGIC ABOVE (SP1GOTCON).

SHOW TO MALE OR FEMALE PARTNERS IF CONDOMS HAVE BEEN USED OR PARTICIPANT IS UNSURE IF CONDOMS WERE USED, DEFINED AS:

FOR (SP1TIMES) = 2: CONDOMS WERE USED IF (SP1UNPROT) < (SP1ANALNO), OR (SP1INPROT) < (SP1INTIMES), OR (SP1VPROT) < (SP1VTIMES), OR (SP1FAPROT) < (SP1FATIMES)

FOR (SP1TIMES) = 1: CONDOMS WERE USED IF (SP1ASRASC) = 1 OR (SP1ASIASC) = 1 OR (SP1VAGCON) = 1 OR (SP1FEMAC) = 1

Now I'm going to ask a few questions about your use of condoms since your previous visit.

VN: **(SP1GOTCON)**

114. **IF SP1TIMES = 1:** Who provided the condom?
IF SP1TIMES = 2: Who took care that condoms were available?
1☐ Participant
2☐ Sexual partner ([XXX])
3☐ Both
99☐ No answer

VN: **(SP1WANTCON)**

115. Were condoms used because [XXX] or because you wanted to use them?
1☐ Participant wanted to use them
2☐ Partner wanted to use them
3☐ Both wanted to use them
99☐ No answer

SHOW IF (SP1TIMES) = 2
VN: **(SP1CSLPT)**

116. When you and [XXX] used condoms, how many times did the condom slip off?

_________ times
99☐ No answer

SHOW IF (SP1TIMES) = 2
VN: **(SP1CBRKT)**

117. When you and [XXX] used condoms, how many times did the condom break?

_________ times
99☐ No answer

SHOW IF (SP1TIMES) = 1
VN: **(SP1CSLIP)**

118. When you and [XXX] used condoms, did the condom slip off?

1☐ Condom slipped of
2☐ Condom did not slip off
3☐ Don't remember
99☐ No answer

SHOW IF (SP1TIMES) = 1
VN: **(SP1CBREK)**

119. When you and [XXX] used condoms, did the condom break?

1☐ Condom did break
2☐ Condom did not break
3☐ Don't remember
99☐ No answer

SHOW TO MALE OR FEMALE PARTNERS IF CONDOMS HAD NOT BEEN USED OR PARTICIPANT WAS UNSURE, DEFINED AS:

FOR (SP1TIMES) = 2: CONDOMS WERE NOT USED IF (SP1UNPROT) = (SP1ANALNO) (RECEPTIVE ANAL SEX MALE PARTNER) OR (SP1INPROT) = (SP1INTIMES) (INSERTIVE ANAL SEX MALE PARTNER) OR (SP1VPROT) = (SP1VTIMES) (VAGINAL SEX FEMALE PARTNER) OR (SP1FAPROT) = (SP1FATIMES) (ANAL SEX FEMALE PARTNER)

FOR (SP1TIMES) = 1: CONDOMS WERE NOT USED IF (SP1ASRASC) = 2, 3, 99 (RECEPTIVE ANAL SEX MALE PARTNER) OR (SP1ASIASC) = 2, 3, 99 (INSERTIVE ANAL SEX MALE PARTNER) OR (SP1VAGCON) = 2, 3, 99 (VAGINAL SEX FEMALE PARTNER) OR (SP1FEMAC) = 2, 3, 99 (ANAL SEX FEMALE PARTNER)

VN: **(SP1NOTUSE)**

120. What was the main reason why condoms were not (always) used when you had sex with [XXX]?

1☐ Condom wasn't available
2☐ Condom was available, but I didn't want to use one
3☐ Condom was available, but [XXX] didn't want to use one
4☐ Both same HIV status
5☐ Other reason
99☐ No answer

FOR ALL PARTNERS (MALE OR FEMALE) SHOW IF (SP1ANAL) = 1 OR (SP1INSERT) = 1 OR (SP1FEMAN) = 1 (IF PARTICIPANT HAD ANAL SEX WITH MALE OR FEMALE PARTNER)

TOP OF PAGE: Since your previous visit…

SHOW IF SP1TIMES = 1

VN: **(SP1LUBE1)**

121. Was lubricant used when [XXX] and you had anal sex?

1☐ Yes
2☐ No
3☐ Don't remember
99☐ No answer → **GO TO (SP1AODOTH)**

SHOW IF SP1TIMES = 2

VN: **(SP1LUBE2)**

122. How often was lubricant used when [XXX] and you had anal sex? Would you say…

1☐ Never
2☐ Rarely
3☐ Sometimes
4☐ Most of the times
5☐ Always
99☐ No answer → **GO TO (SP1AODOTH)**

INSERT PAGE BREAK

AT TOP OF PAGE: Since your previous visit…

SHOW IF (SP1LUBE) = 1 OR (SP1LUBE2) > 1 ((SP1TIMES) CAN = 1 OR 2)

VN: **(SP1LUBTYPE)**

123. What type of lubricant did you use with [XXX]? *(Mark all that apply)*

SP1LUBTYPE_1 ☐ Vaseline
SP1LUBTYPE_2 ☐ Commercially or clinic-sourced product (e.g., KY Jelly)
SP1LUBTYPE_3 ☐ Body lotion or baby oil
SP1LUBTYPE_4 ☐ Vegetable or food oil
SP1LUBTYPE_5 ☐ Butter or margarine
SP1LUBTYPE_6 ☐ Soap
SP1LUBTYPE_7 ☐ Grease
SP1LUBTYPE_8 ☐ Saliva or water
SP1LUBTYPE_9 ☐ Other
SP1LUBTYPE_99 ☐ No answer

INSERT PAGE BREAK

SHOW IF (SP1TIMES) = 1 OR 2

AT TOP OF PAGE: Since your previous visit…

VN: **(SP1AODOTH)**

124. **IF SP1TIMES = 1:** Was [XXX] under the influence of alcohol or drugs when you and [XXX] had sex? Would you say…
IF SP1TIMES = 2: How often was [XXX] under the influence of alcohol or drugs when you and [XXX] had sex? Would you say…
1☐ Never
2☐ Rarely
3☐ Sometimes
4☐ Most of the time
5☐ Always
6☐ No
7☐ Yes
99☐ No answer

SHOW "No", "Yes" and "No answer" IF (SP1TIMES) = 1
SHOW "Never", "Rarely", "Sometimes", "Most of the time", "Always" and "No answer" "IF (SP1TIMES) = 2

VN: **(SP1AODSELF)**

125. **IF SP1TIMES = 1:** Were you under the influence of alcohol or drugs when you and [XXX] had sex?
IF SP1TIMES = 2: How often were you under the influence of alcohol or drugs when you and [XXX] had sex?
1☐ Never
2☐ Rarely
3☐ Sometimes
4☐ Most of the time
5☐ Always
6☐ No
7☐ Yes
99☐ No answer

SHOW "No", "Yes" and "No answer" IF (SP1TIMES) = 1
SHOW "Never", "Rarely", "Sometimes", "Most of the time", "Always" and "No answer" "IF (SP1TIMES) = 2

TOP OF PAGE: Since your last visit…

VN: **(SP1PAYYOU)**

126. **IF SP1TIMES = 1:** Did [XXX] pay you for having sex with him/her?
IF SP1TIMES = 2: Did [XXX] ever pay you for having sex with him/her?
1☐ Yes
2☐ No
99☐ No answer

VN: **(SP1GETELSE)**

127. **IF SP1TIMES = 1:** Did you get anything else from [XXX] for having sex with him/her, such as food, a place to sleep, or clothing?
IF SP1TIMES = 2: Did you ever get anything else from [XXX] for having sex with him/her, such as food, a place to sleep, or clothing?
1☐ Yes
2☐ No
99☐ No answer

TOP OF PAGE: Since your last visit…

VN: **(SP1PAYTHEM)**

128. **IF SP1TIMES = 1:** Did you pay [XXX] for having sex with you?
IF SP1TIMES = 2: Did you ever pay [XXX] for having sex with you?
1☐ Yes
2☐ No
99☐ No answer

VN: **(SP1GIVELSE)**

129. **IF SP1TIMES = 1:** Did you give [XXX] anything for having sex with you, such as food, a place to sleep, or clothing?
IF SP1TIMES = 2: Did you ever give [XXX] anything for having sex with you, such as food, a place to sleep, or clothing?
1☐ Yes
2☐ No
99☐ No answer

VN: **(LIKE SP1FORCED WITH MINOR WORDING CHANGE)**

130. **IF SP1TIMES = 1:** Did you feel that [XXX] forced you to have sex with him/her when you did not want to yourself?
IF SP1TIMES = 2: Since your previous visit, did you ever feel that [XXX] forced you to have sex with him/her when you did not want to yourself?
1☐ Yes
2☐ No **→ GO TO LOGIC BEFORE (SP1OFTYFT)**
99☐ No answer

SHOW IF (SP1FORCED) = 1 AND (SP1TIMES) = 2
VN: **(SP1OFTYFF)**

131. How often would you say you felt forced by [XXX] to have sex?
1☐ Only once
2☐ A few times
3☐ Regularly
4☐ Often
5☐ Very often
99☐ No answer

SHOW IF (SP1TIMES) = 1
VN: **(SP1FORCY)**

132. Did you force [XXX] to have sex with you?
1☐ Yes
2☐ No
99☐ No answer

SHOW IF (SP1TIMES) = 2
VN: **(<u>SP1OFTYFT</u> WITH MINOR WORDING CHANGE)**

133. How often would you say you have forced [XXX] to have sex with <u>you</u> since your previous visit?
1☐ Never
2☐ Only once
3☐ A few times
4☐ Regularly
5☐ Often
6☐ Very often
99☐ No answer

SHOW (SP1SWW) – (SP1TSTUDY) IF (SP1GENDER) = 1 (IF PARTNER IS MALE)

VN: **(SP1SWW)**

134. As far as you know, does [XXX] also have sex with women?
   - 1☐ Yes
   - 2☐ No
   - 3☐ Don't know
   - 99☐ No answer

SHOW IF (SP1TIMES) = 2

VN: **(SP1SWO WITH MINOR WORDING CHANGE)**

135. Since your last visit, during the time you were sexually involved with [XXX], did [XXX] have sex with anyone else?
   - 1☐ Yes
   - 2☐ No
   - 3☐ Don't know
   - 99☐ No answer

SHOW IF (SP1TIMES) = 1 OR 2

VN: **(SP1AGAIN)**

136. Do you think you will have sex again with [XXX]?
   - 1☐ Yes
   - 2☐ No
   - 3☐ Don't know
   - 99☐ No answer

SHOW IF (SP1TIMES) = 2

VN: **(SP1YSTUDY)**

137. Have you ever talked with [XXX] about your participation in this research study?
   - 1☐ Yes
   - 2☐ No
   - 3☐ Don't know
   - 99☐ No answer

SHOW IF (SP1TIMES) = 1 OR 2

VN: **(SP1TSTUDY)**

138. As far as you know, does [XXX] participate in this research study?
   - 1☐ Yes
   - 2☐ No
   - 3☐Don't know
   - 99☐ No answer

GO BACK TO START OF SEXUAL PARTNER SECTION [(SP1TIMES) IS THE FIRST QUESTION] IF MORE PARTNERS HAVE TO BE DISCUSSED. OTHERWISE:

IF (SP#GENDER) = 2 (FEMALE) THEN GO TO (SPFEMIW)

IF (SP#GENDER) ≠ 2 (IS NOT FEMALE) AND NO MORE PARTNERS AND PARTICIPANT HAD 3 OR LESS PARTNERS CONTINUE WITH NEXT SECTION (NEXT SECTION DEPENDS ON THE VISIT)

IF 3 PARTNERS HAVE BEEN DISCUSSED AND PARTICIPANT HAD 4 PARTNERS CONTINUE WITH (SP4UNPSEX)

IF 3 PARTNERS HAVE BEEN DISCUSSED AND PARTICIPANT HAD MORE THAN 4 PARTNERS CONTINUE WITH (SP5UNPSEX)

ADDITIONAL SEXUAL RISK QUESTIONS IF (SPHMANY) = 4 ONLY

SHOW IF (SPHMANY) = 4

VN: **(SP4UNPSEX)**

139. You said that you had had sex with one other person since your last visit. Did you ever have unprotected anal or vaginal sex with this person in this period?

1☐ Yes
2☐ No, never
3☐ Can't remember
99☐ No answer

SHOW IF (SPHMANY) = 4

VN: **(SP4YSTUDY)**

140. Have you ever talked with this person about your participation in this research study?

1☐ Yes
2☐ No
3☐ Don't know
99☐ No answer

ADDITIONAL SEXUAL RISK QUESTIONS IF (SPHMANY) > 4

SHOW IF (SPHMANY) > 4

VN: **(SP5UNPSEX)**

141. You said that you had had sex with several other persons since your last visit. Did you ever have unprotected anal or vaginal sex with any of these persons in this period?

1☐ Yes
2☐ No, never
3☐ Can't remember
99☐ No answer

SHOW IF (SPHMANY) > 4

VN: **(SP5YSTUDY)**

142. Have you ever talked with these persons about your participation in this research study?

1☐ Yes
2☐ No
3☐ Don't know
99☐ No answer

SHOW IF (SP#GENDER) = 2 (FEMALE) (IF PERSON HAD SEX WITH A WOMAN)

VN: **(SPFEMIW WITH MINOR WORDING CHANGE)**

143. You mentioned that you had sex with one or more women since your last visit. Do you think that if we want to interview her/them, this woman/these women would be willing to come to our study offices for an interview?

1☐ Definitively
2☐ Probably
3☐ Probably not
4☐ Definitively not
5☐ Don't know
99☐ No answer

HIV Risk Perception

	Week 13/ 3.0	Week 26/ 4.0	Week 39/ 5.0	Week 52/ 6.0
Relationship Status and Demographic Background	X	X	X	X
Sexual and Gender Identity, and Social Support		X		X
Resilience and Coping Self-Efficacy		X		
HIV Prevention	X	X	X	X
Sexual Behavior Assessment	X	X	X	X
**HIV Risk Perception**	**X**		**X**	
HIV/STI-Related Factors		X		X
HIV-Related Care and Treatment and HIV Stigma	X	X	X	X
Interest in HIV Prevention Strategies		X		
Alcohol and Drugs		X		X
Mental Health	X	X	X	X
Experience of Study Participation	X	X	X	X

SHOW HIVRCHANCE – HIVRNORISK ONLY IF THE PARTICIPANT IS HIV-NEGATIVE AND ONLY IF VISIT = 3.0 (WEEK 13) OR 5.O (WEEK 39)

VN: **HIVRCHANCE**

144. If you look at the sexual contacts that you have had since your last visit, is there a chance that you got infected with HIV?
   - 1 ☐ No chance
   - 2 ☐ Some chance
   - 99 ☐ No answer

To what extent do you agree or disagree with the following statements?	Disagree strongly = 1	Disagree = 2	Agree = 3	Agree strongly = 4	No answer = 99
145. [VN: **HIVRISK**]I am at risk for HIV	☐	☐	☐	☐	☐
146. [VN: **HIVRPOSSIBLE**]There is a possibility that I have HIV/AIDS	☐	☐	☐	☐	☐
147. [VN: **HIVRMIGHT**]I may have had sex with someone who was at risk for HIV	☐	☐	☐	☐	☐
148. [VN: **HIVRNORISK**]My sexual experiences do not put me at risk for HIV	☐	☐	☐	☐	☐

HIV/STI-Related Factors

	Week 13/ 3.0	Week 26/ 4.0	Week 39/ 5.0	Week 52/ 6.0
Relationship Status and Demographic Background	X	X	X	X
Sexual and Gender Identity, and Social Support		X		X
Resilience and Coping Self-Efficacy		X		
HIV Prevention	X	X	X	X
Sexual Behavior Assessment	X	X	X	X
HIV Risk Perception	X		X	
**HIV/STI-Related Factors**		**X**		**X**
HIV-Related Care and Treatment and HIV Stigma	X	X	X	X
Interest in HIV Prevention Strategies		X		
Alcohol and Drugs		X		X
Mental Health	X	X	X	X
Experience of Study Participation	X	X	X	X

SHOW (HIVAIDS) – (HIVNOTRI) IF VISIT = 4.0 (WEEK 26) OR 6.0 (WEEK 52)

The next questions are about HIV and sexually transmitted infections. Most people prefer to answer them privately, by reading and recording their responses on their own. Some people prefer that I continue to read the questions out loud and record their responses for them. Which would you prefer?

INTERVIEWER RADIO BUTTON

- **SELF-ADMINISTERED**
- **INTERVIEWER ADMINISTERED**

[SHOW IF SELF-ADMINISTERED]: *(Interviewer to read out loud FIRST):* As you read and respond to the questions in this section, I will stay in the room to help or to answer any questions you might have. You can skip any question you choose but please remember your responses are very important and will remain confidential.

[SHOW IF INTERVIEWER ADMINISTERED]: Ok, let's continue. Like I said, the following questions are about HIV, AIDS, other sexually transmitted infections and how they can be prevented.

[SHOW IF SELF-ADMINISTERED]: Please indicate whether each of the following statements are true or false, or that you do not know the answer by selecting the option next to the statement.

[SHOW IF INTERVIEWER ADMINISTERED]: Please indicate whether you think each of the following statements are true or false. You can also say that you don't know the answer.

	True = 1	False = 2	Do not know = 3	No answer = 99
149. [VN: **(HIVAIDS)**]HIV is a virus that weakens the immune system, leading to other infections, cancers, and AIDS	☐	☐	☐	☐
150. [VN: **(HIVCREDU)**]Using condoms when you have sex can reduce the chance of getting HIV	☐	☐	☐	☐

151. [VN: **(HIVCMORE)**]It is safe to use the same condom more than once	☐	☐	☐	☐
152. [VN: **(HIVVACCI)**]There is a vaccine that can stop people from getting HIV	☐	☐	☐	☐
153. [VN: **(HIVORALS)**]Oral sex is just as risky as anal intercourse for transmitting HIV	☐	☐	☐	☐
154. [VN: **(HIVSSCON)**]It is safe to have sex without a condom if it is with your regular partner	☐	☐	☐	☐
155. [VN: **(HIVOILUB)**]When you use condoms it is okay to also use oil-based lubricants	☐	☐	☐	☐
156. [VN: **(HIVPOUT)**] "Pulling out'' before the male ejaculates prevents transmission of HIV	☐	☐	☐	☐
157. [VN: **(HIVWASH)**]As long as both partners wash themselves after sex, it is not necessary to use condoms	☐	☐	☐	☐
158. [VN: **(HIVCURE)**]There is a cure for AIDS	☐	☐	☐	☐
159. [VN: **(HIVSEEIT)**]If someone has HIV you can see that straightaway	☐	☐	☐	☐
160. [VN: **HIVTANAL)**]HIV can be transmitted through anal sex	☐	☐	☐	☐

VN: **(HIVANALR)**

161. If two men have anal sex and do not use a condom, who is more at risk for HIV infection: the top, the bottom, or is the risk the same for both?
  1☐ Top more at risk
  2☐ Bottom more at risk
  3☐ Risk same for both
  4☐ Don't know
  99☐ No answer

[SHOW IF SELF-ADMINISTERED]: Choose the response that best represents to what extent you agree or disagree with the following statements.

[SHOW IF INTERVIEWER-ADMINISTERED]: To what extent you agree or disagree with the following statements? Do you disagree strongly, disagree, agree or agree strongly…

	Disagree strongly = 1	Disagree = 2	Agree = 3	Agree strongly = 4	No answer = 99
162. [VN: **(HIVSTIM)**]The use of condoms can make sex more stimulating	☐	☐	☐	☐	☐
163. [VN: **(HIVPLEASU)**]Condoms can be pleasurable	☐	☐	☐	☐	☐
164. [VN: **(HIVVALUE)**]Condoms go against my values or religious beliefs	☐	☐	☐	☐	☐

SHOW (HIVRISK) – (HIVNOTRI) ONLY IF PARTICIPANT IS HIV-NEGATIVE. IF HIV-POSITIVE, CONTINUE WITH THANK YOU STATEMENT BELOW (HIVNOTRI).

To what extent do you agree or disagree with the following statements?	Disagree strongly = 1	Disagree = 2	Agree = 3	Agree strongly = 4	No answer = 99
165. [VN: **(HIVRISK)**]I am at risk for HIV	☐	☐	☐	☐	☐
166. [VN: **(HIVPOSS)**]There is a possibility that I have HIV/AIDS	☐	☐	☐	☐	☐
167. [VN: **(HIVSEX)**]I may have had sex with someone who was at risk for HIV	☐	☐	☐	☐	☐
168. [VN: **(HIVNOTRI)**]My sexual experiences do not put me at risk for HIV	☐	☐	☐	☐	☐

[SHOW IF SELF-ADMINISTERED]: Thank you for completing that section on your own. Please tell the Study Staff that you have finished your section and are ready to continue the interview.

[SHOW IF INTERVIEWER ADMINISTERED]: Thank you for completing that section.

INTERVIEWER RADIO BUTTON

- **INTERVIEWER ADMINISTERED**

HIV-Related Care and Treatment and HIV Stigma

	Week 13/ 3.0	Week 26/ 4.0	Week 39/ 5.0	Week 52/ 6.0
Relationship Status	X		X	
Demographic Background		X		X
Sexual and Gender Identity, and Social Support		X		X
Resilience and Coping Self-Efficacy		X		
HIV Prevention	X	X	X	X
Sexual Behavior Assessment	X	X	X	X
HIV Risk Perception	X		X	
HIV/STI-Related Factors		X		X
HIV-Related Care and Treatment	X		X	
**HIV-Related Care and Treatment and HIV Stigma**	**X**	**X**	**X**	**X**
Interest in HIV Prevention Strategies		X		
Alcohol and Drugs		X		X
Mental Health	X	X	X	X
Experience of Study Participation	X	X	X	X

SHOW (CTSDOCTR) – (CTSWJUDG) ONLY IF PARTICIPANT IS HIV-POSITIVE AND IF VISIT = 3.0 (WEEK 13), 4.0 (WEEK 26), 5.0 (WEEK 39) OR 6.0 (WEEK 52). TO BE ASKED AFTER REFERRAL TO CARE HAS BEEN MADE.

VN: **(CTSDOCTR)**

169. Since your last visit, have you seen a doctor for the treatment of your HIV infection?

1☐ Yes → **GO TO (CTSDRRES)**
2☐ No
99 ☐ No Answer

SHOW IF (CTSDOCTR) = 2
VN: **(CTSDOCNO_OPEN)**

170. Can you explain why you haven't seen a doctor for the treatment of your HIV infection? **[OPEN TEXT]**

_________________ → **GO TO SELF-ADMINISTERED SECTION INTRO ABOVE (CTSDRUNC)**
2 ☐ No
99☐ No answer

SHOW (CTSDRRES) – (CTSMEDMIS) IF (CTSDOCTR) = 1
VN: **(CTSDRRES)**

171. When you went for treatment, did you feel respected by the doctor and nurses?

1☐ Yes
2☐ No
99 ☐ No Answer

VN: **(CTSDRUND)**

172. Do you feel that the doctor and nurses understood you?

1☐ Yes
2☐ No
99 ☐ No Answer

VN: **(CTSDRACC)**

173. Do you think the doctor and the nurses accept or reject sex between men?

1☐ Reject
2☐ Accept
3☐ Neutral
4☐ I don't know
99 ☐ No Answer

VN: **(CTSDRMED)**

174. Did the doctor prescribe you any medication for the treatment of your HIV infection?

1 ☐ Yes
2 ☐ No → **GO TO SELF-ADMINISTERED SECTION INTRO ABOVE (CTSDRUNC)**
99 ☐ No Answer → **GO TO SELF-ADMINISTERED SECTION INTRO ABOVE (CTSDRUNC)**

SHOW (CTSMEDPRE) – (CTSMEDMIS) IF (CTSDRMED) = 1

VN: **(CTSMEDPRE)**

175. Do you know what medication the doctor prescribed (for the treatment of HIV)?

Note: Make one comprehensive list of response options but only display certain options based on Site. Allow participant to select all drugs that apply.

If Site = Malawi:
1☐AZT
2☐3TC
3☐NVP
4☐EFV
5☐ATV/r
6☐LPV,r
7☐TDF
8☐Combivir (AZT, 3TC)
9☐Truvada (TDF, FTC)
10☐Single tablet TDF
11☐ATV/r
12☐D4T
13☐Triomune (D4T, 3TC, NVP) in single tablet
15☐Raltegrevir
15☐Cotrimoxazole

If Site = Soweto or Cape Town:
16☐Efavirenz
17☐Nevirapine
18☐Combivir (AZT and 3TC)
19☐Lamivudine
20☐FDC (fixed dose combination – Tenofovir, EFV, 3TC),
21☐Tenofovir
22☐Ritonavir
23☐Zidovudine
24☐Alluvia (RTV/LPV)
25☐Kaletra
26☐Abacavir

If Site = Kenya:
27☐Fixed dose regimen - AZT / 3TC
28☐AZT /3TC/ +NVP
29☐D4T / 3TC
30☐D4T / 3TC/ NVP
31☐TDF /3TC

32☐TDF/ 3TC/ EFV
33☐Didanosine
34☐Efavirenz
35☐Nevirapine
36☐Ritonavir
37☐Lopinavir
38☐Atazanavir
39☐Darunavir
40☐Raltegravir
41☐Elvitegravir
42☐Emtricitabine
43☐Lamivudine
44☐Stavudine
45☐Zidovudine
46☐CTX (Septrin/Cotrimoxazole)
47☐IPT (Isoniazid)
98☐ Don't Know **(SHOW ALL SITES THIS OPTION)**
99☐ No Answer **(SHOW ALL SITES THIS OPTION)**

VN: **(CTSMEDOFT)**

176. How often do you have to take the medication? **[OPEN TEXT]**

____
99☐ No answer

VN: **(CTSMEDPIL)**

177. Many people find it difficult to take their pills every day. In the last 30 days, how good a job did you do at taking your HIV medicines in the way you were supposed to? Would you say…

1☐ Very poor
2☐ Poor
3☐ Fair
4☐ Good
5☐ Very good
6☐ Excellent
99 ☐ No Answer

VN: **(CTSMEDSCA)**

178. Please indicate on a scale from 0 to 100 your best guess about how much of your HIV medicines you took as recommended over the last 30 days. 0% means that you have taken no pills, 50% means you have taken half of your pills, and 100% means you have taken every single pill.

1 ☐ 0
2 ☐ 10
3 ☐ 20
4 ☐ 30
5 ☐ 40
6 ☐ 50
7 ☐ 60
8 ☐ 70
9 ☐ 80
10 ☐ 90
11 ☐ 100
99 ☐ No Answer

VN: **(CTSMEDMIS)**

179. In the last 30 days, on how many days did you miss at least one dose of your HIV medicines?

_________________ days (range 0 - 30)

99☐ No answer

At this point, you may read and record your responses on your own. However, some people prefer that I read the questions out loud and record their responses for them. Which would you prefer?

INTERVIEWER RADIO BUTTON

- **SELF-ADMINISTERED**
- **INTERVIEWER ADMINISTERED**

PAGE BREAK

You will now be asked to respond to some questions about your experiences living with HIV. Your honest responses are very important to this study. [**IF SELF-AMINISTERED:** If you have any questions, please ask the Interviewer.]

Since you found out that you have HIV, has any healthcare provider . . .	Yes = 1	No = 2	No Answer = 99
180. [VN: **(CTSDRUNC)**]. . . Been uncomfortable with you?	☐	☐	☐
181. [VN: **(CTSDRINF)**].. . . Treated you as inferior?	☐	☐	☐
182. [VN: **(CTSDRAVO)**].. . . Preferred to avoid you?	☐	☐	☐
183. [VN: **(CTSDRREF)**].. . . Refused to see you for medical care?	☐	☐	☐

END OF SECTION IF VISIT = 3.0 OR 5.0. CONTINUE WITH NEXT SECTION (MENTAL HEALTH).

SHOW (CTSTELLP) - (CTSWJUDG) IF VISIT = 4.0 (WEEK 26) OR 6.0 (WEEK 52).

To what extent do you agree or disagree with the following statements?	Disagree strongly =1	Disagree =2	Agree =3	Agree strongly =4	No answer = 99
184. [VN: **(CTSTELLP)**] It is difficult to tell people about my HIV infection	☐	☐	☐	☐	☐
185. [VN: **(CTSDIRTY)**] Being HIV positive makes me feel dirty and unclean	☐	☐	☐	☐	☐
186. [VN: **(CTSGUILT)**] I feel guilty that I am HIV positive	☐	☐	☐	☐	☐
187. [VN: **(CTSASHAM)**] I am ashamed that I am HIV positive	☐	☐	☐	☐	☐
188. [VN: **(CTSWORTH)**] I sometimes feel worthless because I am HIV positive	☐	☐	☐	☐	☐
189. [VN: **(CTSIHIDE)**] I hide my HIV status from others	☐	☐	☐	☐	☐

To what extent do you agree or disagree with the following statements?	Disagree strongly = 1	Disagree = 2	Agree = 3	Agree strongly =4	No answer = 99
190. [VN: **(CTSREJECT)**] Some people close to me are afraid others will reject them if it becomes known that I have HIV	☐	☐	☐	☐	☐
191. [VN: **(CTSBACKA)**] People have physically backed away from me when they learn I have HIV	☐	☐	☐	☐	☐
192. [VN: **(CTSIGOODP)**] People who know I have HIV tend to ignore my good points	☐	☐	☐	☐	☐
193. [VN: **(CTSTOUCH)**] Some people avoid touching me once they know I have HIV	☐	☐	☐	☐	☐
194. [VN: **(CTSOCIAL)**] I have stopped socializing with some people because of their reactions to my having HIV	☐	☐	☐	☐	☐
195. [VN: **(CTSSTOP)**] People I care about stopped calling me after learning I have HIV	☐	☐	☐	☐	☐
196. [VN: **(CTSAFRAI)**] People seem afraid of me once they learn I have HIV	☐	☐	☐	☐	☐
197. [VN: **(CTSHURT)**] I have been hurt by how people reacted to learning I have HIV	☐	☐	☐	☐	☐
198. [VN: **(CTSCHILD)**] People don't want me around their children once they know I have HIV	☐	☐	☐	☐	☐
199. [VN: **(CTSFRIEN)**] I have lost friends by telling them I have HIV	☐	☐	☐	☐	☐
200. [VN: **(CTSDISTA)**] Some people who know I have HIV have grown more distant	☐	☐	☐	☐	☐
201. [VN: **(CTSUNCLE)**] Having HIV makes me feel unclean	☐	☐	☐	☐	☐
202. [VN: **(CTSIMBAD)**] Having HIV makes me feel that I'm a bad person	☐	☐	☐	☐	☐
203. [VN: **(CTSWORSE)**] People's attitudes about HIV make me feel worse about myself	☐	☐	☐	☐	☐
204. [VN: **(CTSNAGAP)**] I feel I am not as good a person as others because I have HIV	☐	☐	☐	☐	☐
205. [VN: **(CTSFAULT)**] Some people act as though it's my fault that I have HIV	☐	☐	☐	☐	☐
206. [VN: **(CTSISOLA)**] Since learning I have HIV, I feel set apart and isolated from the rest of the world	☐	☐	☐	☐	☐
207. [VN: **(CTSNHIDE)**] I never feel the need to hide the fact that I have HIV	☐	☐	☐	☐	☐
208. [VN: **(CTSWTELL)**] I worry that people who know I have HIV will tell others	☐	☐	☐	☐	☐
209. [VN: **(CTSRTOLD)**] I regret having told some people that I have HIV	☐	☐	☐	☐	☐
210. [VN: **(CTSWJUDG)**] I worry that people may judge me when they learn I have HIV	☐	☐	☐	☐	☐

IF VISIT = 6.0 (WEEK 52), CONTINUE TO ALCOHOL AND DRUG SECTION.

IF VISIT = 4.0 (WEEK 26) SHOW THE STATEMENTS BELOW.

[SHOW IF SELF-ADMINISTERED]: Thank you for completing that section on your own. Please tell the Study Staff that you have finished your section and are ready to continue the interview.

[SHOW IF INTERVIEWER ADMINISTERED]: Thank you for completing that section.

INTERVIEWER RADIO BUTTON

- **INTERVIEWER ADMINISTERED**

Interest in HIV Prevention Strategies

	Week 13/ 3.0	Week 26/ 4.0	Week 39/ 5.0	Week 52/ 6.0
Relationship Status and Demographic Background	X	X	X	X
Sexual and Gender Identity, and Social Support		X		X
Resilience and Coping Self-Efficacy		X		
HIV Prevention	X	X	X	X
Sexual Behavior Assessment	X	X	X	X
HIV Risk Perception	X		X	
HIV/STI-Related Factors		X		X
HIV-Related Care and Treatment and HIV Stigma	X	X	X	X
**Interest in HIV Prevention Strategies**		**X**		
Alcohol and Drugs		X		X
Mental Health	X	X	X	X
Experience of Study Participation	X	X	X	X

THIS SECTION MUST BE INTERVIEWER ADMINISTERED. SHOW IF VISIT = 4.0.

VN: **INTLEARN**

211. Do you feel that you know enough about HIV and AIDS or would you like to learn more?
1 ☐ Know enough → **GO TO INTWAYS**
2 ☐ Would like to know more
99 ☐ No answer → **GO TO INTWAYS**

SHOW IF INTLEARN = 2 (WOULD LIKE TO KNOW MORE)
VN: **INTTOPIC**
212. Are there specific topics that you would like to learn more about? **[OPEN TEXT]**

____
2 ☐ No
99 ☐ No answer

VN: **INTWAYS**

213. We would like to talk with you now about ways in which people prevent transmission of HIV. What ways do you know that can be used to prevent HIV transmission? *(Check all that apply).*
INTWAYS_1 ☐ Use of condoms for anal sex
INTWAYS_2 ☐ Use of condoms and water-based lubrication for anal sex
INTWAYS_3 ☐ Use of condoms for vaginal intercourse
INTWAYS_4 ☐ Abstinence (no anal sex or vaginal intercourse)
INTWAYS_5 ☐ Pre-exposure prophylaxis (PrEP)
INTWAYS_6 ☐ Post-exposure prophylaxis (PEP)
INTWAYS_7 ☐ Reduce number of sexual partners
INTWAYS_8 ☐ Being faithful to one partner
INTWAYS__9 ☐ Other, specify: ___________________
INTWAYS_99 ☐ No answer

INTPEP – INTCHOICE ARE ONLY FOR MEN WHO ARE HIV-NEGATIVE. IF THE PARTICIPANT IS HIV-POSITIVE, CONTINUE WITH NEXT SECTION (ALCOHOL AND DRUGS).

VN: **INTPEP**

214. Have you ever heard of PEP, post-exposure prophylaxis?
   1 ☐ Yes
   2 ☐ No
   99 ☐ No answer

{IF INTPEP = 1: Just to make sure it's clear what we are talking about,**}** PEP is the use of HIV medications as soon as possible after exposure to HIV to prevent your body from becoming infected with HIV. Emergency PEP contains the same medicines that people with HIV take to stay healthy. These medicines stop the virus from multiplying in your body. PEP can reduce the chance of infection if you are exposed to HIV. PEP can for instance be taken after a condom broke or because you didn't use a condom but had sex with someone who was HIV positive.

VN: **INTUSEPEP**

215. If you were to have an exposure to HIV in the next 12 months, how likely would you be interested in using PEP to prevent HIV if it would be available to you?
   5 ☐ Very likely
   4 ☐ Somewhat likely
   3 ☐ Neither likely or unlikely
   2 ☐ Somewhat unlikely
   1 ☐ Very unlikely
   99 ☐ No answer

VN: **INTSTILLPEP**

216. With PEP, you take HIV medications for 28 days, usually once or twice a day. How hard or how easy would it be for you to take HIV medications for 28 days?
   1 ☐ Very hard
   2 ☐ Hard
   3 ☐ Neutral
   4 ☐ Easy
   5 ☐ Very easy
   99 ☐ No answer

VN: **INTPREP**

217. I would now like to talk with you about PrEP. PrEP stands for Pre-exposure prophylaxis. Have you ever heard of PrEP?
   1 ☐ Yes
   2 ☐ No
   99 ☐ No answer

{IF INTPREP = 1: Just to make sure it's clear what we are talking about,**}** With PrEP you take HIV medication to reduce the chances of getting HIV infected. You would need to take this medication every day whether you planned to have sex that day or not. If you don't take it every day PrEP is not as effective.

VN: **INTUSEPREP**

218. If it would be possible for you to get PrEP, how interested would you be in using it?
   1 ☐ Very interested
   2 ☐ Somewhat interested
   3 ☐ Not interested → **GO TO VARIABLE INTINJECT**
   99 ☐ No answer → **GO TO VARIABLE INTINJECT**

SHOW IF INTUSEPREP < 3
VN: **INTSTILLPREP**
219. The medication would have to be prescribed by a doctor. That doctor would need to see you at least every 3 months to test you for HIV infection and other medical checkups. Would you still be interested in PrEP?
1 ☐ Very interested
2 ☐ Somewhat interested
3 ☐ Not interested → **GO TO VARIABLE INTINJECT**
99 ☐ No answer → **GO TO VARIABLE INTINJECT**

SHOW IF INTSTILLPREP < 3
VN: **INTSIDEEFFECT**
220. There are some side effects reported by people who start taking HIV medication, mostly nausea and weight loss that goes away after the first month or so. In rare cases, HIV medication taken for long periods can damage the kidneys. Given these side effects, would you still be interested in PrEP?
1 ☐ Very interested
2 ☐ Somewhat interested
3 ☐ Not interested → **GO TO VARIABLE INTINJECT**
99 ☐ No answer → **GO TO VARIABLE INTINJECT**

SHOW IF INTSIDEEFFECT < 3
VN: **INT3MON**
221. How hard or how easy would it be for you to take PrEP every day and to see a doctor every three months for checkups?
1 ☐ Very hard
2 ☐ Hard
3 ☐ Neutral
4 ☐ Easy
5 ☐ Very easy
99 ☐ No answer

VN: **INTINJECT**
222. Imagine that you could get PrEP via an injection. You would have to see a doctor once every three months. The injection that the doctor gives you would reduce your chances of getting infected with HIV for three months. And then you would have to see the doctor again. This is called injectible PrEP. How interested would you be in having injectible PrEP? Would you say…
1 ☐ Very interested
2 ☐ Somewhat interested
3 ☐ Not interested
99 ☐ No answer

SHOW ALL
VN: **INTMICROB**
223. I would now like to talk with you about Microbicides. Have you ever heard of Microbicides?
1 ☐ Yes
2 ☐ No
99 ☐ No answer

{IF INTMICROB = 1: Just to make sure it's clear what we are talking about,**}** Microbicides are gels or creams that people can put in their rectum or vagina to prevent HIV infection. At this moment there are no microbicides that have proven to be effective. Imagine that an effective microbicide would become available.

VN: **INTUSEMICROB**

224. If you were having anal sex with a partner in the next 12 months, how likely would you be to use rectal microbicides if you were the bottom partner?

1 ☐ Very likely
2 ☐ Somewhat likely
3 ☐ Neither likely or unlikely
4 ☐ Somewhat unlikely
5 ☐ Very unlikely
6 ☐ I don't have receptive anal intercourse
99 ☐ No answer

VN: **INTPRESUA**

225. How likely would you be to persuade your partner to use rectal microbicides if you were the top partner?

1 ☐ Very likely
2 ☐ Somewhat likely
3 ☐ Neither likely or unlikely
4 ☐ Somewhat unlikely
5 ☐ Very unlikely
6 ☐ I don't have insertive anal intercourse
99 ☐ No answer

PAGE BREAK

Several companies are developing kits that could be purchased and would allow for testing by you in your home, and you would have the result in less than one hour. The whole test and the results would be done at home.

VN: **INTHIVTEST**

226. If such home test kits were available, would you be interested in buying and using it, or would you rather go to a clinic or doctor to find out about your HIV status?

1 ☐ Would be interested in buying and using it
2 ☐ Would rather go to a clinic or doctor
3 ☐ Do not know
99 ☐ No answer

VN: **INTTESTPART**

227. If such home test kits were available, how likely is it that you would ask a sex partner to use it and test himself before having sex?

5 ☐ Very likely
4 ☐ Likely
3 ☐ Neutral
2 ☐ Unlikely
1 ☐ Very unlikely
99 ☐ No answer

PAGE BREAK

Some types of HIV prevention strategies require frequent HIV testing. If you were participating in an HIV prevention program that required frequent HIV testing, would you consider performing testing at home if it required that…:

	Definitely would = 4	Possibly would = 3	Probably would not = 2	Definitely would not = 1	No answer = 99
228. [VN: **INTPRICK**]…you prick your finger to get a drop of blood	☐	☐	☐	☐	☐
229. [VN: **INTSWAB**]…you swab your mouth with a Q-tip	☐	☐	☐	☐	☐
230. [VN: **INTREADSELF**]…you read the test results yourself	☐	☐	☐	☐	☐
231. [VN: **INTCALLRESULT**]…you mail in the test and call in for results	☐	☐	☐	☐	☐

There are different ways in which people can learn more about HIV and AIDS. I will mention several ways this can be done and would like to hear from you whether how much you would like or dislike learning more about HIV in this way.

	Like very much = 5	Like = 4	No preference = 3	Dislike = 2	Dislike very much = 1	No answer = 99
232. [VN: **INTBROCHURES**] Via brochures and pamphlets	☐	☐	☐	☐	☐	☐
233. [VN: **INTINTERNET**] Via the internet	☐	☐	☐	☐	☐	☐
234. [VN: **INTINDIVIDUAL**] In individual meetings with a prevention worker	☐	☐	☐	☐	☐	☐
235. [VN: **INTMENSEX**] In groups with other men who have sex with men	☐	☐	☐	☐	☐	☐
236. [VN: **INTDOCTOR**] In person from a medical doctor or a nurse	☐	☐	☐	☐	☐	☐
237. [VN: **INTCHURCH**] In a mosque, church, or religious group	☐	☐	☐	☐	☐	☐
238. [VN: **INTFRIENDS**] From friends	☐	☐	☐	☐	☐	☐
239. [VN: **INTFAMILY**] From family	☐	☐	☐	☐	☐	☐

VN: **INTCHOICE**

240. Which of the various ways that we discussed would you prefer most?

1 ☐ Brochures and pamphlets
2 ☐ Internet
3 ☐ Individual meetings with a prevention worker
4 ☐ Groups with other men who have sex with men
5 ☐ Medical doctor or nurse
6 ☐ Mosque, church, or religious group
7 ☐ Friends
8 ☐ Family
99 ☐ No answer

Alcohol and Drugs

	Week 13/ 3.0	Week 26/ 4.0	Week 39/ 5.0	Week 52/ 6.0
Relationship Status and Demographic Background	X	X	X	X
Sexual and Gender Identity, and Social Support		X		X
Resilience and Coping Self-Efficacy		X		
HIV Prevention	X	X	X	X
Sexual Behavior Assessment	X	X	X	X
HIV Risk Perception	X		X	
HIV/STI-Related Factors		X		X
HIV-Related Care and Treatment and HIV Stigma	X	X	X	X
Interest in HIV Prevention Strategies		X		
**Alcohol and Drugs**		**X**		**X**
Mental Health	X	X	X	X
Experience of Study Participation	X	X	X	X

SHOW IF VISIT = 4.0 (WEEK 26) OR 6.0 (WEEK 52)

***IF VISIT = 4.0 (WEEK 26): SHOW THE STATEMENT BELOW AND OFFER “SELF-ADMINISTERED” OR “INTERVIEWER ADMINISTERED”.**

***IF VISIT = 6.0 (WEEK 52): IF SELF-ADMINISTERED WAS SELECTED DURING “HIV-RELATED CARE AND TREATMENT AND HIV STIGMA” SECTION AT VISIT 6.0 (WEEK 52), MAINTAIN SELF-ADMINISTERED AND SKIP DIRECTLY TO AODOFTEN. IF INTERVIEW-ADMINISTERED WAS SELECTED DURING “HIV-RELATED CARE AND TREATMENT AND HIV STIGMA” SECTION, SHOW THE STATEMENT BELOW AND OFFER “SELF-ADMINISTERED” OR “INTERVIEWER ADMINISTERED” AGAIN.**

You will now be asked to respond to some questions about your experiences with alcohol and drugs. At this point, you may read and record your responses on your own. However, some people prefer that I read the questions out loud and record their responses for them. Which would you prefer?

INTERVIEWER RADIO BUTTON

- **SELF-ADMINISTERED**
- **INTERVIEWER ADMINISTERED**

[SHOW IF SELF-ADMINISTERED]: Your honest responses are very important to this study. If you have any questions, please ask the Interviewer.

VN: **(AODOFTEN)**

241. How often do you have a drink containing alcohol?

1☐ Never → **GO TO (AODPXDG)**
2☐ Monthly or less
3☐ Two to four times a month
4☐ Two to three times a week
5☐ Four or more times a week
99☐ No answer

SHOW IF (AODOFTEN) IS >1 (IF PARTICIPANT HAS HAD A DRINK)

VN: **(AODMANY)**

242. How many drinks containing alcohol do you have on a typical day when you are drinking?

1☐ 1 or 2
2☐ 3 or 4
3☐ 5 or 6
4☐ 7 to 9
5☐ 10 or more
99☐ No answer

SHOW IF (AODOFTEN) IS >1 (IF PARTICIPANT HAS HAD A DRINK)

VN: **(AODBINGE)**

243. How often do you have six or more drinks on one occasion?

1 ☐Never
2 ☐Less than monthly
3 ☐Monthly
4 ☐Weekly
5 ☐Daily or almost daily
99☐ No answer

The next questions are about other substances you could have used, including substances prescribed by a doctor (like pain medications) that you might have taken for reasons or in doses other than prescribed, and recreational or illegal drugs.

When referring to these substances, please include any of the following:

- Cannabis (marijuana, dagga, bhang, ganja, puga, pot, grass, hash, etc.)
- Nyaope (whoonga or wunga; ARV mixed with other drugs)
- Inhalants (turpentine, nitrous oxide, shoe glue, glue, petrol, gas, paint thinner, etc.)
- Methamphetamine (tik, speed, crystal meth, ice, etc.)
- Sedatives or sleeping pills (mandrax, Valium, Serepax, Ativan, Xanax, Librium, Rohypnol, GHB, etc.)
- Cocaine (coke, crack, etc.)
- Street opioids (heroin, opium, etc.)
- Prescription opioids (fentanyl, oxycodone [OxyContin, Percocet], hydrocodone [Vicodin], methadone, buprenorphine, etc.).
- Prescription stimulants (Ritalin)

	Never = 1	Once or twice = 2	Monthly = 3	Weekly = 4	Daily or almost daily = 5	No answer = 99
244. [VN: **(AODPXDG)**] In the past 6 months, how often have you used prescription drugs for non-medical reasons?	☐	☐	☐	☐	☐	☐
245. [VN: **(AODRECD)**] In the past 6 months, how often have you used recreational drugs?	☐	☐	☐	☐	☐	☐

SHOW IF (AODPXDG) >1 OR (AODRECD) > 1 (IF DRUGS HAVE EVER BEEN USED)

VN: **(AODHUSE)**

246. Some people smoke, swallow, or inject drugs. How do you use drugs? You can choose more than one response

(AODHUSE_1)☐ Smoke
(AODHUSE_2)☐ Swallow
(AODHUSE_3)☐ Inject
(AODHUSE_4)☐ Other
(AODHUSE_99)☐ No answer

SHOW IF (AODHUSE_3) IS CHECKED (IF PARTICIPANT INJECTED DRUGS)

VN: **(AODINJECT)**

247. Have you in the past 6 months ever shared needles with somebody else?

1☐ Yes
2☐ No
99☐ No answer

Mental Health

	Week 13/ 3.0	Week 26/ 4.0	Week 39/ 5.0	Week 52/ 6.0
Relationship Status and Demographic Background	X	X	X	X
Sexual and Gender Identity, and Social Support		X		X
Resilience and Coping Self-Efficacy		X		
HIV Prevention	X	X	X	X
Sexual Behavior Assessment	X	X	X	X
HIV Risk Perception	X		X	
HIV/STI-Related Factors		X		X
HIV-Related Care and Treatment and HIV Stigma	X	X	X	X
Interest in HIV Prevention Strategies		X		
Alcohol and Drugs		X		X
**Mental Health**	**X**	**X**	**X**	**X**
Experience of Study Participation	X	X	X	X

SHOW IF VISIT = 3.0 (WEEK 13), 4.0 (WEEK 26), 5.0 (WEEK 39) OR 6.0 (WEEK 52).

***MAINTAIN SELF-ADMINISTERED OR INTERVIEWER-ADMINISTERED SETTING. IF PARTICIPANT HASN'T YET BEEN GIVEN A CHANCE TO SELECT SELF-ADMINISTERED (E.G. IF VISIT 3.0 OR 5.0 AND PARTICIPANT IS HIV-NEGATIVE, PARTICIPANT WILL NOT SEE THIS OPTION UNTIL THIS SECTION), PROVIDE OPTION TO SELECT SELF-ADMINISTERED.**

The next questions are about how you feel about yourself.

{IF VISIT =3.0 OR 5.0 AND PARTICIPANT IS HIV-NEGATIVE} Some people prefer to answer them privately, by reading and recording their responses on their own. Some people prefer that I continue to read the questions out loud and record their responses for them. Which would you prefer?

INTERVIEWER RADIO BUTTON

- **SELF-ADMINISTERED**
- **INTERVIEWER ADMINISTERED**

SHOW ALL

VN: **(MHSELFEST)**

248. To what extent do you agree or disagree with the following statement: I have high self-esteem. With "self-esteem" we mean having a favorable opinion of yourself.
 1☐ Disagree strongly
 2☐ Disagree
 3☐ Agree
 4☐ Agree strongly
 99☐ No answer

VN: **(MHANXIET)**

249. In the last 4 weeks, since **{TODAY-4 weeks}**, have you had an anxiety attack — suddenly feeling fear or panic?
 1☐ Yes
 2☐ No → **GO TO (DEPNOINT)**
 99☐ No answer

SHOW IF (MHANXIET) = 1

VN: **(MHANXEVR)**

250. Has this ever happened before?

1☐ Yes
2☐ No
99☐ No answer

Over the last 2 weeks, how often have you been bothered by any of the following problems?

**[IF INTERVIEWER ADMIN]:** For each please respond with not at all, several days, more than half the days or nearly every day.	Not at all = 1	Several days = 2	More than half the days = 3	Nearly everyday = 4	No answer = 99
251. [VN: **(DEPNOINT)**] Little interest or pleasure in doing things	☐	☐	☐	☐	☐
252. [VN**: (DEPRESSD)**] Feeling down, depressed, or hopeless	☐	☐	☐	☐	☐
253. [VN: **(DEPSLEEP)**]Trouble falling or staying asleep, or sleeping too much	☐	☐	☐	☐	☐
254. [VN: **(DEPTIRED)**] Feeling tired or having little energy	☐	☐	☐	☐	☐
255. [VN: **(DEPOREAT)**] Poor appetite or overeating	☐	☐	☐	☐	☐
256. [VN: **(DEPDOWN)**] Feeling bad about yourself - or that you are a failure or have let yourself or your family down	☐	☐	☐	☐	☐
257. [VN: **(DEPTHINK)**] Trouble concentrating on things, such as reading the newspaper or watching television	☐	☐	☐	☐	☐
258. [VN: **(DEPMOVE)**] Moving or speaking so slowly that other people could have noticed	☐	☐	☐	☐	☐
259. [VN: **(DEPRESTL)**] Or the opposite - being so fidgety or restless that you have been moving around a lot more than usual	☐	☐	☐	☐	☐
260. [VN: **(DEPHURTS)**] Thoughts that you would be better off dead, or of hurting yourself in some way	☐	☐	☐	☐	☐

Experience of Study Participation

	Week 13/ 3.0	Week 26/ 4.0	Week 39/ 5.0	Week 52/ 6.0
Relationship Status and Demographic Background	X	X	X	X
Sexual and Gender Identity, and Social Support		X		X
Resilience and Coping Self-Efficacy		X		
HIV Prevention	X	X	X	X
Sexual Behavior Assessment	X	X	X	X
HIV Risk Perception	X		X	
HIV/STI-Related Factors		X		X
HIV-Related Care and Treatment and HIV Stigma	X	X	X	X
Interest in HIV Prevention Strategies		X		
Alcohol and Drugs		X		X
Mental Health	X	X	X	X
**Experience of Study Participation**	**X**	**X**	**X**	**X**

***MAINTAIN SELF-ADMINISTERED OR INTERVIEWER-ADMINISTERED SETTING**

The following questions are about your participation in the study. **[SHOW FOLLOWING ADDITIONAL SENTENCE IF VISIT =4.0 (WEEK 26) OR 6.0 (WEEK 52):** The first set of questions is about how you felt answering the questions in the interview.**]**

SHOW (PRTQSAD) – (PRTQBADT) IF VISIT = 4.0 (WEEK 26) OR 6.0 (WEEK 52)

How did you feel about answering the questions? To what extent do you agree or disagree with the following statements?	Disagree strongly = 1	Disagree = 2	Agree = 3	Agree strongly = 4	No answer = 99
261. [VN: **(PRTQSAD)**] Some questions made me sad	☐	☐	☐	☐	☐
262. [VN: **(PRTQLIKE)**] I liked that I was able to give my opinion	☐	☐	☐	☐	☐
263. [VN: **(PRTQEASY)**] All questions were easy to understand	☐	☐	☐	☐	☐
264. [VN: **(PRTQDIFF)**] Some questions were difficult to answer	☐	☐	☐	☐	☐
265. [VN: **(PRTQDOWN)**] Some questions made me feel down	☐	☐	☐	☐	☐
266. [VN: **(PRTQHELP)**] I felt a need for help due to the questions	☐	☐	☐	☐	☐
267. [VN: **(PRTQRELIE)**] I found it a relief to share my experiences	☐	☐	☐	☐	☐
268. [VN: **(PRTQBADT)**] The questions gave me bad thoughts about things that happened to me	☐	☐	☐	☐	☐

SHOW (PRTQSEXB) IF VISIT = 3.0 (WEEK 13), 4.0 (WEEK 26), 5.0 (WEEK 39) OR 6.0 (WEEK 52)

VN: **(PRTQSEXB)**

269. How comfortable or uncomfortable did you feel answering the questions about sexual behavior?

1☐ Very comfortable
2☐ Comfortable
3☐ Uncomfortable
4☐ Very uncomfortable
99☐ No answer

SHOW (PRTQAOD) IF VISIT = 4.0 (WEEK 26) OR 6.0 (WEEK 52)

VN: **(PRTQAOD)**

270. How comfortable or uncomfortable did you feel answering the questions about drug and alcohol use?

1☐ Very comfortable
2☐ Comfortable
3☐ Uncomfortable
4☐ Very uncomfortable
99☐ No answer

SHOW (PRTCLEAR) – (PRTMPROC) IF VISIT = 3.0 (WEEK 13), 4.0 (WEEK 26), 5.0 (WEEK 39) OR 6.0 (WEEK 52)

The next set of questions is about your participation in this study more generally.

Please indicate whether you agree or disagree with the following statements:	Disagree strongly = 1	Disagree = 2	Agree = 3	Agree strongly = 4	No answer = 99
271. [VN: **(PRTCLEAR)**] All study procedures are clearly explained	☐	☐	☐	☐	☐
272. [VN: **(PRTRESPT)**] I feel respected by the research staff	☐	☐	☐	☐	☐
273. [VN: **(PRTPRIVT)**] The research staff keeps my personal information completely private	☐	☐	☐	☐	☐

[IF SELF-ADMINISTERED]: Thank you. You have finished this self-administered section. Please tell the clinic staff person that you have finished your section and are ready to continue the interview.

[IF INTERVIEWER ADMINISTERED]: Thank you for completing that section.

INTERVIEWER RADIO BUTTON

- **INTERVIEWER ADMINISTERED**

I will now continue to read questions to you about your experiences with this study. Do you have any questions before we continue?

VN: **(PRTCOMFY)**

274. Do you feel comfortable with all the medical procedures requested for this study?

1☐ Yes → **GO TO PRTLAST IF VISIT = 3.0 (WEEK 13) OR 5.0 (WEEK 39); GO TO PRTRJOIN IF VISIT = 4.0 (WEEK 26); GO TO PRTSTOP IF VISIT = 6.0 (WEEK 52)**
2☐ No
99☐ No answer

SHOW IF (PRTCOMFY) = 2

VN: **(PRTMPROC)**

275. Can you tell which medical procedures you are not comfortable with and why that is so? **[OPEN TEXT]**

____

99☐ No answer

SHOW PRTSTOP – PRTREASON IF VISIT = 6.0 (WEEK 52)

VN: **PRTSTOP**

276. Have you ever considered stopping with your participation in this study?

1☐ Yes
2☐ No → **GO TO (PRTRJOIN)**
99☐ No answer → **GO TO (PRTRJOIN)**

SHOW IF PRTSTOP= 1 (YES)

VN: **PRTREASON**

277. What was the main reason why you considered stopping with your participation?

1☐ Difficulty arranging visits
2☐ No time/competing interests
3☐ Study burden too high
4☐ Study not relevant
5☐ Negative experiences in the study
6☐ Other, please specify: ______________________________
99☐ No answer

SHOW (PRTRJOIN) – (PRTRCONT) IF VISIT = 4.0 (WEEK 26) OR 6.0 (WEEK 52)

VN: **(PRTRJOIN)**

278. What was the single most important reason you decided to join this study? **[OPEN TEXT]**

____

99☐ No answer

VN: **(PRTRCONT)**

279. What is the single most important reason you continue to participate in this study? **[OPEN TEXT]**

____

99☐ No answer

SHOW (PRTCOMM) IF VISIT = 4.0 (WEEK 26)

VN: **(PRTCOMM)**

280. How committed do you feel to this study?

1☐ Very committed
2☐ Committed
3☐ Moderately committed
4☐ Not committed
99☐ No answer

SHOW PRTCONTHELP – PRTSIGBRD IF VISIT = 4.0 (WEEK 26) OR 6.0 (WEEK 52)

We would like to know how important each of the following reasons is for you in continuing to participate in the study. Please indicate if the reason is very important, moderately important, slightly important or not important at all.

	Very important = 4	Moderately important = 3	Slightly important = 2	Not important at all = 1	No answer = 99
281. [VN: **PRTCONTHELP**] Contributing to research that may help my community	☐	☐	☐	☐	☐
282. [VN: **PRTCOMMIT**] Keeping the commitment I made when I decided to enroll in this study	☐	☐	☐	☐	☐
283. [VN: **PRTLEARNHIV**] Being in a position to learn about HIV prevention	☐	☐	☐	☐	☐
284. [VN: **PRTHEALTHCARE**] Getting better health care	☐	☐	☐	☐	☐
285. [VN: **PRTFRIENDHIV**] Doing something for my friends who died of HIV	☐	☐	☐	☐	☐
286. [VN: **PRTMONEY**] Receiving money for my participation in the study	☐	☐	☐	☐	☐
287. [VN: **PRTCONTINUE**] Other people wanted me to continue with the study	☐	☐	☐	☐	☐
288. [VN: **PRTFREE**] Receiving free condoms and lubrication	☐	☐	☐	☐	☐

VN: **PRTOTHREASON**

289. Are there any other reasons not already mentioned that are important for you in continuing to participate in the study? Please specify:________________________**[OPEN TEXT]**
   - 2 ☐ No
   - 99☐ No answer

SHOW (PRTIMPORT) IF VISIT = 6.0 (WEEK 52)

VN: **(PRTIMPORT)**

290. How important or unimportant do you consider this study to be for your community?
   - 1☐ Very Important
   - 2☐ Important
   - 3☐ Moderately important
   - 4☐ Not important at all
   - 99☐ No answer

There are several things that might make it difficult for you to participate in this study. I will mention a few things and I would like to hear from you whether it is easy or difficult for you to do these.

	Very easy = 1	Easy = 2	Difficult = 3	Very difficult = 4	No answer = 99
291. [VN: **(PRTCLINC)**] Travelling to the study clinic	☐	☐	☐	☐	☐
292. [VN: **(PRTTIME)**] Making time to come for study visits	☐	☐	☐	☐	☐
293. [VN: **(PRTAPPTS)**] Setting up appointments for a study visit	☐	☐	☐	☐	☐

VN: **(PRTBURDN)**

294. Overall, considering the time you spent and any anxiety, or discomfort you may have experienced in this study, how difficult has it been for you to participate in this study?

4☐Very difficult
3☐Moderately difficult
2☐Slightly difficult
1☐Not difficult at all
99☐ No answer

SHOW IF (PRTBURDN)>1
VN: **(PRTSIGBRD)**

295. What are the most significant burdens thus far for you of participating in this study? **[OPEN TEXT]**

____
99☐ No answer

SHOW IF VISIT = 3.0 (WEEK 13), 4.0 (WEEK 26), 5.0 (WEEK 39) OR 6.0 (WEEK 52)
VN: **PRTLAST**

296. Did anything particular happen since your last visit that made you want to consider stopping study participation? **[OPEN TEXT]**

____
2 ☐ No
99☐ No answer

SHOW PRTTALK – PRTWHOMPRESS IF VISIT = 4.0 (WEEK 26) OR 6.0 (WEEK 52)
VN: **PRTTALK**

297. **IF VISIT = 4.0:** Since you started participating in this study, have you talked with someone about your participation?
IF VISIT = 6.0: In the past 6 months, have you talked with someone about your participation in this study?

1 ☐ Yes
2 ☐ No → **GO TO PRTOTHER**
99 ☐ No answer → **GO TO PRTOTHER**

SHOW IF PRTTALK = 1
VN: **PRTFAVOR**

298. Was their response to your participation in this study favorable or unfavorable?

4 ☐ Very favorable
3 ☐ Favorable
2 ☐ Unfavorable
1 ☐ Very unfavorable
5 ☐ Do not know/not applicable
99 ☐ No answer

VN: **(PRTOTHER)**

299. Do you know any men like you who also have sex with men who are not participating in this study?

1☐ Yes
2☐ No → **GO TO PRTOTHPRESS**
99☐ No answer → **GO TO PRTOTHPRESS**

SHOW IF (PRTOTHER) = 1
VN: **(PRTOTH_OPEN)**

300. Do you have any idea why these men are not participating in this study? **[OPEN TEXT]**

____
2 ☐ No
99☐ No answer

VN: **PRTOTHPRESS**

301. How much pressure, if any, do you feel from other people (including the research team) to continue in the study?

4 ☐ A great deal
3 ☐ Some
2 ☐ A little
1 ☐ None → **GO TO (PRTFAMLY)**
99☐ No answer → **GO TO (PRTFAMLY)**

SHOW IF PRTOTHPRESS > 1

VN: **PRTWHOMPRESS**

302. From whom do you feel pressure? *Mark all that apply.*

PRTWHOMPRESS_1 ☐ The research team
PRTWHOMPRESS_2 ☐ Other participants in the study
PRTWHOMPRESS_3 ☐ Close friend(s)
PRTWHOMPRESS_4 ☐ Family member(s)
PRTWHOMPRESS_5 ☐ Others, please specify: ______________________________
PRTWHOMPRESS_99☐ No answer

SHOW (PRTFAMLY) – (PRTEVENT_OPEN) IF VISIT = 3.0 (WEEK 13), 4.0 (WEEK 26), 5.0 (WEEK 39) OR 6.0 (WEEK 52)

Because of your participation in this study, have you, since your last visit…	Yes = 1	No = 2	No answer = 99
303. [VN: **(PRTFAMLY)**]. . . Had personal trouble with family?	☐	☐	☐
304. [VN: **PRTFRIEND**]. . . Had personal trouble with friends or acquaintances?	☐	☐	☐
305. [VN: **PRTHOUSE]**. . . Had trouble getting or keeping housing?	☐	☐	☐
306. [VN: **PRTECON]**. . . Had trouble getting or keeping a job or trouble with income or economic support?	☐	☐	☐
307. [VN: **PRTHEALTH]**. . . Had trouble with health insurance or getting health care?	☐	☐	☐
308. [VN: **PRTLEGAL]**. . . Been arrested or had trouble with the police or other legal problems?	☐	☐	☐
309. [VN: **PRTPROB]**. . . Had any other type of problem?	☐	☐	☐

SHOW IF ANY ONE OF (PRTFAMLY) TO (PRTPROB) = 1 (YES)

VN: **(PRTEVENT)**

310. How often have these events happened since your last visit? Would you say…

1☐ Only once
2☐ A few times
3☐ Regularly
4☐ Often
5☐ Very often
99☐ No answer

VN: **(PRTEVENT_OPEN)**

311. Because of your participation in this study, did anything negative or bad happen to you that you have not reported to us already? **[OPEN TEXT]**

____

2 ☐ No
99☐ No answer

END OF QUESTIONNAIRE IF VISIT = 3.0 (WEEK 13) OR 5.0 (WEEK 39). GO TO END SCREEN.

SHOW PRTMEDBENEFIT – PRTOTHBENEFIT IF VISIT = 4.0 (WEEK 26) OR 6.0 (WEEK 52)

VN: **PRTMEDBENEFIT**

312. Overall, how much medical benefit (including vaccination, improvement in your physical health and/or increased access to medical services) do you think you are getting from participating in this study?

4 ☐ A great deal of medical benefit
3 ☐ A moderate amount of medical benefit
2 ☐ A small amount of medical benefit
1 ☐ No medical benefit → **GO TO PRTNONMEDBENIFIT**
99☐ No answer → **GO TO PRTNONMEDBENIFIT**

SHOW IF PRTMEDBENEFIT > 1

VN: **PRTMEDDES**

313. In your view, what are the primary medical benefits of participating in this study? **[OPEN TEXT]**

____

99☐ No answer

VN: **PRTNONMEDBENEFIT**

314. To what extent do you get non-medical benefits from participating in this study, such as the opportunity to meet new friends, feelings of support, hope, or satisfaction?

4 ☐ A great deal of non-medical benefit
3 ☐ A moderate amount of non-medical benefit
2 ☐ A small amount of non-medical benefit
1 ☐ No non-medical benefit → **GO TO PRTIMPORTANT**
99☐ No answer → **GO TO PRTIMPORTANT**

SHOW IF PRTNONMEDBENEFIT > 1

VN: **PRTNONMEDDES**

315. Please describe what these non-medical benefits are. **[OPEN TEXT]**

____

99☐ No answer

In what ways do you think you might have benefited from participating in this study? Please indicate whether you agree or disagree with the following statements:	Disagree strongly = 1	Disagree = 2	Neutral = 3	Agree = 4	Agree strongly = 5	No answer = 99
316. [VN: **PRTIMPORTANT**] It made me feel like part of something important	☐	☐	☐	☐	☐	☐
317. [VN: **PRTHELPCOMMU**] It made me feel like I am helping my community	☐	☐	☐	☐	☐	☐
318. [VN: **PRTTALKEXP**] It helped me to have someone to talk to about my experiences	☐	☐	☐	☐	☐	☐
319. [VN: **PRTFEELHELP**] It made me feel like I am helping other young men like myself	☐	☐	☐	☐	☐	☐
320. [VN: **PRTKNOWPPL**] It helped me to know that people care about other young men like myself	☐	☐	☐	☐	☐	☐
321. [VN: **PRTQREFLECT**] Answering the questions helped me reflect on who I am	☐	☐	☐	☐	☐	☐
322. [VN: **PRTSUPPORT**] Participating in the study made me feel supported	☐	☐	☐	☐	☐	☐
323. [VN: **PRTBEHAVIOR**] Participating in the study helped me to think about my behavior	☐	☐	☐	☐	☐	☐

VN: **PRTPOSIMPACT**

324. Has your participation in this study had a positive or beneficial impact on your life?

1 ☐ Yes → **GO TO PRTIMPROVE**

2 ☐ No → **IF VISIT = 4.0 (WEEK 26) END QUESTIONNAIRE. GO TO END SCREEN. IF VISIT = 6.0 (WEEK 52) GO TO PRTRIGHTDECISION**

98 ☐ Don't Know → **IF VISIT = 4.0 (WEEK 26) END QUESTIONNAIRE. GO TO END SCREEN. IF VISIT = 6.0 (WEEK 52) GO TO PRTRIGHTDECISION**

99 ☐ No answer → **IF VISIT = 4.0 (WEEK 26) END QUESTIONNAIRE. GO TO END SCREEN. IF VISIT = 6.0 (WEEK 52) GO TO PRTRIGHTDECISION**

SHOW PRTIMPROVE PRTOTHERBENEFIT IF PRTPOSIMPACT = 1 (YES)

Because of your participation in this study, have you experienced…?	Yes = 1	No = 2	No answer = 99
325. [VN: **PRTIMPROVE**] Employment improvement – for example, you found a job or your performance improved at your current job	☐	☐	☐
326. [VN: **PRTFINANCIAL**] Financial improvement – for example, you saved financial incentives that you received as part of the study	☐	☐	☐
327. [VN: **PRTKNOWLEDGE**] Knowledge improvement – for example, you gained knowledge about HIV prevention or risks associated with alcohol or drug use, or you learned about your HIV status	☐	☐	☐
328. [VN: **PRTLIFE**] Life improvement – for example, you feel that your life has improved as a result of being in the study or it has returned to normal	☐	☐	☐
329. [VN: **PRTPHYSHEALTH**] Physical health improvement – for example, you have experienced an improvement in your physical health	☐	☐	☐
330. [VN: **PRTMENTALHEALTH**] Mental health improvement – for example, you feel less stressed or more self-confident	☐	☐	☐
331. [VN: **PRTRELATIONSHIPS**] Improved relationships – for example, your relationships with family or friends have improved	☐	☐	☐
332. [VN: **PRTREDUCEALC**] Reduction in alcohol or drug use – for example, you have reduced your frequency or dosage or alcohol/drug use, detoxed, or stopped using all together	☐	☐	☐

VN: **PRTOTHBENEFIT**

333. Have you experienced any other benefits due to participating in this study? **[OPEN TEXT]**

____

2 ☐ No

99☐ No answer

IF VISIT = 4.0 (WEEK 26) END QUESTIONNAIRE. GO TO END SCREEN.

SHOW PRTRIGHTDECISION – PRTLGBT IF VISIT = 6.0 (WEEK 52)

If you look back at the whole study and think about your decision to participate in this study, would you agree or disagree with the following statements:

	Disagree strongly = 1	Disagree = 2	Neutral = 3	Agree = 4	Agree strongly = 5	No answer = 99
334. **[VN: PRTRIGHTDECISION]** It was the right decision	☐	☐	☐	☐	☐	☐
335. **[VN: PRTREGRETCHOICE]** I regret the choice that was made	☐	☐	☐	☐	☐	☐
336. **[VN: PRTSAMECHOICE]** I would go for the same choice if I had to do it over again	☐	☐	☐	☐	☐	☐
337. **[VN: PRTHARMCHOICE]** The choice did me a lot of harm	☐	☐	☐	☐	☐	☐
338. **[VN: PRTWISEDECISION]** The decision was a wise one	☐	☐	☐	☐	☐	☐

339. **VN: PRTPARTICIPATION**
If in the future we would like to do a study like this one, would you be interested in participating again?
1☐ Yes
2☐ No
98☐ Don't know
99☐ No answer

340. **VN: PRTCONTACT**
Would we be allowed to contact you again?
1☐ Definitely
2☐ Probably
3☐ Probably not
4☐ Definitely not
98☐ Don't know
99☐ No answer

341. **VN: PRTLGBT**
Would you encourage other people who are gay, bisexual, or transgender to participate in such a study or would you discourage them?
1☐ Strongly encourage
2☐ Encourage
3☐ Discourage
4☐ Strongly discourage
98☐ Don't know
99☐ No answer

END SCREEN
Thank you for your participation in this interview!
