# Supplementary material for: HIV incidence in a multinational cohort of men and transgender women who have sex with men in sub-Saharan Africa: Findings from HPTN 075
Source: PLoS One. 2021 Feb 25;16(2):e0247195. doi: 10.1371/journal.pone.0247195 (PMC7906338; doi:10.1371/journal.pone.0247195)
Supplement: S1 File — (DOCX) [file pone.0247195.s006.docx]

| **Variable Names** | **Format** | **Description** |  |
| --- | --- | --- | --- |
| Uid |  | Participant uid | |
| start | Date7. | visit date of last visit (if at enrollment, then the current visit date) | |
| Stop | Date7. | Visit date at current visit | |
| Event2 | 0= “Negative”  1= “Positive” | HIV event by current visit | |
| Site_mala | 1 = “Malawi”  2 = “Kenya”  3 = “Cape Town”  4 = “Soweto”  ; | study site | |
| sexwsex | 1=”No”  3=”Yes”  ; | Ever sex with women | |
| Age_grp | 1=”Age 18-24”  2=”25+”  ; | Participant age group |  |
| Demeduc_r | 1= “Low”  2= “Middle”  3= “High”  ; | Education level |  |
| demorient | 1= “Men Only”  2= “Men and women”  ; | Sexual Orientation;  if idattract=5 then demorient=1;  else if idattract in (1 2 3 4) then demorient=2;  else if idattract=99 then demorient=.; |  |
| Sexident | 1= “Gay”  2= “Bisexual and other”  ; | Sexual identity;  if idsexual=1 then sexident=1;  else if idsexual>1 then sexident=2; |  |
| demtrans | 1= “No”  2= “Yes”  ; | Transgender |  |
| Shmedcir_r | 1= “No”  2= “Yes”  ; | Medical Circumcision |  |
| shexchng_r | 1= “No”  2= “Yes”  ; | Transactional sex (ever) |  |
| shafter_r | 1= “No”  2= “Yes”  ; | Forced sex (ever) |  |
| sexwsex | 1= “No”  3= “Yes”  ; | Sex with women (ever) |  |
| homo_exp_cat | 1= “None”  2= “Any”  ; | Homophobic experience;  if stighara>0 or stigbeat>0 or stigblack>0 or stiglost>0 or stigdisc>0 then  homo_exp=sum(STIGHARA=1, STIGBEAT=1, STIGBLACK=1, STIGLOST=1, STIGDISC=1);  /*categorize the numerical covariates*/  if homo_exp=0 then homo_exp_cat=1;  else if homo_exp>0 then homo_exp_cat=2; |  |
| neg_homo_cat | 1= “Few”  2= “Many”  ; | Negative feelings about homosexuality;  if IDGLAD<99 & IDGLAD>0 THEN IDGLADR=5-IDGLAD;  else IDGLADr=IDGLAD;  do i=1 to 4;  if neg{i}=99 then neg{i}=.;  end;  neg_homo=sum(IDDECIDE, IDISLIKE, IDWISH, IDGLADr)/sum((IDDECIDE^<.z), (IDISLIKE^<.z),(IDWISH^<.z), (IDGLADr^<.z));  if sum((IDDECIDE^<.z), (IDISLIKE^<.z),(IDWISH^<.z), (IDGLADr^<.z))<2 then neg_homo=.;  if 0<=neg_homo<=2 then neg_homo_cat=1;  else if neg_homo>2 then neg_homo_cat=2; |  |
| idhidden_cat_r | 1= “Low”  2= “High”  ; | Concealment of same-sex attraction;  if idhidden in (98, 99) then idhidden=.;  if idhidden2 in (98, 99) then idhidden2=.;  IDHIDDENALL=sum(5-IDHIDDEN, 5-IDHIDDEN2);  if IDHIDDENALL>2 then idhidden_cat_r=1;  else if 0<=IDHIDDENALL<=2 then idhidden_cat_r=2; |  |
| sphmany_c | 1= “0-1”  2= “2”  3= “3+”  ; | #of partners |  |
| sexaodself_c | 0= “0”  1= “1+”  ; | #of partners that when under the influence  sexaodself=sum(sp1aodself in (2 3 4 5 7), sp2aodself in (2 3 4 5 7), sp3aodself in (2 3 4 5 7)); |  |
| timesunrec_c | 0= “0”  1= “1+”  ; | #of time had unprotected anal sex  if sp1gender ^in (1 3 5) or sp1anal ^=1 then count1=0;  else if sp1anal=1 then do;  if sp1times=1 & sp1asrasc in (2 3) then count1=1;  else if (sp1times=1 & sp1asrasc=1) or (sp1times=2 & sp1unprot=99) then count1=0;  else if sp1times=2 then count1=sp1unprot_text;  end;  if sp2gender ^in (1 3 5) or sp2anal^=1 then count2=0;  else if sp2anal=1 then do;  if sp2times=1 & sp2asrasc in (2 3) then count2=1;  else if (sp2times=1 & sp2asrasc=1) or (sp2times=2 & sp2unprot=99) then count2=0;  else if sp2times=2 then count2=sp2unprot_text;  end;  if sp3gender ^in (1 3 5) or sp3anal^=1 then count3=0;  else if sp3anal=1 then do;  if sp3times=1 & sp3asrasc in (2 3) then count3=1;  else if (sp3times=1 & sp3asrasc=1) or (sp3times=2 & sp3unprot=99) then count3=0;  else if sp3times=2 then count3=sp3unprot_text;  end;  if sphmany_text<=0 then timesunrec=0;  else if sphmany_text=1 then timesunrec=count1;  else if sphmany_text=2 then timesunrec=count1+count2;  else if sphmany_text>=3 then timesunrec=count1+count2+count3; |  |
| maleunrec_c | 0= “0”  1= “1”  2= “2+”  ; | #of male partners with unprotected anal sex;  if sp1gender ^in (1 3 5) or sp1anal^=1 then countm1=0;  else if sp1anal=1 then do;  if (sp1asrasc in (2 3)) or sp1unprot_text>0 then countm1=1;  else if sp1asrasc=1 or sp1unprot_text=0 or sp1unprot=99 then countm1=0;  end;  if sp2gender ^in (1 3 5) or sp2anal^=1 then countm2=0;  else if sp2anal=1 then do;  if (sp2asrasc in (2 3)) or sp2unprot_text>0 then countm2=1;  else if sp2asrasc=1 or sp2unprot_text=0 or sp2unprot=99 then countm2=0;  end;  if sp3gender ^in (1 3 5) or sp3anal^=1 then countm3=0;  else if sp3anal=1 then do;  if (sp3asrasc in (2 3)) or sp3unprot_text>0 then countm3=1;  else if sp3asrasc=1 or sp3unprot_text=0 or sp3unprot=99 then countm3=0;  end;  if sphmany_text<=0 then maleunrec=0;  else if sphmany_text=1 then maleunrec=countm1;  else if sphmany_text=2 then maleunrec=countm1+countm2;  else if sphmany_text>=3 then maleunrec=countm1+countm2+countm3; |  |
| timesunins_c | 0= “0”  1= “1+”  ; | #of time had unprotected insertive anal sex;  if sp1gender ^in (1 3 5) or sp1insert^=1 then counti1=0;  else if sp1insert=1 then do;  if sp1times=1 & sp1asiasc in (2 3) then counti1=1;  else if (sp1times=1 & sp1asiasc=1) or (sp1times=2 & sp1inprot=99) then counti1=0;  else if sp1times=2 then counti1=sp1inprot_text;  end;  if sp2gender ^in (1 3 5) or sp2insert^=1 then counti2=0;  else if sp2insert=1 then do;  if sp2times=1 & sp2asiasc in (2 3) then counti2=1;  else if (sp2times=1 & sp2asiasc=1) or (sp2times=2 & sp2inprot=99) then counti2=0;  else if sp2times=2 then counti2=sp2inprot_text;  end;  if sp3gender ^in (1 3 5) or sp3insert^=1 then counti3=0;  else if sp3insert=1 then do;  if sp3times=1 & sp3asiasc in (2 3) then counti3=1;  else if (sp3times=1 & sp3asiasc=1) or (sp3times=2 & sp3inprot=99) then counti3=0;  else if sp3times=2 then counti3=sp3inprot_text;  end;  if sphmany_text<=0 then timesunins=0;  else if sphmany_text=1 then timesunins=counti1;  else if sphmany_text=2 then timesunins=counti1+counti2;  else if sphmany_text>=3 then timesunins=counti1+counti2+counti3; |  |
| maleunins_c | 0= “0”  1= “1”  2= “2+”  ; | #of male with unprotected insertive anal sex;  if sp1gender ^in (1 3 5) or sp1insert^=1 then countim1=0;  else if sp1insert=1 then do;  if (sp1asiasc in (2 3)) or sp1inprot_text>0 then countim1=1;  else if sp1asiasc=1 or sp1inprot_text=0 or sp1inprot=99 then countim1=0;  end;  if sp2gender ^in (1 3 5) or sp2insert^=1 then countim2=0;  else if sp2insert=1 then do;  if (sp2asiasc in (2 3)) or sp2inprot_text>0 then countim2=1;  else if sp2asiasc=1 or sp2inprot_text=0 or sp2inprot=99 then countim2=0;  end;  if sp3gender ^in (1 3 5) or sp3insert^=1 then countim3=0;  else if sp3insert=1 then do;  if (sp3asiasc in (2 3)) or sp3inprot_text>0 then countim3=1;  else if sp3asiasc=1 or sp3inprot_text=0 or sp3inprot=99 then countim3=0;  end;  if sphmany_text<=0 then maleunins=0;  else if sphmany_text=1 then maleunins=countim1;  else if sphmany_text=2 then maleunins=countim1+countim2;  else if sphmany_text>=3 then maleunins=countim1+countim2+countim3; |  |
| any_rec | 0 = “Negative”  1= “Positive”  ; | any rectal GC/CT;  IF CT_rec="POSITIVE" or GC_rec="POSITIVE" then any_rec=1;  else any_rec=0;  if CT_rec=" " & GC_rec=" " then any_rec=.; |  |
| any_oral | 0 = “Negative”  1= “Positive”  ; | any oral GC/CT;  IF CT_oral="POSITIVE" or GC_oral="POSITIVE" then any_oral=1;  else if GC_oral=" " & CT_oral=" " then any_oral=.;  else any_oral=0; |  |
| any_urine | 0 = “Negative”  1= “Positive”  ; | any uretral GC/CT;  if STIgnunp=2 or STIctunp=2 then any_urine=1;  else if STIgnunp<.z & STIctunp<.z then any_urine=.;  else any_urine=0; |  |
| syph | 0 = “Negative”  1= “Positive”  ; | any syphilis;  if STItrenr=2 or STInrtre=2 then syph=1;  else if STItrenr<.z & STInrtre<.z then syph=.;  else syph=0; |  |
| any_hbv | 0 = “Negative”  1= “Positive”  ; | any HBV;  if STIhep<.z & STIhsab<.z & STIhcab=<.z then any_hbv=.;  else if STIhep=1 & STIhsab=2 & STIhcab=2 then any_hbv=1;  else any_hbv=0; |  |
